# Ecofriendly Upcycling of Poly(vinyl chloride) Waste Plastics into Precious Metal Adsorbents

**DOI:** 10.1002/advs.202503157

**Published:** 2025-05-09

**Authors:** Seung Su Shin, Seungho Lee, Sung‐Joon Park, Hansoo Kim, Juyeon Choi, Wangyun Won, Jung‐Hyun Lee

**Affiliations:** ^1^ Department of Chemical and Biological Engineering Korea University 145 Anam‐ro, Seongbuk‐gu Seoul 02841 Republic of Korea; ^2^ Department of Polymer Science and Engineering Kyungpook National University 80 Daehak‐ro, Buk‐gu Daegu 41566 Republic of Korea

**Keywords:** eco‐friendly upcycling, hydrazine functionalization, poly(vinyl chloride), precious metal adsorbent, waste plastic upcycling

## Abstract

Global interest in the recycling of precious metals (PMs) in various industrial sectors has spurred the exploration of high‐performance PM adsorbents. Unfortunately, many adsorbents exhibit unsatisfactory PM adsorption performance and require complex fabrication protocols and toxic chemicals. Hence, further development of simple, efficient, and eco‐friendly adsorbents is necessary. Herein, poly(vinyl chloride) (PVC) waste plastics are simply transformed into high‐performance PM adsorbents via benign solvent treatment and hydrazination. The resultant hydrazine‐functionalized PVC (h‐PVC) plastic can effectively recover gold, palladium, and platinum from real‐world leachates owing to its combined reduction and chemisorption mechanisms. The PM‐adsorbed h‐PVC plastic can be regenerated, calcined into high‐purity PMs, or directly employed as a catalyst, demonstrating its practical feasibility. Techno‐economic and life‐cycle assessments reveal that the h‐PVC plastic‐utilizing industrial‐scale recovery of gold from electronic waste is cost‐competitive and environmentally advantageous. The strategy supports environmental and sustainable technologies by enabling the sustainable maintenance of carbon and PM resources and provides an efficient and sustainable method for fabricating advanced adsorbent materials.

## Graphical abstract

Poly(vinyl chloride) waste plastics are simply transformed into high‐performance precious metal (PM) adsorbents via a facile and green hydrazine‐functionalization process. The resultant adsorbent effectively and selectively recovers PM from real‐world leachates via its combined chemisorption and reduction mechanisms. Its practical feasibility and potential for industrial‐scale application are also comprehensively demonstrated.

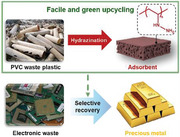

## 1 Introduction

Precious metals (PMs) (e.g., gold (Au), palladium (Pd), and platinum (Pt)) are valuable elements with unique properties that are beneficial for diverse industrial applications, including electronics, catalysts, batteries, and solar and fuel cells.^[^ ^1^, ^2^, ^3^ ^]^ However, PMs are scarce; thus, the development of technologies to effectively recover PMs from end‐of‐life electronic waste and spent catalysts is an active research area.^[^ ^4^ ^]^

Among several technologies for PM recovery, including adsorption,^[^ ^5^ ^]^ extraction,^[^ ^6^ ^]^ electrodialysis,^[^ ^7^ ^]^ and precipitation,^[^ ^8^ ^]^ adsorption has received significant interest owing to its high efficiency and simple operation.^[^ ^5^ ^]^ Various adsorbents, including polymers, activated carbons, and organic and inorganic nanoparticles, have been explored for PM recovery.^[^ ^5^, ^9^, ^10^, ^11^ ^]^ Polymer adsorbents have attracted particular attention owing to their high PM adsorption capacity and facile modification.^[^ ^12^ ^]^ Amine‐functionalized polymers (e.g., polyethylene imine) are widely employed to adsorb PM ions because of their favorable chemical interactions (i.e., electrostatic interaction and chelation) with PM species.^[^ ^13^ ^]^ However, they display inherently low PM adsorption capacity and selectivity because their PM adsorption relies mainly on chemical interactions.^[^ ^14^, ^15^ ^]^ Hydrazide‐functionalized polymers were recently proven to be more effective at recovering PMs than amine polymers via their strong chemical interactions and high reduction ability.^[^ ^16^ ^]^ Unfortunately, because most polymer adsorbents must be supported on substrates for their facile recovery, their adsorption performance is unsatisfactory.^[^ ^17^ ^]^ Furthermore, polymer adsorbents often require complex fabrication protocols and/or toxic chemicals/solvents,^[^ ^18^ ^]^ which can increase production costs and raise environmental concerns. These limitations have fueled the demand for facile, economic, and environmentally sustainable methods to fabricate high‐performance polymeric PM adsorbents.^[^ ^10^ ^]^

Herein, we present the facile upcycling of poly(vinyl chloride) (PVC) waste plastics into highly effective and easily recoverable PM adsorbents via benign solvent treatment followed by hydrazination. PVC, one of the commercial commodity plastics, contains reactive chlorine groups bonded to a polyethylene backbone. PVC plastics typically contain plasticizers (e.g., di‐2‐ethylhexyl phthalate) to improve their processibility.^[^ ^19^ ^]^ Although various functional groups, including amine, bipyridine, and imidazole, have been introduced to PVC for use as PM adsorbents, they resulted in unsatisfactory PM adsorption capacity and selectivity owing to their limited adsorption mechanisms.^[^ ^20^, ^21^, ^22^ ^]^ Moreover, previous strategies have fabricated PVC adsorbents by chemically modifying pure PVC polymer powder or physically treating (e.g., grounding and separation) PVC waste plastics prior to chemical modification to avoid the unwanted effect of plasticizers.^[^ ^20^, ^21^ ^]^ To overcome the drawbacks of previous strategies, we aimed to directly upcycle various pristine PVC waste plastics into high‐performance PM adsorbents by introducing porous structures and new hydrazine functional groups in an eco‐friendly manner. The simple treatment of a PVC waste plastic with benign solvents (dimethyl sulfoxide (DMSO) and ethanol (EtOH)) effectively removed the plasticizer and induced phase separation, yielding a highly porous, deplasticized PVC (d‐PVC) plastic. Subsequent hydrazination resulted in a porous, hydrazine‐functionalized PVC (h‐PVC) plastic. The resultant h‐PVC plastic featured high PM adsorption ability owing to its highly reducing hydrazine groups and could be readily collected because of its macroscale size.

To verify the validity of our strategy, we first synthesized a model h‐PVC polymer by hydrazinating a PVC polymer and then compared its PM adsorption performance and mechanism with those of commercial reducing agents (i.e., hydrazine and sodium borohydride (NaBH_4_)). Based on this model study, the PM adsorption performance and selectivity of the h‐PVC plastic were evaluated using real‐world leachates. The practical feasibility of the h‐PVC plastic was demonstrated by regenerating, refining, and directly utilizing the PM‐adsorbed h‐PVC (PM@h‐PVC) plastic. We also performed techno‐economic and life‐cycle assessments (TEA and LCA, respectively) for integrated processes featuring the h‐PVC plastic adsorbent for Au recovery from electronic waste.

## 2 Results and Discussion

### 2.1 Properties and PM Adsorption Mechanism of the h‐PVC Polymer

**Figure 1:**
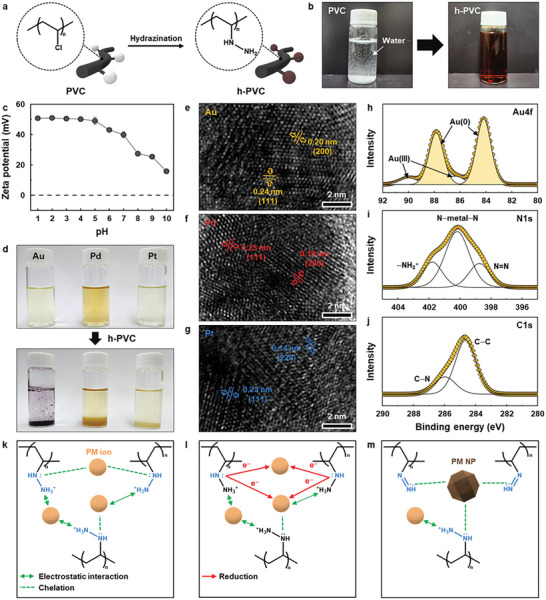
a) Schematic of the synthesis of the h‐PVC polymer via hydrazination. b) Photographs of the PVC and h‐PVC polymer aqueous solutions. c) Zeta potentials of the h‐PVC polymer as a function of the solution pH. d) Photographs of PM (Au, Pd, and Pt, 200 mg L^−1^) aqueous solutions (pH = 2) before (top) and after (bottom) the introduction of the h‐PVC polymer (0.2 g L^−1^). e–g) High‐resolution TEM images of the PM@h‐PVC precipitates: e) Au, f) Pd, and g) Pt. h–j) Deconvoluted h) Au4f, i) N1s, and j) C1s XPS peaks of the Au@h‐PVC precipitate. The PM@h‐PVC precipitates were collected by PSU membrane filtration after PM (200 mg L^−1^) aqueous solutions (pH = 2) containing the h‐PVC polymer (0.2 g L^−1^) were shaken for 3 h. k–m) Proposed PM adsorption mechanism of the h‐PVC polymer. Data represents the mean ± standard deviation (*n* = 3).

The h‐PVC polymer was synthesized by completely converting the chlorine groups of a PVC polymer into hydrazine groups via hydrazination at 80 °C for 5 d (**Figure** **1**a; Figures S1–S3, Supporting Information). Whereas the pristine PVC polymer was white and water‐insoluble, the h‐PVC polymer was brown and highly water‐soluble (solubility: 5 w/v.%) owing to its hydrophilic hydrazine groups (Figure 1b; Figure S4, Supporting Information). The h‐PVC polymer bearing abundant cationic hydrazine groups exhibited a strong positive charge, which was intensified at a low pH owing to the protonation of the hydrazine groups (Figure 1c).^[^ ^23^ ^]^

The introduction of the h‐PVC polymer to acidic PM aqueous solutions led to rapid precipitation, presumably owing to the reduction of the PM ions by the highly reducing hydrazine groups of the polymer (Figure 1d). Because the large PM@h‐PVC precipitates formed were easily collected by membrane filtration (Figure S5, Supporting Information), the h‐PVC polymer may potentially be used as a standalone adsorbent. PM reduction by the h‐PVC polymer was evidenced by the characteristic red–purple color and UV–vis spectrum of Au metal nanoparticles (NPs)^[^ ^16^ ^]^ observed in an h‐PVC‐containing Au aqueous solution (Figure 1d; Figure S6, Supporting Information). Transmission electron microscopy (TEM) and X‐ray diffraction (XRD) also identified the crystal lattice structures of PM NPs in the PM@h‐PVC precipitates^[^ ^16^ ^]^ (Figure 1e–g; Figures S7 and S8, Supporting Information). X‐ray photoelectron spectroscopy (XPS) of the PM@h‐PVC precipitates revealed both ionic and metallic PM (PM(0)) peaks (Figure 1h; Figures S9 and S10, Supporting Information). The integrated area fraction of the PM(0) peak relative to the ionic PM peak decreased in the order of Au (89%) > Pt (80%) > Pd (29%), positively correlating with the reduction potentials of these ions (AuCl_4_^−^ > PtCl_6_^2−^ > PdCl_4_^2−^).^[^ ^24^ ^]^ Whereas the h‐PVC polymer exhibited two deconvoluted N1s peaks at 399.6 (secondary amine, ─NH─) and 401.5 (protonated primary amine, ─NH_3_^+^) eV (Figure S2a, Supporting Information), the PM@h‐PVC precipitates displayed three N1s peaks at 398.7 (N═N), 400.2 (N─metal─N), and 401.9 (─NH_3_^+^) eV (Figure 1i; Figure S11, Supporting Information).^[^ ^25^ ^]^ The higher binding energy of the ─NH_3_^+^ peak for the PM@h‐PVC precipitates supports the occurrence of electrostatic interactions between their ─NH_3_^+^ groups and PM ions.^[^ ^25^ ^]^ The appearance of the N═N peak for the PM@h‐PVC precipitates indicates that some (44–50%) of the protonated hydrazine (─NHNH_3_^+^) groups of h‐PVC were oxidized to diazene groups by reducing PM ions (Table S1, Supporting Information).^[^ ^26^ ^]^ Moreover, the presence of the N─metal─N peak accounted for chelate formation between protonated hydrazine/diazene groups and the PM ions/NPs. No distinct difference in the C1s peak was observed between the h‐PVC polymer and PM@h‐PVC precipitates (Figure 1j; Figures S2b and S12, Supporting Information).

Given the above results, we can reasonably speculate that the protonated h‐PVC polymer electrostatically attracts anionic PM species in acidic solutions via its ─NH_3_^+^ groups^[^ ^27^ ^]^ and forms chelates with these ions via its unshared electron‐bearing hydrazine ─NH─ groups (Figure 1k).^[^ ^5^ ^]^ Subsequently, the protonated hydrazine groups of the h‐PVC polymer reduce a fraction of adsorbed PM ions to NPs by donating electrons^[^ ^16^ ^]^ while being oxidized to diazene groups (Figure 1l,m; Note S1, Supporting Information).^[^ ^26^ ^]^ Progressive PM reduction promotes the formation of PM NPs, which are chelated with neighboring diazene and/or hydrazine ─NH─ groups bearing unshared electrons (Figure 1m), consequently forming large PM@h‐PVC precipitates.^[^ ^16^ ^]^

### 2.2 PM Adsorption Performance of the h‐PVC Polymer

**Figure 2:**
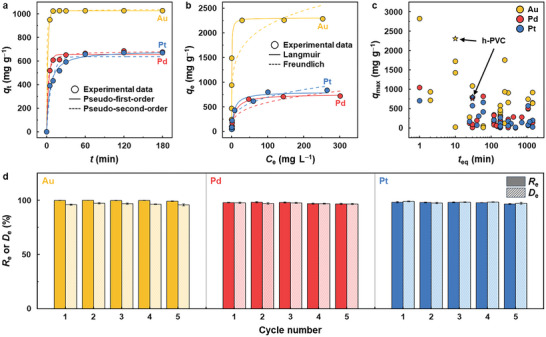
a) PM adsorption kinetics (PM adsorption capacity (*q*_t_) as a function of the contact time (*t*)) of the h‐PVC polymer and their fits to established kinetics models (h‐PVC polymer dose = 0.2 g L^−1^, initial PM ion concentration (*C*_i_) = 200 mg L^−1^, pH = 2). b) PM adsorption isotherms (equilibrium PM adsorption capacity (*q*_e_) as a function of the equilibrium PM ion concentration (*C*_e_)) of the h‐PVC polymer and their fits to established isotherm models (h‐PVC polymer dose = 0.2 g L^−1^, pH = 2, contact time = 3 h). c) PM adsorption performance (maximum PM adsorption capacity (*q*_max_) and equilibrium time (*t*_eq_)) of the h‐PVC polymer and other reported PM adsorbents. d) PM recovery (*R*_e_) and desorption (*D*_e_) efficiency of the h‐PVC polymer as a function of the adsorption–desorption cycle number (h‐PVC polymer dose = 0.2 g L^−1^, *C*_i_ = 10 mg L^−1^, pH = 2, contact time = 3 h). Data represents the mean ± standard deviation (*n* = 3).

The PM adsorption performance of the h‐PVC polymer was characterized at pH 2, which corresponds to the pH range (0–2) of typical PM leachates;^[^ ^27^, ^28^ ^]^ maximum PM recovery efficiency (*R*_e_) was achieved at this pH (Note S2 and Figure S13, Supporting Information). The h‐PVC polymer rapidly adsorbed PM ions with a short equilibrium time (*t*_eq_, 10 min for Au, and 30 min for Pd and Pt) when 95% of its equilibrium adsorption capacity (*q*_e_) was reached (**Figure** **2**a). The adsorption kinetics data of the h‐PVC polymer fit the pseudo‐second‐order model well (Figure 2a; Table S2, Supporting Information), indicating that PM ions are chemisorbed by the h‐PVC polymer via electrostatic interaction and chelation.^[^ ^29^ ^]^ Furthermore, the adsorption isotherm data of the h‐PVC polymer fit the Langmuir model well (Figure 2b; Table S3, Supporting Information), suggesting that it adsorbs PM ions through monolayer formation.^[^ ^30^ ^]^ Anionic PM species are chemisorbed by the protonated hydrazine groups of the h‐PVC polymer through monolayer formation and then reduced to their NP forms. Because NP growth is enabled by the post‐transition (collective reduction) of PM ions chemisorbed onto neighboring adsorptive site, PM adsorption onto the h‐PVC polymer (chemisorption and subsequent reduction) can be depicted by the monolayer chemisorption mechanism.^[^ ^16^, ^31^ ^]^ The maximum PM adsorption capacities (*q*_max_) of the h‐PVC polymer determined for Au, Pt, and Pd were 2304, 803, and 759 mg g^−1^, respectively (Table S3, Supporting Information), corresponding to the reduction potentials of these ions. This result implies the critical role of the reduction mechanism of the h‐PVC polymer in its PM adsorption capacity. Compared with most reported adsorbents, the h‐PVC polymer exhibited a significantly higher *q*_max_ and PM adsorption rate (Figure 2c; Table S4, Supporting Information). In water, h‐PVC polymer chains are highly dissolved, well dispersed, and flexible. Particularly, in the acidic aqueous solution, h‐PVC polymer chains are highly stretched owing to strong intramolecular electrostatic repulsion between their positively charged hydrazine groups. Hence, combined with its stretched chain conformation, the high chain dispersion, and mobility of the h‐PVC polymer in the acidic PM solution would allow for its rapid and direct interaction with PM ions owing to marginal mass transfer resistance, resulting in its fast PM adsorption kinetics. Furthermore, compared with the reducing agents (hydrazine and NaBH_4_), the h‐PVC polymer displayed a higher *R*_e_ for all PMs investigated at the same dose (Note S3 and Figures S14 and S15, Supporting Information). The exceptional PM adsorption performance of the h‐PVC polymer can be attributed to its capacity for chemisorption through electrostatic and chelation interactions combined with its strong ability to reduce PM ions.^[^ ^16^ ^]^ Interestingly, unlike hydrazine, the h‐PVC polymer effectively reduced Pt ions even at a low dose of 0.2 g L^−1^ (Figures S14–S16, Supporting Information), clearly demonstrating its advantage over hydrazine. We speculate that locally concentrated hydrazine groups confined to the polymer backbone collectively boost the PM reduction ability of the h‐PVC polymer.^[^ ^16^ ^]^ Moreover, unlike the reducing agents, the h‐PVC polymer produced large and easily collectible PM@h‐PVC precipitates, from which the h‐PVC polymer could be regenerated using PM desorption agents (hydrochloric acid (HCl) and thiourea).^[^ ^27^ ^]^ The h‐PVC polymer maintained its high *R*_e_ (>95%) and desorption efficiency (*D*_e_, >95%) for the three PMs over five adsorption–desorption cycles (Figure 2d), demonstrating its excellent reusability. The high reusability of the h‐PVC polymer can be attributed to the effective regeneration of hydrazine groups from their oxidized diazene groups during the repeated long‐term recycling process (Figure S17, Supporting Information).

### 2.3 Fabrication and Characterization of the h‐PVC Plastic

**Figure 3:**
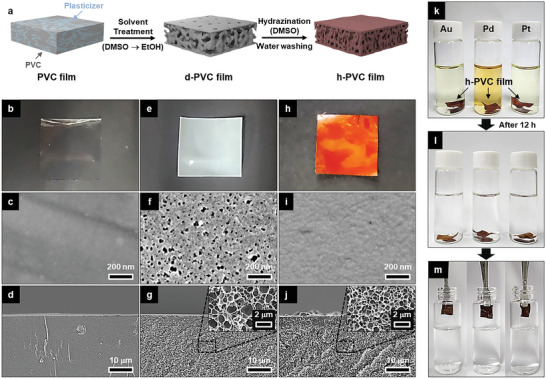
a) Schematic of the fabrication of the h‐PVC plastic film via benign solvent treatment and subsequent hydrazination. b,e,h) Photographs and c,f,i) surface and d,g,j) cross‐sectional SEM images of the b–d) pristine PVC, e–g) d‐PVC, and h–j) h‐PVC films. k–m) Photographs illustrating the PM adsorption and collection processes of the h‐PVC film.

Although the h‐PVC polymer can be employed as a standalone PM adsorbent, its key beneficial attributes were further exploited to upcycle PVC waste plastics into PM adsorbents. A commercially available PVC plastic film was transformed into a porous h‐PVC film via benign solvent treatment (DMSO followed by EtOH) and subsequent hydrazination (**Figure** **3**a). DMSO, which is a good solvent for PVC and the plasticizer,^[^ ^32^ ^]^ effectively swelled the PVC network while dissolving the plasticizer. The subsequent immersion of the DMSO‐swollen PVC film into EtOH, which is a poor solvent for PVC but a good solvent for the plasticizer,^[^ ^32^ ^]^ caused non‐solvent‐induced phase separation^[^ ^33^, ^34^ ^]^ while completely removing the plasticizer, forming a highly porous d‐PVC film. Hydrazination of the d‐PVC film in DMSO followed by water rinsing yielded the porous h‐PVC film.

Whereas the pristine PVC plastic film was transparent and had a completely dense structure (Figure 3b–d), the d‐PVC film was opaque white owing to its porous structure (Figure 3e–g). The d‐PVC film had an asymmetric pore structure comprising a dense microporous (pore size: ≤100 nm) surface layer (thickness: ≈5 µm) and a macroporous (pore size: ≤1 µm) inner layer (Figure 3f,g; Figure S18, Supporting Information). This unique structure may have resulted from the rapid phase separation occurring near the surface of the d‐PVC film during its formation.^[^ ^34^ ^]^ Marginal detection of the characteristic Fourier transform infrared peak of the plasticizer (i.e., di‐2‐ethylhexyl terephthalate) for the d‐PVC film (Figures S19 and S20, Supporting Information) confirmed the effective removal of the plasticizer from the PVC film by solvent treatment. The h‐PVC film exhibited a characteristic brown color (Figure 3h), which intensified as the hydrazination time increased owing to the increase in hydrazination degree (Figure S21, Supporting Information). The porosity of the h‐PVC film gradually decreased with increasing hydrazination time up to 24 h and then plateaued (Figure S22, Supporting Information). The hydrazination time was optimized at 24 h, at which the PM adsorption capacity (*q*_e_) and kinetics of the resultant h‐PVC film were saturated (Figures S23 and S24, Supporting Information). The optimized h‐PVC film exhibited ∼65% conversion of its chlorine to hydrazine groups (Figures S20 and S25, Supporting Information) and was used in subsequent experiments. Compared with the d‐PVC film, the h‐PVC film exhibited a similar asymmetric structure but smaller pores and lower overall porosity (≈52%) (Figure 3i,j; Figures S18 and S26, Supporting Information). Exposure of the h‐PVC film to water, which is less compatible with h‐PVC than DMSO, during the washing step after hydrazination presumably resulted in slight pore shrinkage. Unlike the negatively charged PVC film,^[^ ^35^ ^]^ the h‐PVC film exhibited a strong positive charge at pH <10 owing to its abundant hydrazine groups (Figure S27, Supporting Information).^[^ ^23^ ^]^ The h‐PVC film effectively adsorbed PM species in acidic PM aqueous solutions (Figure 3k,l) and was readily collected by hand owing to its macroscale size (Figure 3m).

### 2.4 PM Adsorption Mechanism and Performance of the h‐PVC Plastic

**Figure 4:**
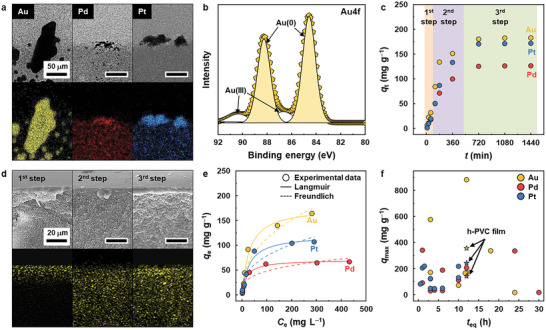
a) Cross‐sectional TEM (top) and corresponding EDS (bottom) images of the PM@h‐PVC plastic films. The PM@h‐PVC films were collected using tweezers after PM (200 mg L^−1^) aqueous solutions (pH = 2) containing the h‐PVC film (1.0 g L^−1^) were shaken for 12 h. b) Deconvoluted Au4f XPS peak of the Au@h‐PVC film. c) PM adsorption kinetics (*q*_t_ as a function of *t*) of the h‐PVC film (h‐PVC film dose = 1.0 g L^−1^, *C*_i_ = 200 mg L^−1^, pH = 2). d) Cross‐sectional SEM (top) and corresponding EDS (bottom) images of the Au@h‐PVC film over three‐step adsorption (1^st^ step at 10 min, 2^nd^ step at 2 h, and 3^rd^ step at 12 h). e) PM adsorption isotherms (*q*_e_ as a function of *C*_e_) of the h‐PVC film and their fits to established isotherm models (h‐PVC film dose = 1.0 g L^−1^, pH = 2, contact time = 12 h). f) PM adsorption performance (*q*_max_ and *t*_eq_) of the h‐PVC film and other reported macroscale PM adsorbents.

The cross‐sectional TEM and EDS images and XRD patterns of the PM@h‐PVC films corresponded to those of metallic PM NPs (**Figure** **4**a; Figure S28, Supporting Information).^[^ ^36^ ^]^ The PM@h‐PVC films also exhibited XPS profiles similar to those of the PM@h‐PVC polymer precipitates but had lower metallic PM fractions because the hydrazine group density of the h‐PVC film is lower than that of the h‐PVC polymer (Figure 4b; Figures S29–S32 and Table S5, Supporting Information). These results suggest that the h‐PVC film adsorbs PM species via reduction and chemical interactions, similar to the model h‐PVC polymer.

Unlike the h‐PVC polymer, the h‐PVC film maintains a macroscale structure with surface and internal pores without being dissolved in water owing to its incomplete hydrazination, as described above. Hence, the h‐PVC film can suffer from significant internal mass transfer resistance owing to its porous structure,^[^ ^37^ ^]^ resulting in its slower PM adsorption kinetics than that of the h‐PVC polymer. In, fact, the h‐PVC film progressively adsorbed PM ions until its *q*_e_ was reached at 12 h (*t*_eq_) (Figure 4c). The h‐PVC film exhibited a longer *t*_eq_ than the h‐PVC polymer (10–30 min) presumably because of its dense surface layer, which acts as a diffusion barrier layer. According to the Weber–Morris model, the h‐PVC film displayed three‐step adsorption kinetics (Figure 4c; Figures S33 and S34, Supporting Information). In the first step, PM ions slowly diffused for adsorption through the dense near‐surface region of the h‐PVC film, showing a moderate adsorption rate (*k*_d_). In the second step, PM ions rapidly diffused across the macroporous internal region of the h‐PVC film through its pores; among the adsorption steps, this step had the highest *k*_d_. Finally, in the third step, adsorption significantly slowed as the adsorption sites of the film became saturated,^[^ ^38^ ^]^ displaying the lowest *k*_d_.^[^ ^38^ ^]^ The adsorption rate‐determining step is defined as the step with the slowest adsorption rate except for the last adsorption equilibrium step.^[^ ^39^ ^]^ Hence, the first step (surface diffusion) is considered the adsorption rate‐determining step as the dense surface layer predominantly governs the overall PM adsorption rate by exerting significant internal mass transfer resistance. This stepwise PM adsorption mechanism was further confirmed by scanning electron microscopy–energy‐dispersive X‐ray spectroscopy (SEM–EDS) (Figure 4d; Figure S35, Supporting Information). In the first step, the adsorbed PM species were localized near the surface of the h‐PVC film. The PM species were then uniformly distributed throughout the h‐PVC film, with their density higher in the third step than in the second step.

Similar to the h‐PVC polymer, the h‐PVC film exhibited adsorption isotherms that fit the Langmuir isotherm model well (Figure 4e; Table S6, Supporting Information). The *q*_max_ values of the h‐PVC film were determined to be 355 (Au), 241 (Pt), and 141 (Pd) mg g^−1^. Compared with other reported macroscale PM adsorbents, the h‐PVC film exhibited comparable or even higher (for Pt) PM adsorption performance (Figure 4f; Table S7, Supporting Information) owing to its combined PM reduction and chemisorption mechanisms.

### 2.5 Practical Applications of the h‐PVC Plastic

**Figure 5:**
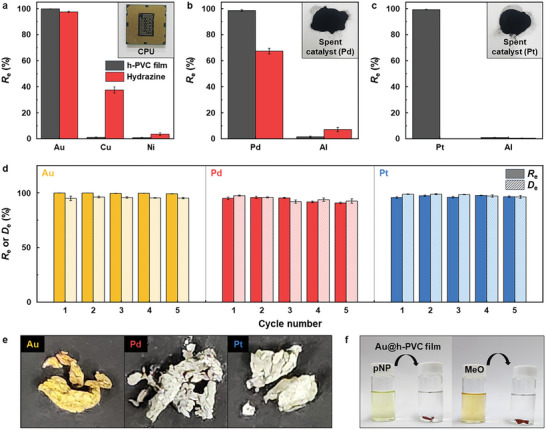
a–c) Recovery efficiency (*R*_e_) of the h‐PVC plastic film with real‐world leachates (h‐PVC film dose = 1.0 g L^−1^, pH = 2, contact time = 12 h): a) CPU and spent b) Pd and c) Pt catalyst leachates (insets: photographs of the real‐world samples). d) PM *R*_e_ and desorption efficiency (*D*_e_) of the h‐PVC film with real‐world leachates as a function of the adsorption–desorption cycle number (h‐PVC film dose = 1.0 g L^−1^, pH = 2, contact time = 12 h). e) Photographs of the PM particles obtained by calcinating the PM@h‐PVC films. f) Photographs illustrating the reduction of organic dyes (pNP and MeO) catalyzed by the Au@h‐PVC film. Data represents the mean ± standard deviation (*n* = 3).

The practical application of the h‐PVC plastic film as a PM adsorbent was verified via characterizations of its PM adsorption capacity and selectivity using real‐world leachates obtained from a central processing unit (CPU) (Au) and spent catalysts (Pt and Pd) (Figure S36, Supporting Information) in comparison with those of hydrazine (**Figure** **5**a–c). Although hydrazine exhibited a satisfactory *R*_e_ for Au (≈97%) and Pd (≈67%), it also showed a remarkable *R*_e_ for coexisting metal ions, thus exhibiting low PM selectivity owing to its low selective reduction mechanism.^[^ ^16^ ^]^ Moreover, hydrazine was ineffective at recovering Pt owing to its poor Pt reduction ability (Figure 5c).^[^ ^40^ ^]^ By contrast, the h‐PVC film displayed a considerably high *R*_e_ (>95%) for all PMs but a marginal *R*_e_ for coexisting metal ions; thus, its PM adsorption performance and selectivity were superior to those of hydrazine. Hydrazine, as a reducing agent, can reduce coexisting metal ions as well as PM ions regardless of their charge properties, leading to its low PM selectivity.^[^ ^16^ ^]^ The superior PM adsorption performance and selectivity of the h‐PVC film over hydrazine can be attributed to its dual action as an adsorbent and reductant, which induces combined chemisorption (electrostatic interaction and chelation) and reduction. Specifically, the high positive charge of the h‐PVC film induces strong electrostatic repulsion for coexisting metal cations (i.e., Cu^2+^, Ni^2+^, and Al^3+^)^[^ ^16^ ^]^ while exerting strong electrostatic attraction and chelation with anionic PM species, which are subsequently reduced by concentrated hydrazine groups (Figure S37, Supporting Information). The excellent PM selectivity of the h‐PVC film was further confirmed by its selective PM adsorption in mixed‐ion solution and simulated industrial wastewater (Figures S38 and S39, Supporting Information).

PM species were readily recovered from the resultant PM@h‐PVC films with a high *D*_e_ via treatment with desorption agents (HCl and thiourea), regenerating the h‐PVC film. The regenerated h‐PVC film maintained a high *R*_e_ and *D*_e_ (>90%) for all PMs over five adsorption–desorption cycles (Figure 5d), demonstrating its excellent recyclability. The PM@h‐PVC films were also refined into high‐purity (>98%) metallic PMs through air combustion (Figure 5e). Furthermore, because the PM@h‐PVC films contained PM NPs, they could be directly employed as catalysts for chemical reactions. The addition of the PM@h‐PVC films to organic dye (*para*‐nitrophenol (pNP) and methyl orange (MeO))‐containing aqueous solutions effectively catalyzed dye reduction, as verified by visual observations and UV–vis analysis (Figure 5f; Figures S40 and S41, Supporting Information).

Our proposed strategy was further extended to transform various forms of PVC waste plastics, including hose, wire sheath, and mold, into PM adsorbents (Figure S42, Supporting Information). All the resultant h‐PVC plastics displayed the characteristic hydrazine chemistry and asymmetric pore structure of the h‐PVC film (Figures S20, S43, and S44, Supporting Information). Although other h‐PVC forms exhibited a somewhat lower *R*_e_ for all PMs than the h‐PVC film, presumably owing to differences in their impurities/additives, they generally showed satisfactory *R*_e_ values, particularly for Au (Figure S45, Supporting Information). These results confirm the high versatility of our upcycling strategy.

### 2.6 TEA and LCA of Au Recovery Using the h‐PVC Plastic

The economic feasibility of the h‐PVC plastic film was evaluated for the industrial‐scale recovery of Au from the treatment of 100 tons of CPU waste daily. We developed two integrated Au recovery processes based on the experimental data: 1) a film calcination process that recovers Au via Au@h‐PVC film combustion and 2) a film regeneration process that regenerates the Au@h‐PVC film using desorption agents (thiourea and HCl) while recovering Au via electrowinning (Figure S46, Supporting Information).

**Figure 6:**
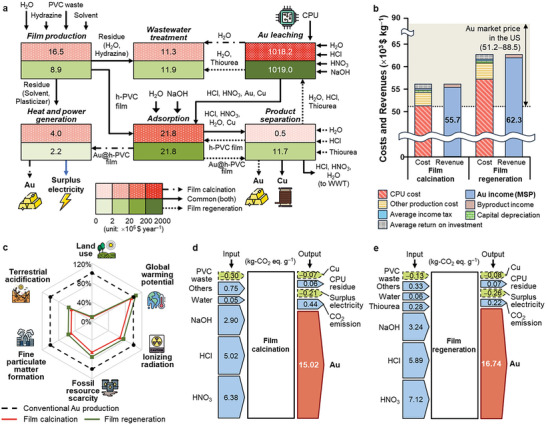
a) Construction of the subsystems and their contributions to the total cost of the complete processes. The processes also include storage (capital and operating costs are 0.5 × 10^6^ and 0.1 × 10^6^ $ year^−1^, respectively, for both film calcination and film regeneration) and utility (capital and operating costs are 0.8 × 10^6^ and 0.2 × 10^6^ $ year^−1^, respectively, for film calcination, while capital and operating costs are 0.6 × 10^6^ and 0.2 × 10^6^ $ year^−1^, respectively, for film regeneration) subsystems. b) Minimum selling price (MSP) of Au. c) Environmental impacts. Contributions of the process inputs and outputs to the global warming potential for the d) film calcination and e) film regeneration processes. Negative numbers are highlighted with dashed borders.

**Figure** **6**a and Tables S8 and S9 (Supporting Information) show the contributions of individual subsystems to the total cost of the complete process. In both processes, the Au leaching subsystem was the highest contributor to the total cost because of the high feedstock and reactor costs resulting from a 24 h residence time and acidic conditions. Compared with the film regeneration process, the film calcination process required a larger amount of the h‐PVC film make‐up because it does not include film regeneration, thus incurring additional film production costs associated with plasticizer removal and hydrazination. Moreover, the complete calcination of the h‐PVC film resulted in an increase in equipment (e.g., combustor and boiler) costs in the heat and power generation subsystem. On the other hand, the film calcination process required no cost for desorption and lower costs for electrowinning and wastewater treatment than the film regeneration process because it features smaller amounts of stillage and condensate. Furthermore, the film calcination process could raise additional revenue by selling surplus electricity, which is generated by film combustion, to the grid. The minimum selling price (MSP), which is the breakeven selling price, of Au for both processes was calculated using discounted cash flow analysis (Figure 6b).^[^ ^41^ ^]^ The feedstock price, electrowinning performance, and solvent loading had a significant effect on the MSP (Figure S47, Supporting Information). Compared with the film regeneration process, the film calcination process led to a lower MSP (55.7 × 10^3^ vs 62.3 × 10^3^ $ kg^−1^) because of two reasons. First, its Au production rate (19.2 tons year^−1^) is higher than that of the film regeneration process (17.2 tons year^−1^) relying on desorption followed by electrowinning. Second, its higher credits (831.3 vs 257.0 kW), which are gained by selling surplus electricity, outweigh the capital and operating costs raised by the film makeup. Importantly, considering that their MSPs were lower than the Au market price in the last five years (51.2 × 10^3^–88.5 × 10^3^ $ kg^−1^), both processes were cost‐competitive.

The environmental benefits of the proposed processes were also assessed (Figure 6c; Table S10, Supporting Information). In terms of land use, terrestrial acidification, fine particulate matter formation, fossil resource scarcity, and ionizing radiation, both processes were environmentally advantageous over the conventional Au production process (i.e., Au mining including beneficiation, concentration, and refinement). Both the film calcination and generation processes also showed significantly lower ecotoxicity and human toxicity than the conventional Au production process (Table S10, Supporting Information). Notably, the film calcination process had a lower global warming potential (GWP, 15.0 kg‐CO_2_ eq. g^−1^) than the film regeneration process (16.7 kg‐CO_2_ eq. g^−1^) because the former does not employ a desorption subsystem requiring the use of thiourea, thereby reducing indirect CO_2_ emissions. Compared with the conventional Au production process, the film calcination process to treat 100 tons of CPU waste can reduce CO_2_ emissions by 16 017 tons per year. Finally, the contributions of the process inputs and outputs to the GWP were analyzed (Figure 6d,e). The environmental credits obtained by utilizing CPU and PVC wastes and producing surplus electricity led to negative CO_2_ emissions. By contrast, most chemical agents, including NaOH, HCl, nitric acid (HNO_3_), and thiourea, had significant adverse effects on the GWP. This finding suggests that our proposed Au recovery process can be further improved by replacing the chemical agents with those with a lower GWP and optimizing the operating conditions.

We further compared the TEA and LCA results of our h‐PVC film processes with those of the chemical reduction process utilizing the hydrazine‐reducing agent (Figure S48, Supporting Information). The chemical reduction process showed a relatively lower total cost than the h‐PVC film processes owing to its simplicity resulting from the absence of the film fabrication process and the use of the simple filtration process to recover Au (Tables S8 and S9, Supporting Information). Nevertheless, compared with the film calcination process, the chemical reduction process led to a higher MSP (Table S10, Supporting Information) mainly owing to its lower Au recovery and production rates. Furthermore, the environmental impacts of the chemical reduction process were comparable to those of the film calcination and regeneration processes across most categories (Table S11, Supporting Information). However, it should be noted that the purity of Au recovered via the chemical reduction process (77%) is remarkably lower than that obtained via both film processes (98%) (Table S10, Supporting Information) because of the considerably lower Au selectivity of hydrazine, demonstrated above. Hence, the chemical reduction process requires additional refinement processes to improve the purity of Au, which would considerably increase the MSP and adverse environmental impacts, making it economically and environmentally disadvantageous over the film processes.

## 3 Conclusion

In this study, we successfully demonstrated the upcycling of PVC waste plastics into high‐performance PM adsorbents. A PVC waste plastic film was transformed into a porous h‐PVC film via benign solvent treatment and subsequent hydrazination. The h‐PVC film exhibited higher PM adsorption performance (particularly for Pt) than many other macroscale PM adsorbents. Compared with hydrazine, the h‐PVC film displayed considerably higher PM adsorption capacity and selectivity with real‐world leachates and enabled easier collection. The excellent PM recovery performance of the h‐PVC film was ascribed to its high reduction ability and chemical (electrostatic and chelation) interactions, which were imparted by its high‐density hydrazine groups. The PM‐adsorbed h‐PVC film could be regenerated, calcined into high‐purity PMs, or directly employed as a catalyst in the dye reduction reaction. Our proposed strategy was successfully extended to the upcycling of various PVC waste plastic forms into PM adsorbents. The practical feasibility of recovering Au from CPU waste using the h‐PVC plastic film was analyzed via TEA and LCA. Although both proposed processes were cost‐competitive and had positive environmental impacts, the film calcination process was more cost‐effective (i.e., lower MSP) and environmentally beneficial (i.e., lower GWP) than the film regeneration process. The upcycling of waste plastics into value‐added PM adsorbents via minimal and environmentally benign treatments can enable the sustainable use of carbon and PM resources with positive environmental impacts. Our study also provides a material platform for designing high‐performance adsorbents for recovering other valuable resources or removing hazardous matter in various industries, including electronics, batteries, electrochemical devices, and wastewater treatment.

## 4 Experimental Section

### Synthesis of the h‐PVC Polymer

PVC polymer (5.0 g) was dissolved in *N*‐methylpyrrolidone (154.0 mL) at 80 °C. Hydrazine hydrate (25.6 g) was injected dropwise into the PVC polymer solution, and the mixture was allowed to react at 80 °C for 5 d, which resulted in the complete hydrazination of the PVC polymer (Figure S1, Supporting Information). The reaction temperature (80 °C) was selected based on previous reports on the amine‐functionalization reaction of PVC^[^ ^42^ ^]^ and preliminary experiments (Figure S49, Supporting Information). Thereafter, the mixture was cooled to 25 °C and then added dropwise to acetone to precipitate the h‐PVC polymer. The obtained h‐PVC polymer was vacuum‐dried for 1 d.

### Fabrication of the h‐PVC Plastic

A commercially available PVC plastic film (10 × 10 cm^2^, 1.2 g) or other forms of PVC plastic (1.2 g) were soaked in DMSO (45 mL) for 30 min and then immersed in EtOH (225 mL) for 1 h. The obtained d‐PVC plastic was subsequently immersed in a hydrazine hydrate (4.6 g) solution in DMSO (30.7 mL) and reacted at 80 °C for 24 h, which resulted in the saturated *q*_e_ of the resultant h‐PVC film (Figure S23, Supporting Information). The solution was cooled to 25 °C, and the obtained h‐PVC plastic was washed with deionized (DI) water.

### PM Adsorption Tests Using Model PM Aqueous Solutions

Each PM (1000 mg L^−1^) standard solution was diluted using DI water to prepare aqueous solutions with various PM concentrations. The solution pH was adjusted using HCl (1 N) and sodium hydroxide (NaOH, 1 N). A predetermined weight (*w*) of the adsorbent (h‐PVC polymer (10 mg) or plastic (50 mg)) was introduced to each PM aqueous solution (*V*: 50 mL) and then shaken for 3 h (h‐PVC polymer) or 12 h (h‐PVC plastic). Subsequently, the supernatant was collected by either permeating the mixture containing PM@h‐PVC polymer precipitates through a polysulfone ultrafiltration (PSU) membrane via dead‐end filtration (HP4750 stirred cell, Sterlitech) at 5 bar or removing the PM@h‐PVC plastic from the mixture using tweezers. The PM concentrations of the PM aqueous solutions before (*C*_i_) and after (*C*_e_) the introduction of the adsorbent were quantified via inductively coupled plasma–mass spectrometry (ICP–MS, NexION 300D, PerkinElmer) to determine *q*_e_ (mg g^−1^) as follows:

(1) $$q_{e}=\frac{\left(C_{i}-C_{e}\right)\times V}{w}$$

*R*_e_ (%) was determined as follows:

(2) $$R_{e}=\frac{C_{i}-C_{e}}{C_{i}}\times \;100$$

At least three replicates were tested to average the results.

### PM Recovery Tests Using Real‐world Leachates

A spent computer CPU was used as a real‐world Au source, while spent catalysts were employed as real‐world Pd and Pt sources. Real‐world leachates were prepared by following the protocol reported in a previous study.^[^ ^16^ ^]^ Briefly, each real‐world PM source (CPU (20 g) or spent catalyst (2 g)) was soaked in aqua regia (50 mL), which comprises HCl and HNO_3_ at a volume ratio of 3:1, for 1 d. Thereafter, the solution was permeated through a cellulose fiber membrane filter, and the supernatant was diluted to 1 L using DI water. The pH of the resultant solution was adjusted to 2 using NaOH (1 N). The h‐PVC plastic film or hydrazine (50 mg) was introduced to each of the obtained real‐world leachates (50 mL) and then shaken for 12 h. Subsequently, the supernatant was collected by either removing the h‐PVC film from the mixture using tweezers or permeating the hydrazine‐containing mixture through a PSU membrane via dead‐end filtration at 5 bar. The metal concentrations of the real‐world leachates before (*C*_s_) and after the introduction of the h‐PVC film or hydrazine were quantified via ICP–MS to determine *R*_e_. The metal composition and *C*_s_ of the real‐world leachates were summarized in Figure S36 (Supporting Information).

### Regeneration of the h‐PVC Plastic Film

The PM@h‐PVC plastic film collected after the PM recovery tests using real‐world leachates was immersed in an aqueous solution of the desorption reagents (HCl (1 N) and thiourea (1 N)) and ultrasonicated for 30 min to induce the desorption of PM species from the film. The supernatant was collected by removing the film from the mixture using tweezers, and its PM concentration (*C*_d_) was quantified via ICP–MS to determine *D*_e_ (%) as follows:

(3) $$D_{e}=\frac{C_{d}}{C_{s}\times R_{e}}\times \;100$$

The collected h‐PVC film was thoroughly rinsed with water to ensure the complete removal of residual thiourea and ionic species and freeze‐dried for 1 d. The resultant h‐PVC film was reused for the above adsorption–desorption process five times.

### Calcination of the PM@h‐PVC Plastic Film

The PM@h‐PVC plastic film collected after the PM recovery tests using real‐world leachates was vacuum‐dried for 1 d, placed in a ceramic pan, and directly subjected to a butane flame (1300 °C) using a portable torch for 5 min for complete combustion. The obtained calcined particles were digested in the predetermined volume (*V*_AR_) of aqua regia, and their concentration (*C*_PM_) was quantified via ICP–MS to determine their purity as follows:

(4) $$\mathrm{Purity}\;\left(\%\right)=\frac{w_{\mathrm{CP}}}{V_{\mathrm{AR}}\times C_{\mathrm{PM}}}\times \;100$$

where *w*_CP_ was the weight of the calcined particles.

### Dye Reduction Activity of the PM@h‐PVC Plastic Film

The PM@h‐PVC plastic film collected after the PM recovery tests using real‐world leachates was washed with DI water, vacuum‐dried for 1 d, and introduced to a dye (pNP or MeO, 0.01 mm)/NaBH_4_ (1 mm) aqueous solution (10 mL). The progress of the dye reduction reaction was monitored by visual observation and UV–vis analysis.

### TEA Analysis

The process models were developed using Aspen Plus V14.3 software. The size of the required equipment was determined based on the material balance calculated for processing 100 tons of CPU waste daily. The capital costs for equipment such as the pump, vessel, and distillation column were calculated using Aspen Process Economic Analyzer V14.3.^[^ ^43^ ^]^ The capital costs for the remaining equipment, such as the adsorption bed, combustor, and wastewater treatment system, were determined using the following scaling formula:^[^ ^41^, ^44^ ^]^

(5) $$\mathrm{New}\;\mathrm{cost}=\mathrm{Base}\;\mathrm{cost}\times {\left(\frac{\mathrm{New}\;\mathrm{size}}{\mathrm{Base}\;\mathrm{size}}\right)}^{n}$$

where the base cost and corresponding base size were obtained from the literature. The exponent *n* represents the economy of scale, with values between 0 and 1. All capital and operating costs were estimated using a common basis year of 2023. The detailed economic parameters and assumptions were provided in Table S12 (Supporting Information). Heat integration using pinch analysis was also conducted to reduce utility consumption in the processes (Table S13, Supporting Information).

### LCA Analysis

LCA was conducted following International Standard Organization guidelines (ISO 14040 and 14044).^[^ ^45^, ^46^ ^]^ The analysis consists of four main steps: definition of the goal and scope, life‐cycle inventory analysis, life‐cycle impact assessment, and interpretation of life‐cycle results. The system boundary was set to be the integrated process, and the functional unit was set to 1 kg of Au. The environmental impacts were evaluated using SimaPro 9.1 with the Ecoinvent database 3.6 and the ReCiPe 2016 midpoint approach.^[^ ^47^ ^]^

### Statistical Analysis

The experimental data with error bars was presented as the mean ± standard deviation. Sample size (*n*) for each statistical analysis was indicated in the figure legends. All statistical analyses were performed using Microsoft Excel software.

## Conflict of Interest

The authors declare no conflict of interest.

## Supporting information



Supporting Information

## Data Availability

The data that support the findings of this study are available from the corresponding author upon reasonable request.

## References

[1] Y. Chen , M. Xu , J. Wen , Y. Wan , Q. Zhao , X. Cao , Y. Ding , Z. L. Wang , H. Li , Z. Bian , Nat. Sustain. 2021, 4, 618.

[2] A. T. Nakhjiri , H. Sanaeepur , A. E. Amooghin , M. M. A. Shirazi , Desalination 2022, 527, 115510.

[3] Y. Chen , Q. Qiao , J. Cao , H. Li , Z. Bian , Joule 2021, 5, 3097.

[4] Y. Ding , S. Zhang , B. Liu , H. Zheng , C. Chang , C. Ekberg , Resour. Conserv. Recycl. 2019, 141, 284.

[5] F. Yang , Z. Yan , J. Zhao , S. Miao , D. Wang , P. Yang , J. Mater. Chem. A. 2020, 8, 3438.

[6] M. D. Rao , K. K. Singh , C. A. Morrison , J. B. Love , Sep. Purif. Technol. 2021, 263, 118400.

[7] J. Wang , B. Zeng , J. Lv , Y. Lu , H. Chen , ACS Sustainable Chem. Eng. 2020, 8, 16952.

[8] L. M. M. Kinsman , B. T. Ngwenya , C. A. Morrison , J. B. Love , Nat. Commun. 2021, 12, 6258. 34716348 10.1038/s41467-021-26563-7 PMC8556376

[9] Y. Chen , F. Zi , X. Hu , P. Yang , Y. Ma , H. Cheng , Q. Wang , X. Qin , Y. Liu , S. Chen , C. Wang , Sep. Purif. Technol. 2020, 230, 115834.

[10] Z. Chang , F. Li , X. Qi , B. Jiang , J. Kou , C. Sun , J. Hazard. Mater. 2020, 391, 122175. 32045802 10.1016/j.jhazmat.2020.122175

[11] R. Fan , H. Min , X. Hong , Q. Yi , W. Liu , Q. Zhang , Z. Luo , J. Hazard. Mater. 2019, 364, 780. 30447562 10.1016/j.jhazmat.2018.05.061

[12] J. Guo , Y. Wu , Z. Wang , J. Yu , J.‐R. Li , J. Mater. Sci. 2022, 57, 10886.

[13] X. Zhang , H. Li , M. Ye , H. Zhang , G. Wang , Y. Zhang , Sep. Purif. Technol. 2022, 292, 121021.

[14] J. Guo , X. Fan , Y. Li , S. Yu , Y. Zhang , L. Wang , X. Ren , J. Hazard. Mater. 2021, 415, 125617. 33743379 10.1016/j.jhazmat.2021.125617

[15] A. Butewicz , K. C. Gavilan , A. V. Pestov , Y. Yatluk , A. W. Trochimczuk , E. Guibal , J. Appl. Polym. Sci. 2010, 116, 3318.

[16] S. S. Shin , Y. Jung , S. Jeon , S.‐J. Park , S.‐J. Yoon , K. W. Jung , J. W. Choi , J. H. Lee , Nat. Commun. 2024, 15, 3889. 38719796 10.1038/s41467-024-48090-x PMC11079046

[17] L. B. Hamdy , A. Gougsa , W. Y. Chow , J. E. Russell , E. García‐Díez , V. Kulakova , S. Garcia , A. R. Barron , M. Taddei , E. Andreoli , Mater. Adv. 2022, 3, 3174.

[18] S. Rawat , N. Misra , M. Singh , M. Tiwari , A. Ghosh , S. A. Shelkar , S. Samanta , N. K. Goel , V. Kumar , J. Water Proc. Eng. 2024, 60, 105109.

[19] F. R. Xiu , X. Tan , Y. Qi , M. Wang , J. Hazard. Mater. 2023, 441, 129820. 36103762 10.1016/j.jhazmat.2022.129820

[20] Y. Zhang , H. Dong , W. Du , C. Dong , M. Xiong , Z. Yang , S. Zhao , H. He , Z. Nie , Chem. Eng. J. 2025, 505, 159419.

[21] P. Zhu , R. Wang , React. Funct. Polym. 2024, 199, 105902.

[22] J. K. Bediako , S. W. Park , J. W. Choi , M. H. Song , Y. S. Yun , J. Environ. Chem. Eng. 2019, 7, 102839.

[23] L. Zhang , X. Zha , G. Zhang , J. Gu , W. Zhang , Y. Huang , J. Zhang , T. Chen , J. Mater. Chem. A 2018, 6, 10217.

[24] R. P. Rietra , T. Hiemstra , W. H. van Riemsdijk , Environ. Sci. Technol. 2001, 35, 3369. 11529579 10.1021/es000210b

[25] H. Chaudhuri , X. Lin , Y. S. Yun , J. Hazard. Mater. 2023, 451, 131206. 36931220 10.1016/j.jhazmat.2023.131206

[26] C. Nan , J. Dong , H. Tian , H. Shi , S. Shen , J. Xu , X. Li , T. Shi , J. Mol. Liq. 2018, 256, 489.

[27] S. Lin , D. H. K. Reddy , J. K. Bediako , M. H. Song , W. Wei , J. A. Kim , Y. S. Yun , J. Mater. Chem. A 2017, 5, 13557.

[28] M. Rezaee , H. Abdollahi , R. Saneie , A. Mohammadzadeh , A. Rezaei , M. H. K. Darvanjooghi , S. K. Brar , S. Magdouli , Chemosphere 2022, 298, 134283. 35288186 10.1016/j.chemosphere.2022.134283

[29] J. Wang , X. Guo , J. Hazard. Mater. 2020, 390, 122156. 32006847 10.1016/j.jhazmat.2020.122156

[30] M. A. Al‐Ghouti , D. A. Da'ana , J. Hazard. Mater. 2020, 393, 122383. 32369889 10.1016/j.jhazmat.2020.122383

[31] Z. Qin , H. Ding , R. Huang , S. Tong , Chem. Eng. J. 2022, 428, 132493.

[32] G. Grause , S. Hirahashi , H. Toyoda , T. Kameda , T. Yoshioka , J. Mater. Cycles Waste Manage. 2017, 19, 612.

[33] D. Y. Kim , M. Kim , S. Jeon , J. Lee , H. Park , Y. I. Park , S. J. Park , J. H. Lee , J. Membr. Sci. 2023, 688, 122114.

[34] H. L. Qian , W. P. Huang , Y. Fang , L. Y. Zou , W. J. Yu , J. Wang , K. F. Ren , Z. K. Xu , J. Ji , ACS Appl. Mater. Interfaces 2021, 13, 57000. 34816710 10.1021/acsami.1c18333

[35] R. Zangi , J. B. Engberts , J. Am. Chem. Soc. 2005, 127, 2272. 15713106 10.1021/ja044426f

[36] Y. Chen , S. Lai , S. Jiang , Y. Liu , C. Fu , A. Li , Y. Chen , X. Lai , J. Hu , Mater. Lett. 2015, 157, 15.

[37] J. Kärger , R. Valiullin , Chem. Soc. Rev. 2013, 42, 4172. 23377106 10.1039/c3cs35326e

[38] S.‐H. Park , S. S. Shin , C. H. Park , S. Jeon , J. Gwon , S. Y. Lee , S. J. Kim , H. J. Kim , J. H. Lee , J. Hazard. Mater. 2020, 394, 122512. 32200239 10.1016/j.jhazmat.2020.122512

[39] E. Deze , S. K. Papageorgiou , E. P. Favvas , F. K. Katsaros , Chem. Eng. J. 2012, 209, 537.

[40] H. Wu , Y. Wang , L. O. Jones , W. Liu , L. Zhang , B. Song , X. Y. Chen , C. L. Stern , G. C. Schatz , J. F. Stoddart , Angew. Chem. Inter. Ed. 2021, 60, 17587. 10.1002/anie.202104646 34031957

[41] B. Klein , I. McNamara , R. Davis , A. Mittal , D. Johnson , D. (No. NREL/TP‐5100‐80652). National Renewable Energy Lab. (NREL) 2021, Golden, CO (USA).

[42] G. Sneddon , J. C. McGlynn , M. S. Neumann , H. M. Aydin , H. H. P. Yiu , A. Y. Ganin , J. Mater. Chem. A 2017, 5, 11864.

[43] C. Gong , H. Kim , I. Ro , Y. J. Kim , W. Won , Energy Convers. Manage. 2024, 316, 118831.

[44] B. Ahn , H. Sohn , J. J. Liu , W. Won , ACS Sustainable Chem. Eng. 2024, 12, 8630.

[45] I. O. Standardization,. ISO/IEC 25010‐Systems and software engineering‐Systems and software Quality Requirements and Evaluation (SQuaRE)‐System and software quality models 2011.

[46] ISO 14044: Environmental management‐Life cycle assessment‐Requirements and guidelines 2006.

[47] D. Park , H. Lee , W. Won , Chem. Eng. J. 2024, 487, 150540.

