# Supplementary material for: Ecofriendly Upcycling of Poly(vinyl chloride) Waste Plastics into Precious Metal Adsorbents
Source: Adv Sci (Weinh). 2025 May 9;12(28):2503157. doi: 10.1002/advs.202503157 (PMC12302614; doi:10.1002/advs.202503157)
Supplement: Supplementary file 1 — Supporting Information [file ADVS-12-2503157-s001.docx]

Supporting Information

Ecofriendly Upcycling of Poly(vinyl chloride) Waste Plastics into Precious Metal Adsorbents

Seung Su Shin, Seungho Lee, Sung-Joon Park, Hansoo Kim, Juyeon Choi, Wangyun Won^*^, and Jung-Hyun Lee^*^

**Methods**

*Materials*: PVC polymer (*M*_w_: 233 kg mol^−1^), hydrazine hydrate (50−60%), HNO_3_ (70%), HCl (37%), thiourea (≥99.0%), and Au, Pd, and Pt (1000 mg L^−1^) standard solutions were purchased from Sigma-Aldrich. *N*-Methylpyrrolidone (99.5%), DMSO (99.5%), DMSO-d_6_ (99.9 atom% D), acetone (99.8%), methanol (99.9%), EtOH (99.9%), isopropyl alcohol (99.9%), HCl (1 N) and NaOH (1 N) standard solutions, and NaBH_4_ (98%) were obtained from Daejung Chemical. Sodium nitrate (NaNO_3_, 99%), magnesium nitrate hexahydrate (Mg(NO_3_)_2_⋅6H_2_O, 99%), aluminum nitrate nonahydrate (Al(NO_3_)_3_⋅9H_2_O, 98%), iron nitrate nonahydrate (Fe(NO_3_)_3_⋅9H_2_O, 98%), cobalt nitrate hexahydrate (Co(NO_3_)_2_⋅6H_2_O, 98%), nickel nitrate hexahydrate (Ni(NO_3_)_2_⋅6H_2_O, 97%), copper nitrate trihydrate (Cu(NO_3_)_2_⋅3H_2_O, 99%), zinc nitrate hexahydrate (Zn(NO_3_)_2_⋅6H_2_O, 98%), cadmium nitrate tetrahydrate (Cd(NO_3_)_2_⋅4H_2_O, 98%), and lead nitrate (Pb(NO_3_)_2_, 99%) were purchased from Sigma-Aldrich. MeO and pNP (99%) were acquired from Alfa Aesar. DI water was prepared using a Milli-Q purification system (Millipore). PVC plastic film (thickness: 200 μm), hose, wire sheath, and mold were purchased from Filmbank, Hankil Co., Misumi, and Daehan Mold, respectively. PSU membranes (M-M2540PS20, molecular weight cut-off: 20 kg mol^−1^) and cellulose fiber membrane filters (JIS P 3801, pore size: 1 μm) were obtained from Applied Membranes Inc. and Advantec, respectively.

*Material Characterization*: The chemical structures of the polymers and plastics were identified via Fourier transform infrared spectroscopy (FT-IR, Spectrum Two spectrometer, PerkinElmer). FT-IR spectra were collected in the wavelength range of 800 to 4000 cm^−1^ with an attenuated total reflectance unit. XPS (Nexsa, Thermo Fisher) was performed to characterize the chemical compositions and structures of the polymers and plastic films before and after PM adsorption. XPS spectra were obtained using monochromatized Al-Kα radiation at 1.49 keV. A zeta potential analyzer (ELSZ-2000ZS, Otsuka) was used to measure the surface zeta potentials of the polymers and plastic films. XRD (Smart Lab, Rigaku) was performed to characterize the crystal structures of the polymers and plastic films before and after PM adsorption. Diffraction patterns were obtained using Cu K*α* as a radiation source (*λ* = 0.154 nm) in the angle range of 10° ≤ 2*θ* ≤ 90° with a scan rate of 0.5° min^−1^.The surface and cross-sectional morphologies of the PVC, d-PVC, and h-PVC plastics were examined using SEM (SU5000, Hitachi). The cross-sectional sample was prepared by immersing the plastic in liquid nitrogen for 1 min and fracturing the frozen sample with a blade. The prepared surface and cross-sectional samples were coated with Pt using an ion sputter at 15 mA for 60 s and imaged at an accelerating voltage of 5 kV. The EDS instrument equipped in the SEM was employed to identify the distribution of PM species in the PM@h-PVC plastic films. All SEM−EDS images were obtained at an accelerating voltage of 15 kV. The morphologies of the PM@h-PVC polymer precipitates and plastic films were analyzed using FE-TEM (Tecnai G2 F30ST, FEI). The PM@h-PVC precipitate sample (5 mg) was dispersed in DI water (10 mL), and the resultant solution was dropped onto a copper grid, followed by drying at room temperature. The TEM images of the PM@h-PVC precipitates were obtained at an accelerating voltage of 200 kV. The EDS instrument equipped in the TEM was employed to confirm the presence of PM species in the PM@h-PVC plastic films. The film sample was embedded in EPON™ resin (Polybed 812), followed by curing at 60 °C for 24 h. Approximately 100 nm-thick slices were cut on an ultramicrotome (Reichert Ultracut S, Leica Microsystems) and mounted onto a copper grid. The TEM and EDS images of the PM@h-PVC plastic films were obtained at an accelerating voltage of 200 kV. The overall porosity of the d-PVC and h-PVC plastic films was determined using a gravimetric method. The film was freeze-dried, weighed (*m*_d_), and then soaked in isopropyl alcohol for 12 h. Thereafter, the residual isopropyl alcohol was removed with tissue. The thickness (*t*_f_), area (*A*), and weight (*m*_w_) of the wet film were measured to calculate its overall porosity (*ε*) as follows:

$\text{ε}\text{ }\text{}\text{ }\frac{\text{(}\text{m}_{\text{w}}\text{ }\text{}\text{ }\text{m}_{\text{d}}\text{)}}{\text{(}\text{d}\text{ }\text{}\text{A}\text{ }\text{}\text{t}_{\text{f}}\text{)}}\text{ }\text{}\text{ 100}$ (S1)

where *d* is the density of isopropyl alcohol (0.79 g cm^−3^).

The chemical structure of the plasticizer present in the PVC plastic film was identified via proton nuclear magnetic resonance spectroscopy (^1^H NMR, JNM-ECZ500R, JEOL). A UV−vis spectrometer (Cary 5000, Agilent Technologies) was used to confirm the formation of Au NPs in the Au aqueous solution after the introduction of the h-PVC polymer.

*PM Adsorption Kinetics of the h-PVC Polymer and Plastic Film*: The h-PVC polymer (10 mg) was introduced to the PM (200 mg L^−1^) aqueous solution (50 mL; pH = 2) and then shaken for 1−180 min. After a predetermined contact time (*t*), the supernatant was collected by permeating the mixture containing PM@h-PVC precipitates through a PSU membrane via dead-end filtration at 5 bar, and its PM ion concentration was measured via ICP−MS to determine the PM adsorption capacity (*q*_t_) of the h-PVC polymer. The data of *q*_t_ versus *t* were fitted to the pseudo-first-order and pseudo-second-order models.^[1]^ The pseudo-first-order model assumes that adsorption occurs via physisorption on a few adsorption sites, and its rate is predominantly affected by the diffusion rate of the adsorbate, as described by:

$\text{q}_{\text{t}}\text{ }\text{}\text{ }\text{q}_{\text{e}}\left( \text{1 }\text{}\text{ }\text{e}^{\text{}\text{k}_{\text{1}}\text{t}} \right)$ (S2)

where *q*_e_ is the equilibrium PM adsorption capacity and *k*_1_ is the rate constant.

The pseudo-second-order model assumes that adsorption occurs via chemisorption on abundant adsorption sites, as described by:

$\text{q}_{\text{t}}\text{ }\text{}\text{ }\frac{\text{k}_{\text{2}}\text{q}_{\text{e}}^{\text{2}}\text{t}}{\text{1}{\text{ }\text{}\text{ }\text{k}}_{\text{2}}\text{q}_{\text{e}}\text{t}}$ (S3)

where *k*_2_ is the rate constant.

Similarly, the h-PVC plastic film (50 mg) was introduced to the PM (200 mg L^−1^) aqueous solution (50 mL; pH = 2) and then shaken for 10−1440 min. After a certain *t*, the supernatant was collected by removing the PM@h-PVC film from the mixture using tweezers, and its PM ion concentration was measured via ICP−MS to determine *q*_t_. The data of *q*_t_ versus *t*^0.5^ were fitted to the Weber−Morris model,^[1]^ which assumes that the internal diffusion of the adsorbate dominates over its adsorption, as described by:

$\text{q}_{\text{t}}{\text{ }\text{}\text{ }\text{k}}_{\text{d}}\text{t}^{\text{0.5}} \text{+} \text{C}$ (S4)

where *k*_d_ is the rate constant and *C* is a constant related to the thickness of the boundary layer.

*PM Adsorption Isotherms of the h-PVC Polymer and Plastic Film*: A predetermined amount of the adsorbent (h-PVC polymer (10 mg) or plastic film (50 mg)) was introduced to PM aqueous solutions (50 mL; pH = 2) with different initial PM ion concentrations (*C*_i_) and then shaken for 3 h (h-PVC polymer) and 24 h (h-PVC plastic film). Subsequently, the supernatant was collected by either permeating the mixture containing PM@h-PVC polymer precipitates through a PSU membrane via dead-end filtration at 5 bar or removing the PM@h-PVC film from the mixture using tweezers. The PM ion concentration of the supernatant solution (*C*_e_) was measured via ICP−MS to determine *q*_e_. The data of *q*_e_ versus *C*_e_ were fitted to the Langmuir and Freundlich models.^[2]^ The Langmuir model assumes that each active site can adsorb only a single adsorbate in a monolayer, as described by:

$\text{q}_{\text{e}}\text{ }\text{}\text{ }\frac{\text{q}_{\text{m}\text{ax}}\text{K}_{\text{L}}\text{C}_{\text{e}}}{\text{1 }\text{}\text{ }\text{K}_{\text{L}}\text{C}_{\text{e}}}$ (S5)

where *q*_max_ is the maximum PM adsorption capacity of the adsorbent and *K*_L_ is the Langmuir constant.

The Freundlich equation is an empirical model that assumes that each active site can adsorb multiple adsorbates by forming multilayers, as described by:

$\text{q}_{\text{e}}{\text{ }\text{}\text{ }\text{K}}_{\text{F}}\text{C}_{\text{e}}^{\text{1/}\text{n}}$ (S6)

where *K*_F_ is the Freundlich constant and *n* is a constant related to the adsorption capacity and favorability of the adsorbent.

*Regeneration of the h-PVC Polymer*: The h-PVC polymer (10 mg) was introduced to the PM aqueous solution (pH = 2) at a low *C*_i_ of 10 mg L^−1^, which corresponds to the typical PM ion concentration of real-world leachates,^[3]^ and then shaken for 3 h. The supernatant was collected by permeating the mixture containing PM@h-PVC precipitates through a PSU membrane via dead-end filtration at 5 bar, and its PM ion concentration was measured via ICP−MS to determine *R*_e_. The collected PM@h-PVC precipitates were immersed in an aqueous solution of the desorption reagents (thiourea (1 N) and HCl (1 N)) and ultrasonicated for 30 min to induce the desorption of PM species from the precipitates. The mixture was permeated through a PSU membrane via dead-end filtration at 5 bar. Small thiourea molecules (molecular weight: 76.12 g mol^−1^) and ionic species (HCl and PM ions) permeated through the membrane, whereas the large h-PVC polymer (molecular weight: 217 kg mol^−1^) was screened, enabling the complete collection of the h-PVC polymer. The PM ion concentration of the permeate solution (*C*_d_) was measured via ICP−MS to calculate *D*_e_ as follows:

$\text{D}_{\text{e}}\text{ }\text{}\text{ }\frac{\text{C}_{\text{d}}}{\text{C}_{\text{i}}\text{ }\text{}\text{ }\text{R}_{\text{e}}}\text{ }\text{}\text{ 100}$ (S7)

The collected h-PVC polymer was immersed in methanol while stirring for 1 h and then filtered through a cellulose membrane filter to completely remove loosely bound thiourea and ionic species. Methanol was used as a washing solvent because the h-PVC polymer is marginally soluble in methanol, forming readily collectible large clusters, whereas thiourea and ionic species are highly soluble in methanol. The collected h-PVC polymer was freeze-dried and then reused for the above adsorption−desorption process five times.

*PM Selectivity Tests of the h-PVC Film*: The single metal (PM, Cu, Ni, and Al, 100 mg L^−1^) aqueous solution was prepared by diluting each PM (1000 mg L^−1^) standard solution using DI water or dissolving copper nitrate trihydrate, nickel nitrate hexahydrate, or aluminum nitrate nonahydrate in DI water. The mixed-ion solution containing PM, Fe, and Cu (100 mg L^−1^ each) ions was also prepared using the respective PM (1000 mg L^−1^) standard solution, iron nitrate nonahydrate, and copper nitrate trihydrate. Additionally, the simulated industrial wastewater containing PM, Na, Mg, Al, Fe, Co, Ni, Cu, Zn, Cd, and Pb (100 mg L^−1^ each) ions was prepared using the respective PM (1000 mg L^−1^) standard solution, sodium nitrate, magnesium nitrate hexahydrate, aluminum nitrate nonahydrate, iron nitrate nonahydrate, cobalt nitrate hexahydrate, nickel nitrate hexahydrate, copper nitrate trihydrate, zinc nitrate hexahydrate, cadmium nitrate tetrahydrate, and lead nitrate. The solution pH was adjusted to 2. The h-PVC film (50 mg) was introduced to each prepared solution (50 mL) and then shaken for 12 h. Subsequently, the supernatant was collected by removing the h-PVC film from the solution using tweezers. The metal ion concentrations of the solutions before and after the introduction of the h-PVC film were quantified via ICP−MS to determine recovery efficiency (*R*_e_, %).

**Note S1. Redox Mechanisms between h-PVC and PM Ions**

The redox reaction between the protonated hydrazine (−NHNH_3_^+^) groups of h-PVC and Au ions (AuCl_4_^−^) can be described as follows^[4]^:

(redox reaction) 3(−NHNH_3_^+^) + 2AuCl_4_^−^ → 2Au + 3(−N=NH) + 9H^+^ + 8Cl^−^ (S8)

(oxidation half-reaction) −NHNH_3_^+^ → −N=NH + 3H^+^ + 2e^−^ (S9)

(reduction half-reaction) AuCl_4_^−^ + 3e^−^ → Au + 4Cl^−^ (S10)

The redox reaction between the −NHNH_3_^+^ groups of h-PVC and Pd ions (PdCl_4_^2−^) can also be described as follows^[4]^:

(redox reaction) −NHNH_3_^+^ + PdCl_4_^2−^ → Pd + −N=NH + 3H^+^ + 4Cl^−^ (S11)

(oxidation half-reaction) −NHNH_3_^+^ → −N=NH + 3H^+^ + 2e^−^ (S12)

(reduction half-reaction) PdCl_4_^2−^ + 2e^−^ → Pd + 4Cl^−^ (S13)

The redox reaction between the −NHNH_3_^+^ groups of h-PVC and Pt ions (PtCl_6_^2−^) can be described as follows^[4]^:

(redox reaction) 2(−NHNH_3_^+^) + PtCl_6_^2−^ → Pt + 2(−N=NH) + 6H^+^ + 6Cl^−^ (S14)

(oxidation half-reaction) −NHNH_3_^+^ → −N=NH + 3H^+^ + 2e^−^ (S15)

(reduction half-reaction) PtCl_6_^2−^ + 4e^−^ → Pt + 6Cl^−^ (S16)

Because one −NHNH_3_^+^ group of h-PVC donates two electrons during its oxidation to the diazene (−N=NH) group, one or more −NHNH_3_^+^ groups (i.e., 1.5 for AuCl_4_^−^, 1 for PdCl_4_^2−^, and 2 for PtCl_6_^2−^) are required to reduce one PM ion.

**Note S2. Optimal pH Conditions for the PM Adsorption Tests**

The optimal pH for the PM adsorption tests was determined by characterizing the *R*_e_ value of the h-PVC polymer as a function of the solution pH (Figure S12, Supporting Information). The h-PVC polymer exhibited a higher *R*_e_ at a lower pH owing to its enhanced positive charge, which promoted its electrostatic attraction with anionic PM species.^[5]^ The *R*_e_ value for Pd was measurable only at pH <5 because Pd ions are precipitated at pH ≥5.^[6]^

**Note S3. PM Reduction Abilities of the h-PVC Polymer and Reducing Agents**

The PM reduction abilities of the h-PVC polymer and reducing agents (hydrazine and NaBH_4_) were characterized by examining PM (200 mg L^−1^) aqueous solutions (50 mL; pH = 2) to which the same dose (0.2 g L^−1^) of the h-PVC polymer, hydrazine, or NaBH_4_ was introduced. Similar to the h-PVC polymer, both reducing agents reduced all PM ions to PM NPs, which were precipitated, except for hydrazine in the Pt aqueous solution, in which no Pt reduction was observed (Figures S13 and S14, Supporting Information). This result indicates that hydrazine has a significantly lower ability to reduce Pt ions than the h-PVC polymer and NaBH_4_, which is supported by a previous finding that highly concentrated hydrazine is required to reduce Pt ions.^[7]^ Consistent with this finding, we also observed that Pt ions were reduced to Pt NPs only at excessively high hydrazine concentrations (≥150 g L^−1^), as shown in Figure S15 (Supporting Information).

**
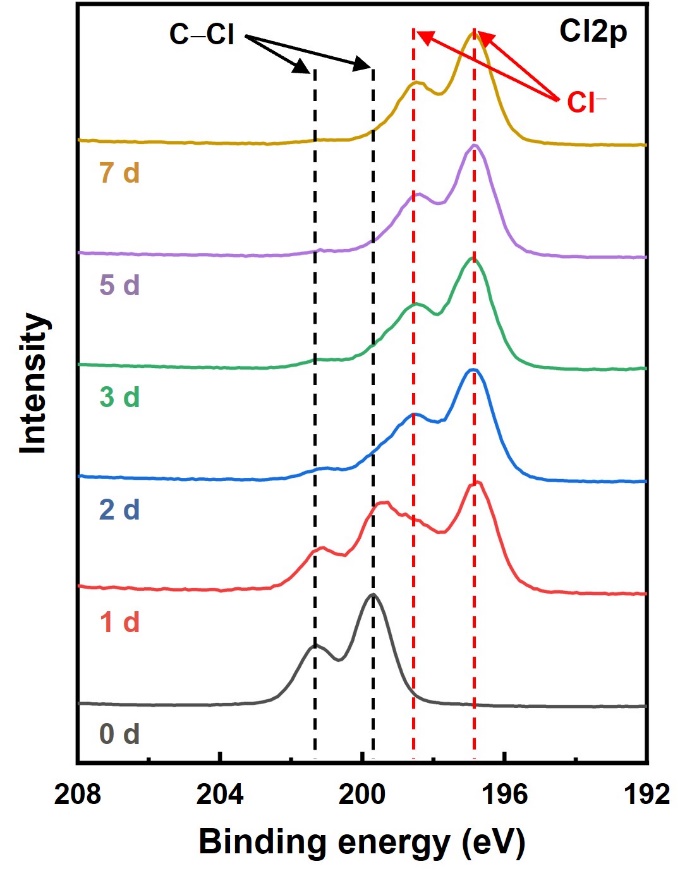
**

**Figure S1.** High-resolution Cl2p XPS peaks of the PVC polymer as a function of the hydrazination time. As the hydrazination time increased, the C−Cl bonding peaks at 201.3 and 199.7 eV were attenuated, whereas the Cl^−^ ion (counterion of the hydrazine group) peaks at 198.5 and 196.8 eV were intensified. After 5 d, the C−Cl bonding peaks completely disappeared, indicating complete hydrazination (i.e., 100% conversion).

**
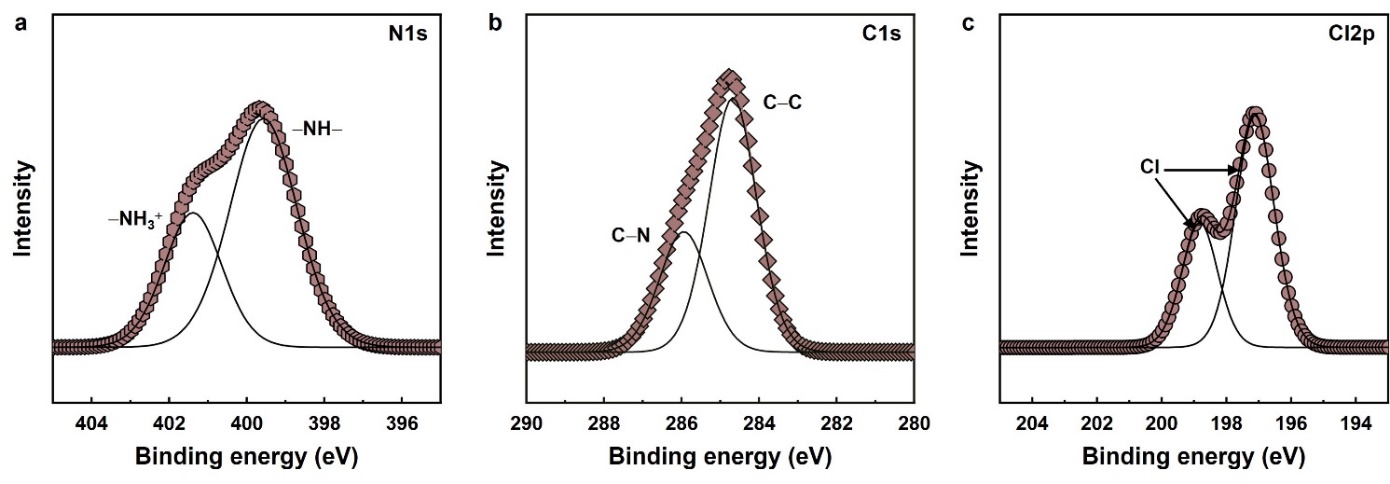
**

**Figure S2.** Deconvoluted a) N1s, b) C1s, and c) Cl2p XPS peaks of the h-PVC polymer. The spectra are consistent with the chemical structure of the h-PVC polymer.

**
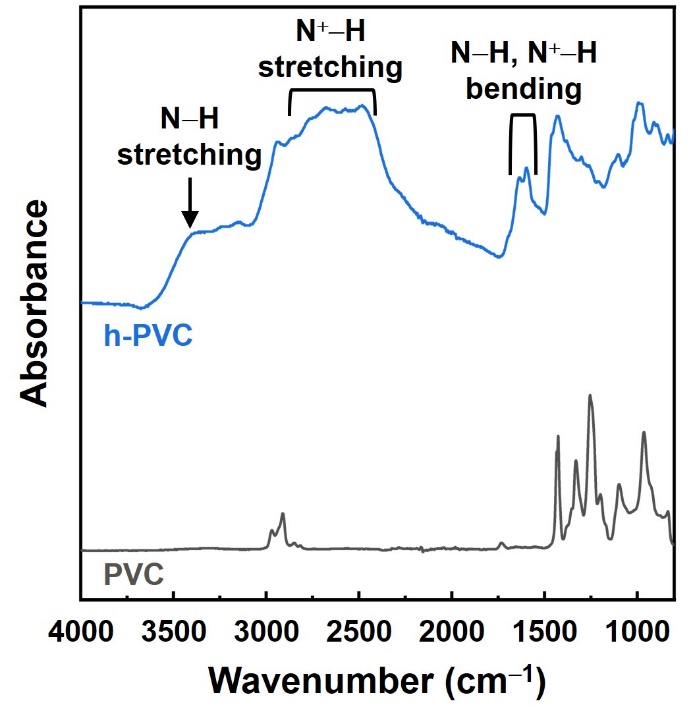
**

**Figure S3.** FT-IR spectra of the PVC and h-PVC polymers. The h-PVC polymer exhibited FT-IR peaks at 3300 (N−H amine stretching), 2480−2850 (N^+^−H protonated amine stretching), 1636 (N−H amine bending), and 1597 (N^+^−H protonated amine bending)^[8]^ cm^−1^, which were absent in the spectra of the pristine PVC polymer, thus confirming complete hydrazination.

**
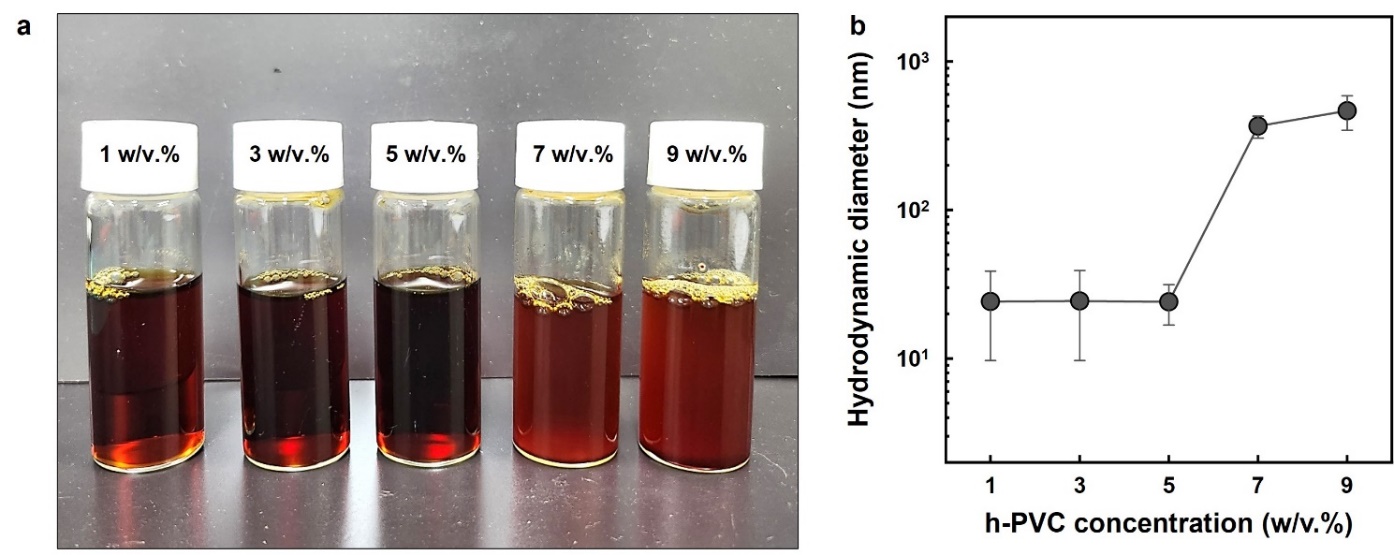
**

**Figure S4.** a) Photographs and b) hydrodynamic diameters of the h-PVC aqueous solutions as a function of the h-PVC concentration. The hydrodynamic diameter of the h-PVC polymer was analyzed using dynamic light scattering (ELSZ-2000, Otsuka Electronics). The solutions with h-PVC concentrations above 5 w/v.% revealed a significant increase in the hydrodynamic diameter of the h-PVC polymer owing to its limited solubility in water (5 w/v.%). Data represents the mean ± standard deviation (*n* = 3).

**
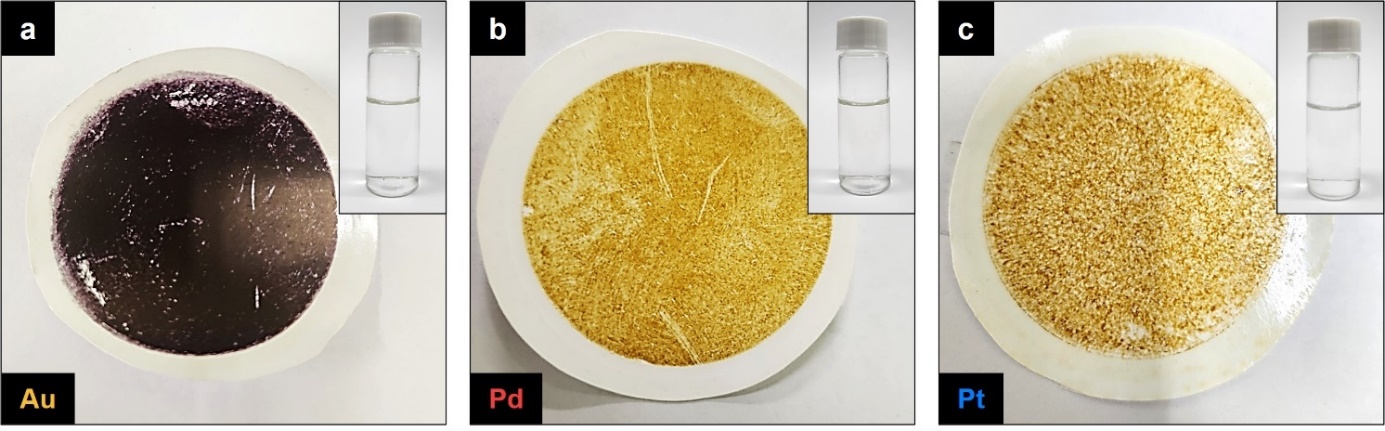
**

**Figure S5.** Photographs of the PM@h-PVC precipitates collected by PSU membrane filtration after PM (200 mg L^−1^) aqueous solutions (pH = 2) containing the h-PVC polymer (0.2 g L^−1^) were shaken for 3 h: a) Au, b) Pd, and c) Pt. Insets: Photographs of the permeate solutions.

**
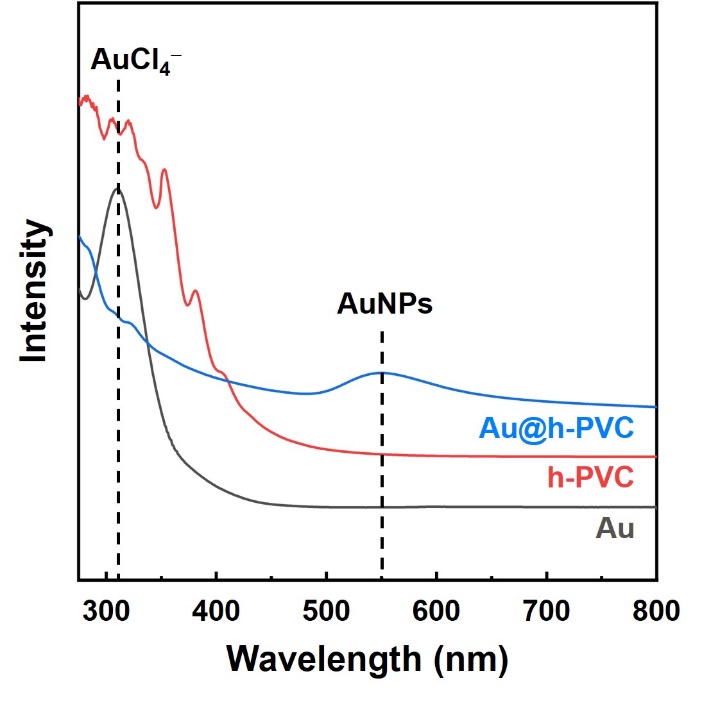
**

**Figure S6.** UV−vis spectra of aqueous solutions containing Au ions (AuCl_4_^−^, 200 mg L^−1^) only, the h-PVC polymer only, and the Au ions/h-PVC polymer (containing Au@h-PVC precipitates) (h-PVC polymer dose = 0.2 g L^−1^, pH = 2, contact time = 3 h).


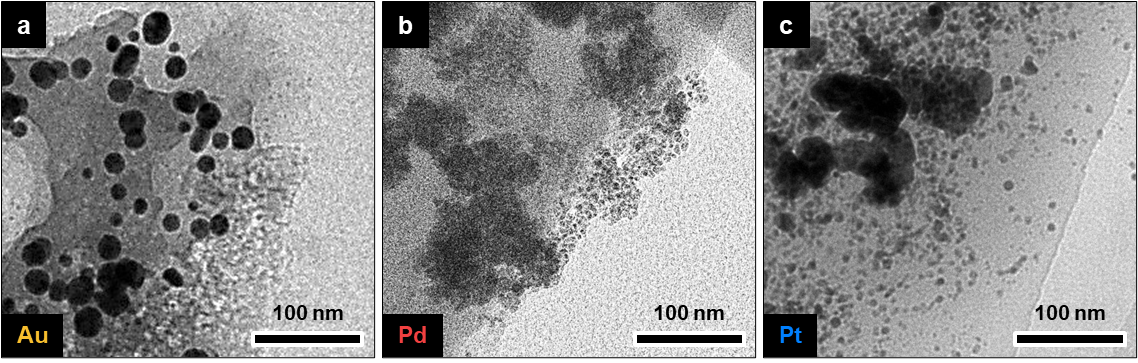


**Figure S7.** Low-magnification TEM images of the PM@h-PVC precipitates: a) Au, b) Pd, and c) Pt. The PM@h-PVC precipitates were collected polysulfone membrane filtration after PM (200 mg L^−1^) aqueous solutions (pH = 2) containing the h-PVC polymer (0.2 g L^−1^) were shaken for 3 h. PM NPs are clearly visible in the corresponding PM@h-PVC precipitates.

**
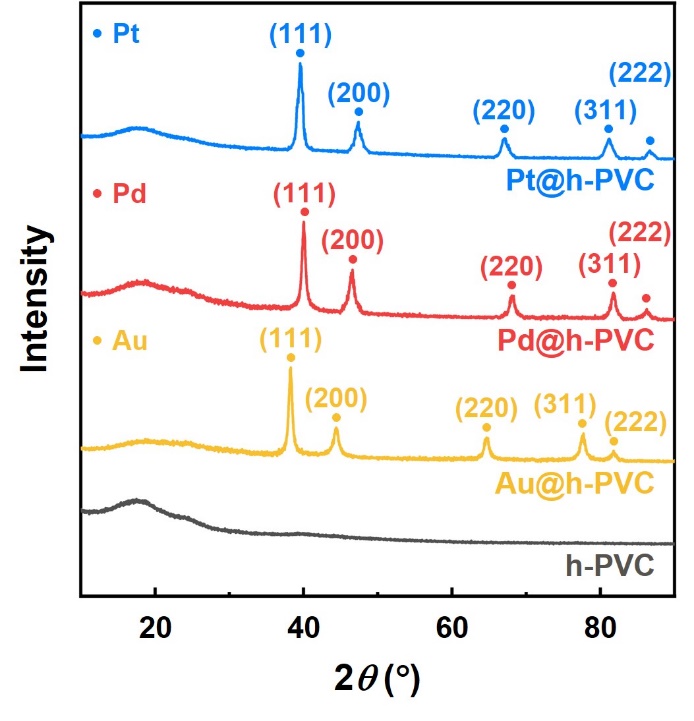
**

**Figure S8.** XRD patterns of the h-PVC polymer and PM@h-PVC precipitates. The PM@h-PVC precipitates were collected by PSU membrane filtration after PM (200 mg L^−1^) aqueous solutions (pH = 2) containing the h-PVC polymer (0.2 g L^−1^) were shaken for 3 h.

**
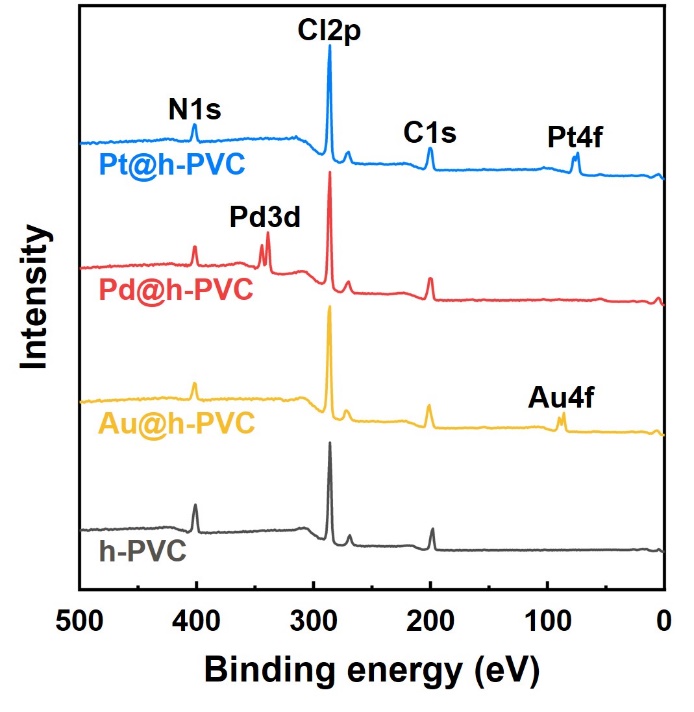
**

**Figure S9.** XPS survey spectra of the h-PVC polymer and PM@h-PVC precipitates. The PM@h-PVC precipitates were collected by PSU membrane filtration after PM (200 mg L^−1^) aqueous solutions (pH = 2) containing the h-PVC polymer (0.2 g L^−1^) were shaken for 3 h.

**
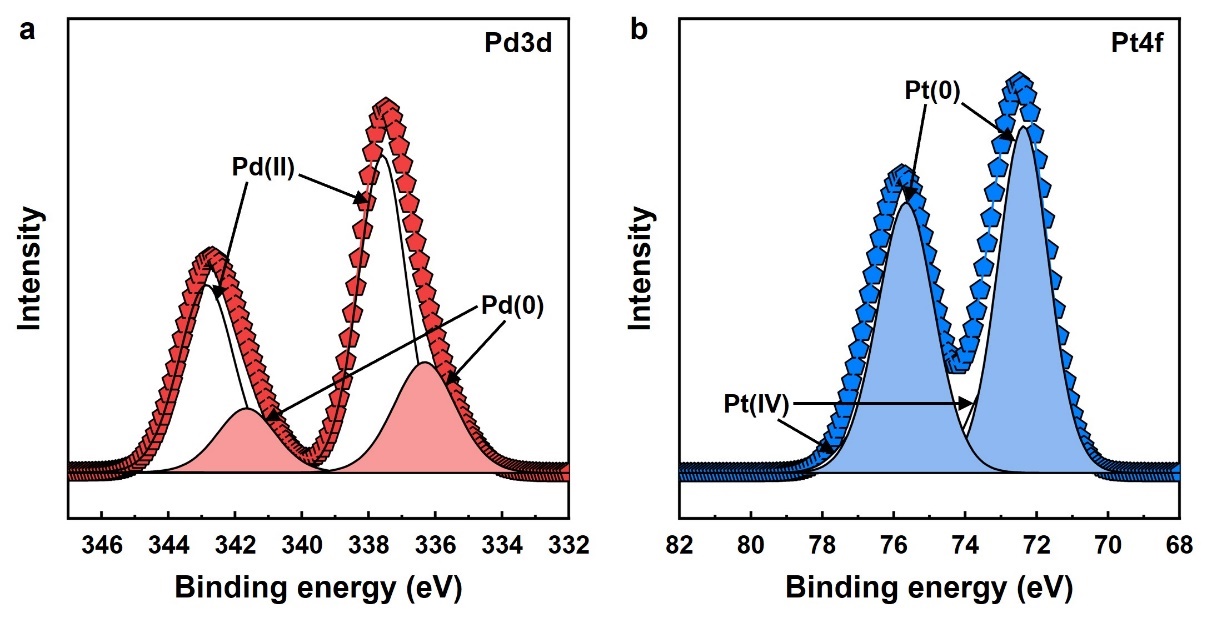
**

**Figure S10.** Deconvoluted a) Pd3d and b) Pt4f XPS peaks of the Pd@h-PVC and Pt@h-PVC precipitates, respectively. The PM@h-PVC precipitates were collected by PSU membrane filtration after PM (200 mg L^−1^) aqueous solutions (pH = 2) containing the h-PVC polymer (0.2 g L^−1^) were shaken for 3 h.

**
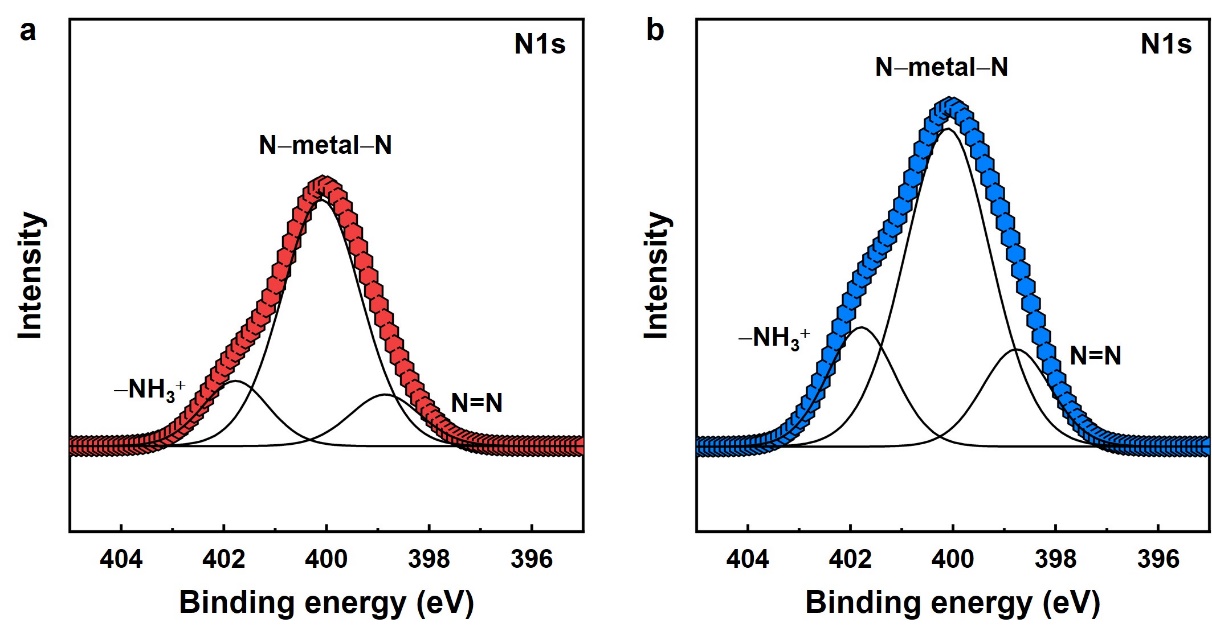
**

**Figure S11.** Deconvoluted N1s XPS peaks of the a) Pd@h-PVC and b) Pt@h-PVC precipitates. The PM@h-PVC precipitates were collected by PSU membrane filtration after PM (200 mg L^−1^) aqueous solutions (pH = 2) containing the h-PVC polymer (0.2 g L^−1^) were shaken for 3 h.

**
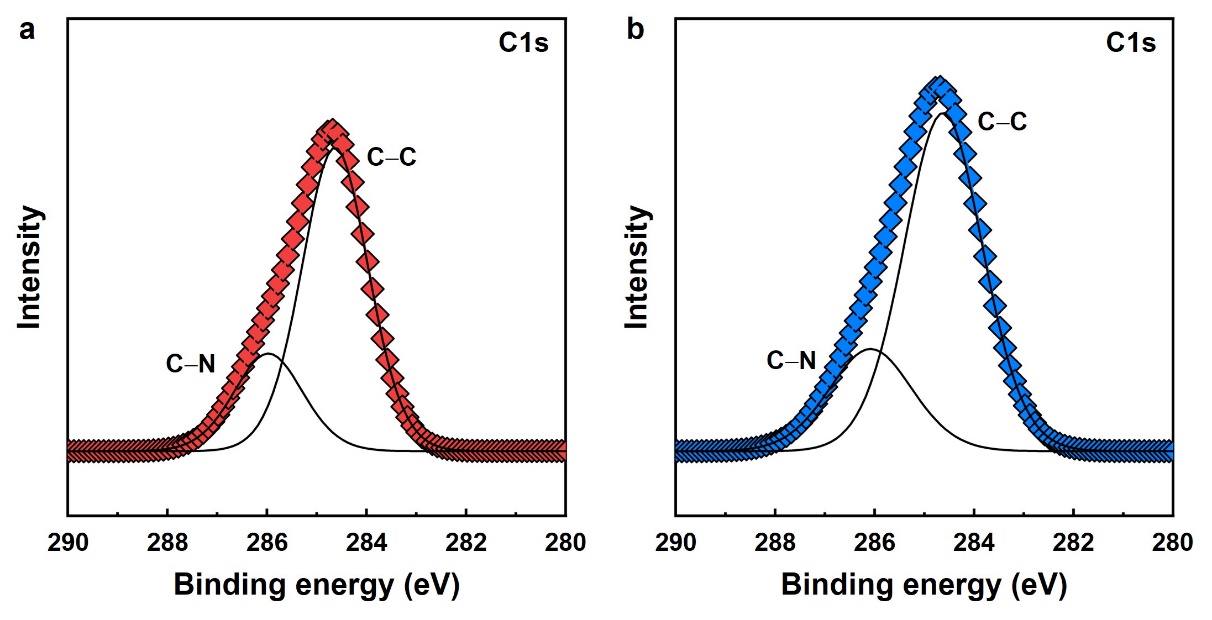
**

**Figure S12.** Deconvoluted C1s XPS peaks of the a) Pd@h-PVC and b) Pt@h-PVC precipitates. The PM@h-PVC precipitates were collected by PSU membrane filtration after PM (200 mg L^−1^) aqueous solutions (pH = 2) containing the h-PVC polymer (0.2 g L^−1^) were shaken for 3 h.

**
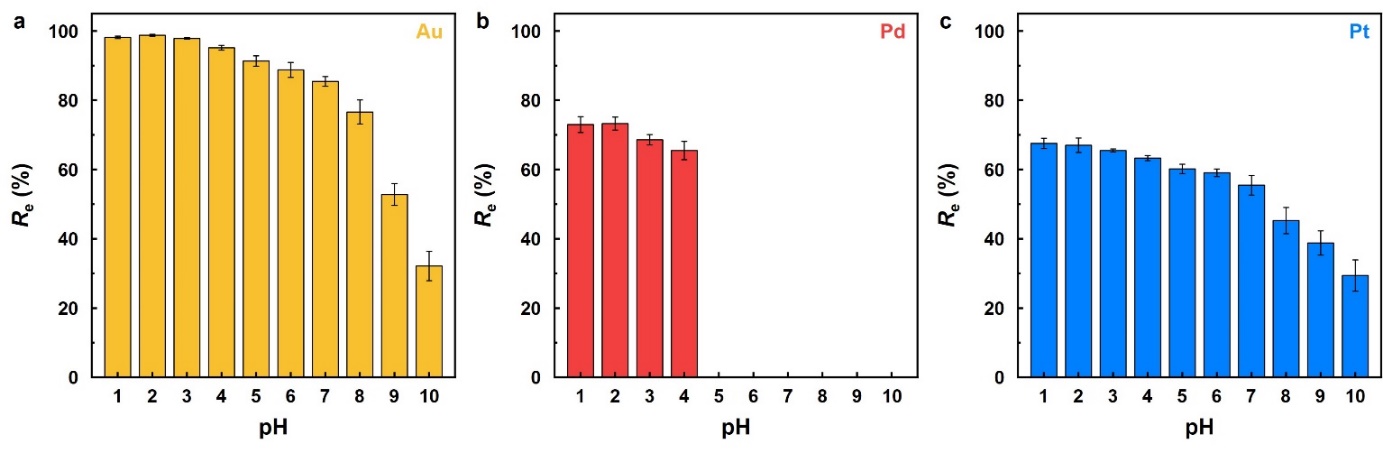
**

**Figure S13.** Recovery efficiency (*R*_e_) of the h-PVC polymer for a) Au, b) Pd, and c) Pt as a function of the solution pH (h-PVC polymer dose = 0.2 g L^−1^, initial PM ion concentration (*C*_i_) = 200 mg L^−1^, contact time = 3 h). Data represents the mean ± standard deviation (*n* = 3).

**
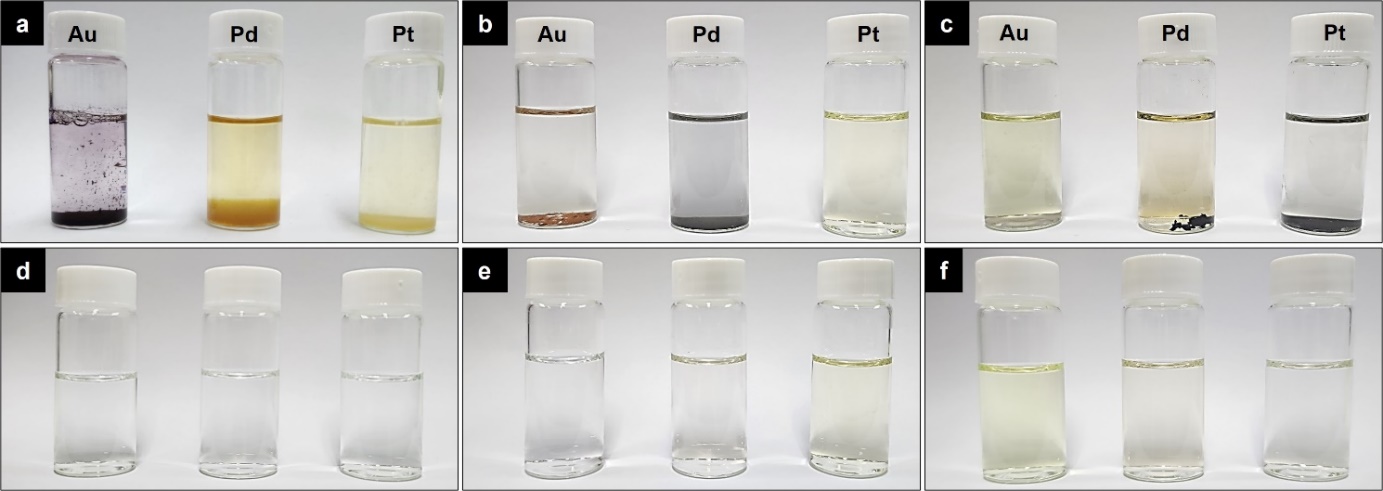
**

**Figure S14.** Photographs of PM (200 mg L^−1^) aqueous solutions (pH = 2) a−c) after the introduction of the h-PVC polymer and reducing agents (h-PVC polymer and reducing agent dose = 0.2 g L^−1^, contact time = 3 h) and d−f) their corresponding permeate solutions, which were collected by PSU membrane filtration: a,d) h-PVC polymer, b,e) hydrazine, and c,f) NaBH_4_.

**
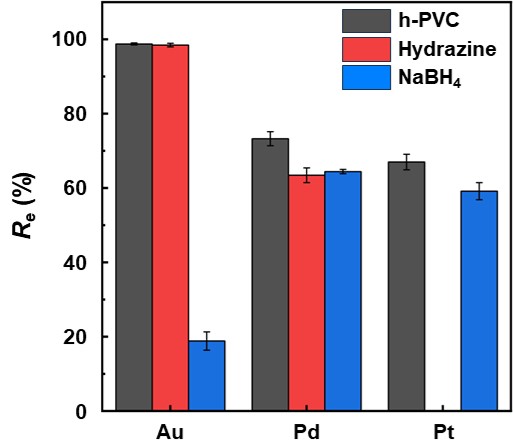
**

**Figure S15.** Recovery efficiency (*R*_e_) of the h-PVC polymer and reducing agents (hydrazine and NaBH_4_) for Au, Pd, and Pt (h-PVC polymer and reducing agent dose = 0.2 g L^−1^, *C*_i_ = 200 mg L^−1^, pH = 2, contact time = 3 h). Data represents the mean ± standard deviation (*n* = 3).

**
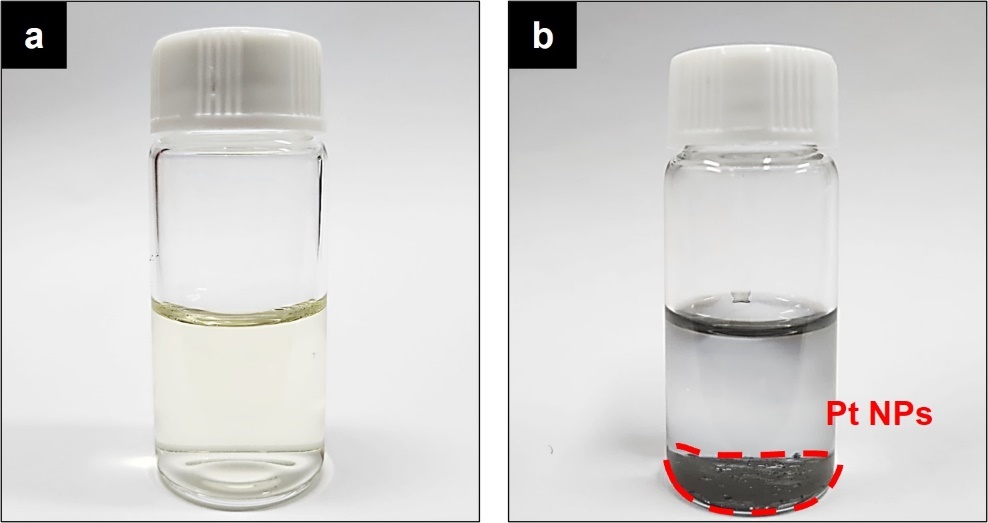
**

**Figure S16.** Photographs of Pt (200 mg L^−1^) aqueous solutions (pH = 2) after the introduction of hydrazine at doses of a) 0.2 and b) 150 g L^−1^ (contact time = 3 h).


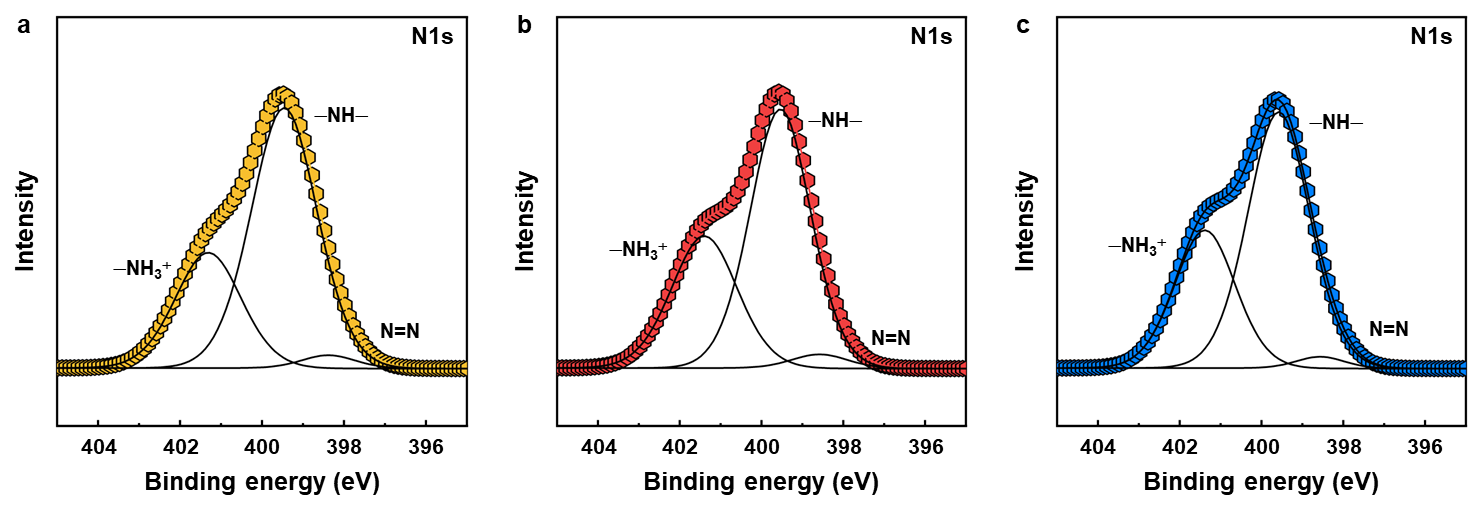


**Figure S17.** Deconvoluted N1s XPS peaks of the h-PVC polymers obtained after five PM adsorption−desorption cycles: a) Au, b) Pd, and c) Pt. Despite its marginal N=N peak, the N1s spectrum of the h-PVC polymer after five PM adsorption−desorption cycles was nearly identical to that of its pristine form (Figure S2a, Supporting Information) regardless of the PM type. This result indicates that most N=N groups are reduced back to hydrazine groups, and thus, the chemical structure of the h-PVC polymer remains nearly intact during five adsorption−desorption cycles, demonstrating its excellent structural stability.

**
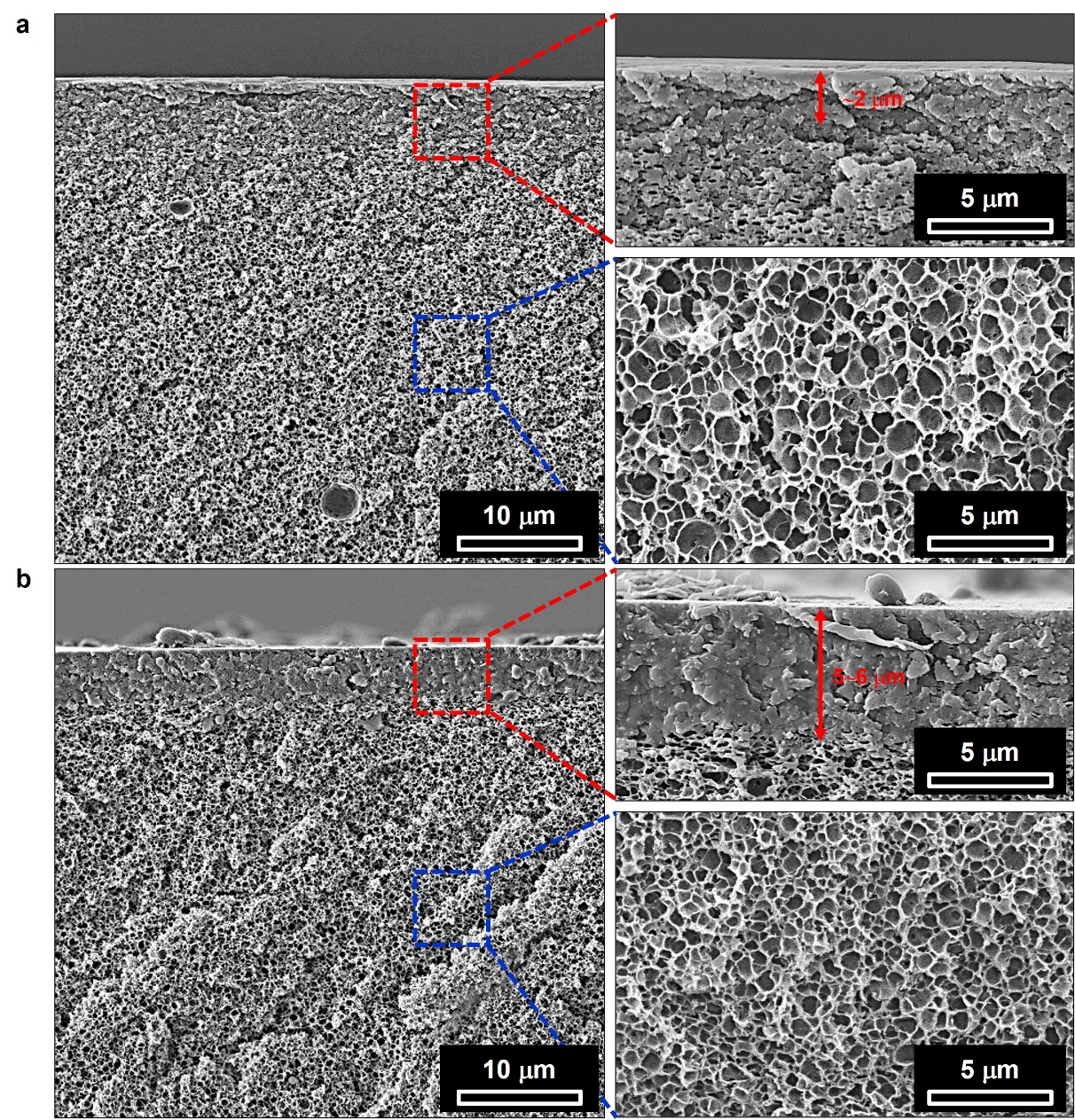
**

**Figure S18.** Cross-sectional SEM images of the a) d-PVC and b) h-PVC films.

**
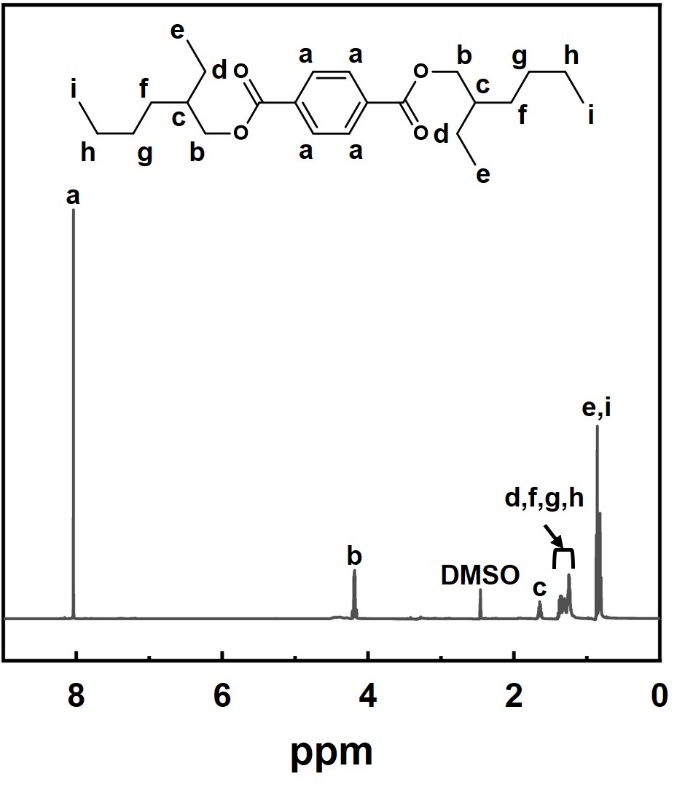
**

**Figure S19.** ^1^H NMR spectrum of the plasticizer present in the PVC film. The PVC film (1 g) was soaked in DMSO-d_6_ (10 mL) for 2 d to extract the plasticizer. The supernatant DMSO-d_6_ solution containing the plasticizer was obtained by removing the PVC film from the mixture using tweezers for ^1^H NMR analysis. The obtained ^1^H NMR spectrum identified the plasticizer as di-2-ethylhexyl terephthalate.^[9]^

Di-2-ethylhexyl terephthalate: ^1^H NMR (500 MHz, DMSO-d_6_) *δ* 0.87 (i, C−CH_3_, 3H), *δ* 0.91 (e, C−CH_3_, 3H), *δ* 1.29 (f, C−CH_2_−C, 2H), *δ* 1.30 (g, C−CH_2_−C, 2H), *δ* 1.36 (h, C−CH_2_−C, 2H), *δ* 1.41 (d, C−CH_2_−C, 2H), *δ* 1.70 (c, C−CH−C, 1H), *δ* 4.24 (b, O−CH_2_−C, 2H), *δ* 8.09 (a, benzene ring, 4H)

**
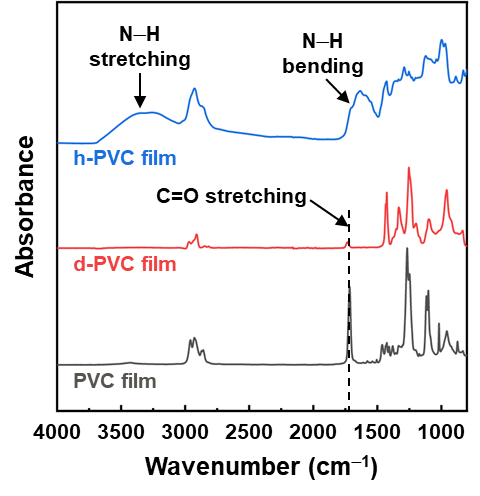
**

**Figure S20.** FT-IR spectra of the pristine PVC, d-PVC, and h-PVC (fabricated with a hydrazination time of 24 h) films. The characteristic FT-IR peak of the plasticizer (i.e., di-2-ethylhexyl terephthalate) at 1716 cm^−1^ (ester C=O stretching), which was present in the PVC film, was marginally detected in the d-PVC film. This result indicates that the plasticizer present in the PVC film was effectively removed by solvent treatment. The h-PVC film exhibited FT-IR peaks at 3300 cm^−1^ (N−H amine stretching) and 1636 cm^−1^ (N−H amine bending), which were absent in the PVC film, thus confirming its hydrazination.

**
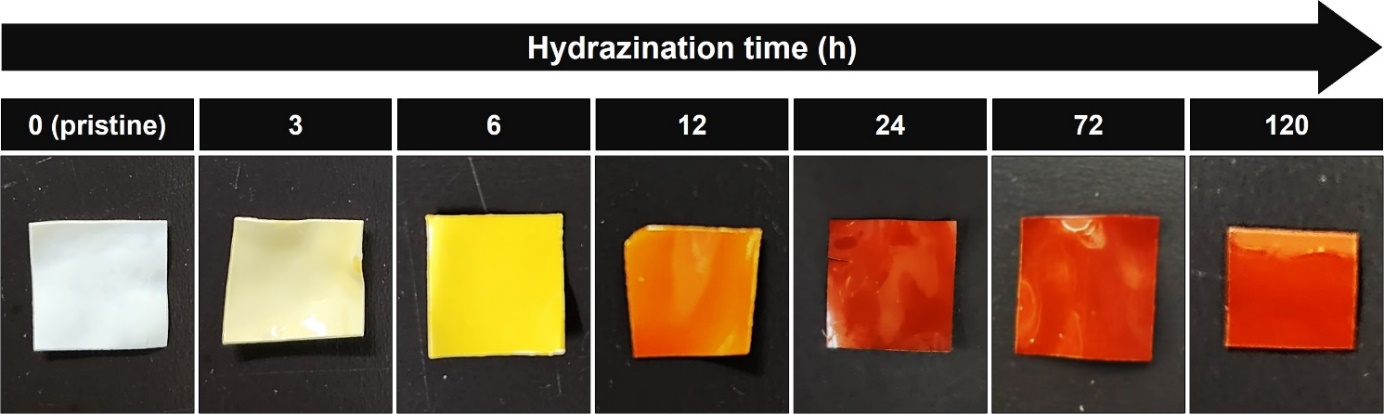
**

**Figure S21.** Photographs of the h-PVC films fabricated with different hydrazination times. The pristine sample represents the d-PVC film. Longer hydrazination times resulted in the formation of h-PVC films that were more distinctly brown, which is the characteristic color of the h-PVC polymer, by increasing the hydrazination degree.


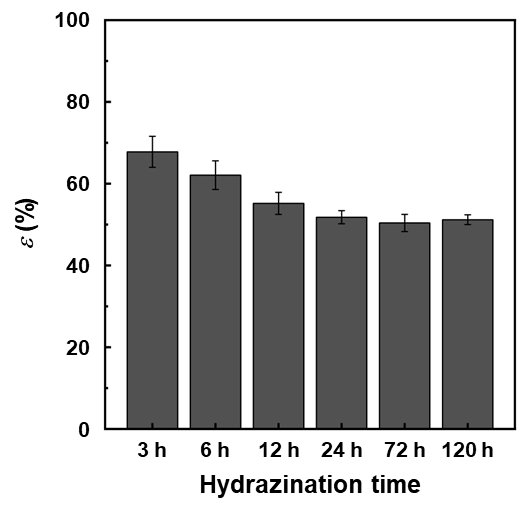


**Figure S22.** Overall porosity (*ε*) of the h-PVC films fabricated with different hydrazination times. Data represents the mean ± standard deviation (*n* = 3).

**
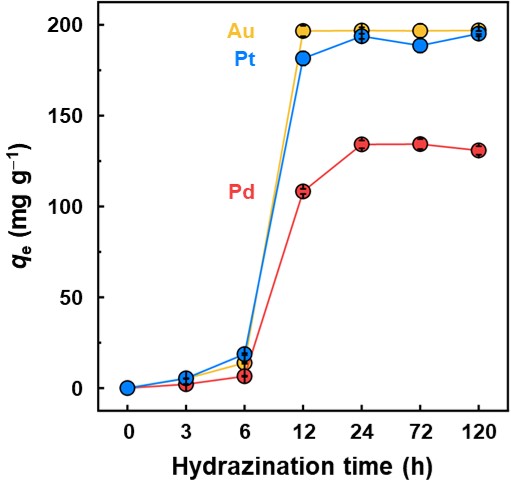
**

**Figure S23.** Equilibrium PM adsorption capacity (*q*_e_) of the h-PVC films fabricated with different hydrazination times (h-PVC film dose = 1.0 g L^−1^, *C*_i_ = 200 mg L^−1^, pH = 2, contact time = 12 h). The optimal hydrazination time was determined to be 24 h, at which the *q*_e_ value of the resultant h-PVC film was saturated. Data represents the mean ± standard deviation (*n* = 3).


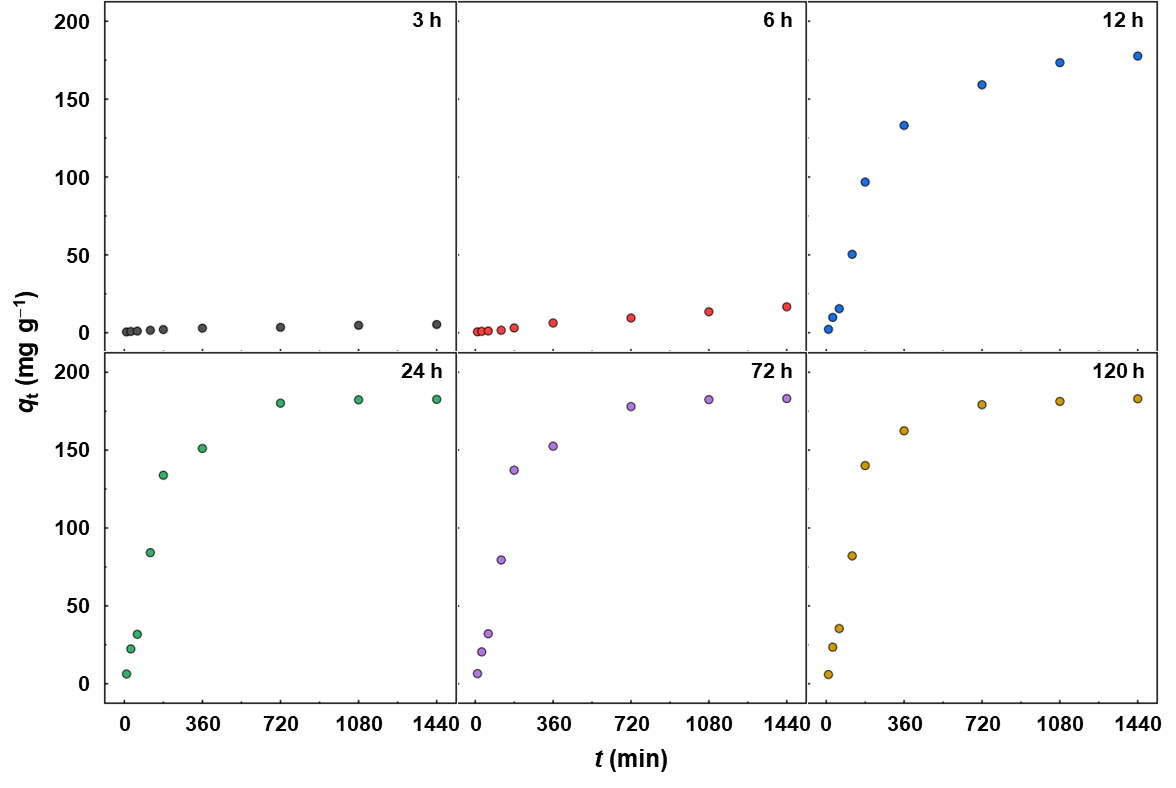


**Figure S24.** Au adsorption kinetics (Au adsorption capacity (*q*_t_) as a function of the contact time (*t*)) of the h-PVC films fabricated with different hydrazination times (h-PVC film dose = 1.0 g L^−1^, *C*_i_ = 200 mg L^−1^, pH = 2). The h-PVC films fabricated with short hydrazination times (3 and 6 h) displayed significantly low Au adsorption rate and capacity. The h-PVC films fabricated with long hydrazination times (24 h and longer) showed nearly identical Au adsorption kinetics and equilibrium adsorption capacity.

**
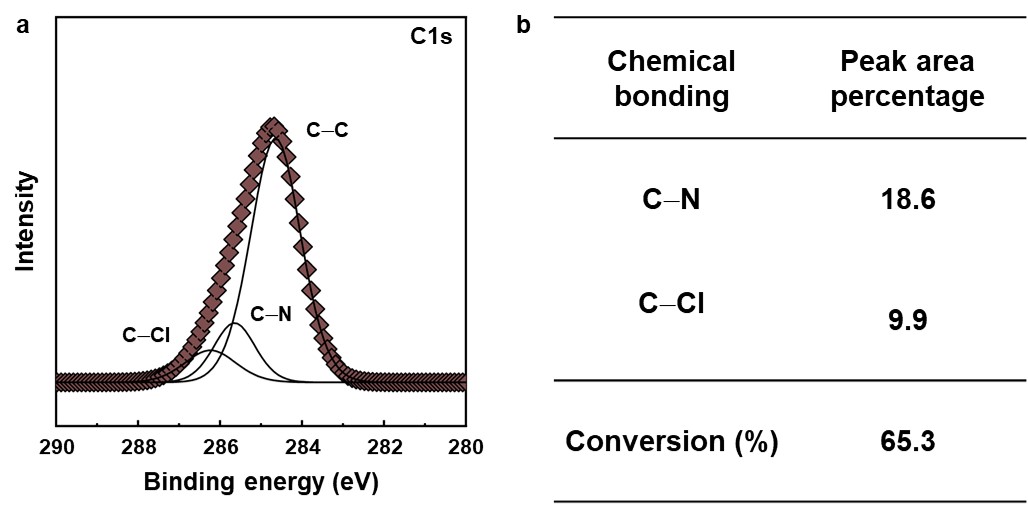
**

**Figure S25.** a) Deconvoluted C1s XPS peaks of the h-PVC film fabricated with a hydrazination time of 24 h and b) their area percentages and calculated conversion of Cl to hydrazine groups. The h-PVC film was dissolved in *N*-methylpyrrolidone at 80 °C for 24 h and precipitated in acetone. The obtained precipitates were vacuum-dried for 24 h and analyzed using XPS.

**
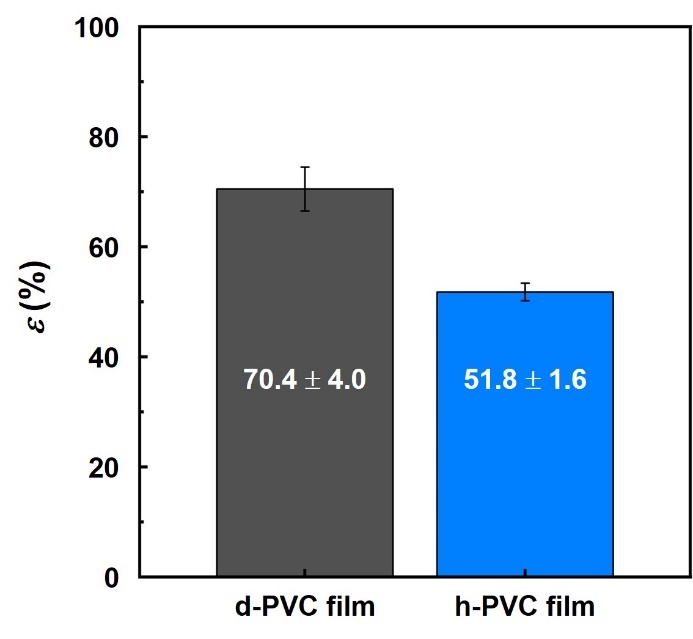
**

**Figure S26.** Overall porosity (*ε*) of the d-PVC and h-PVC films. Data represents the mean ± standard deviation (*n* = 3).

**
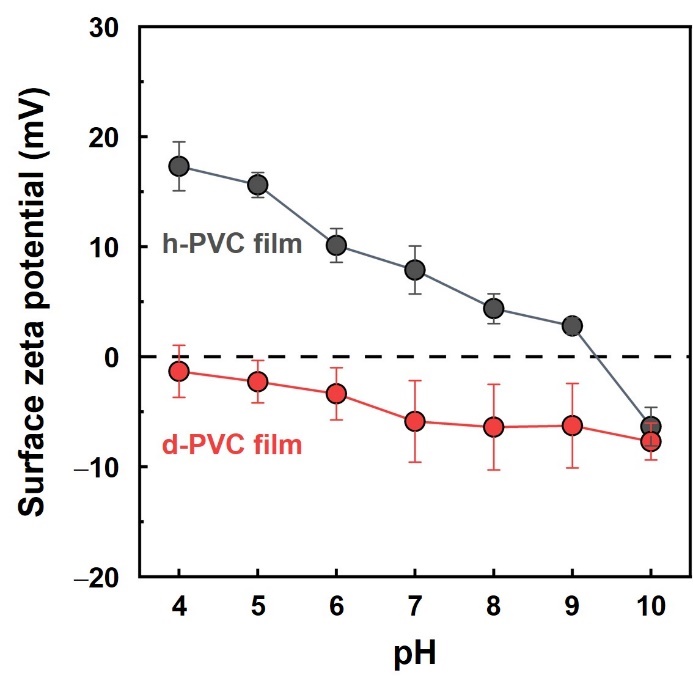
**

**Figure S27.** Surface zeta potentials of the d-PVC and h-PVC films as a function of the solution pH. Data represents the mean ± standard deviation (*n* = 3).

**
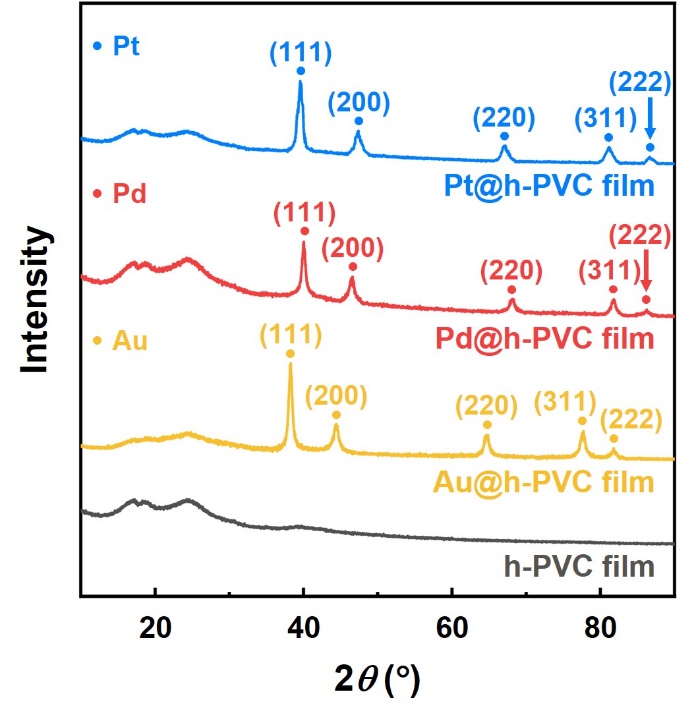
**

**Figure S28.** XRD patterns of the h-PVC and PM@h-PVC films. The PM@h-PVC films were collected using tweezers after PM (200 mg L^−1^) aqueous solutions (pH = 2) containing the h-PVC film (1.0 g L^−1^) were shaken for 12 h.

**
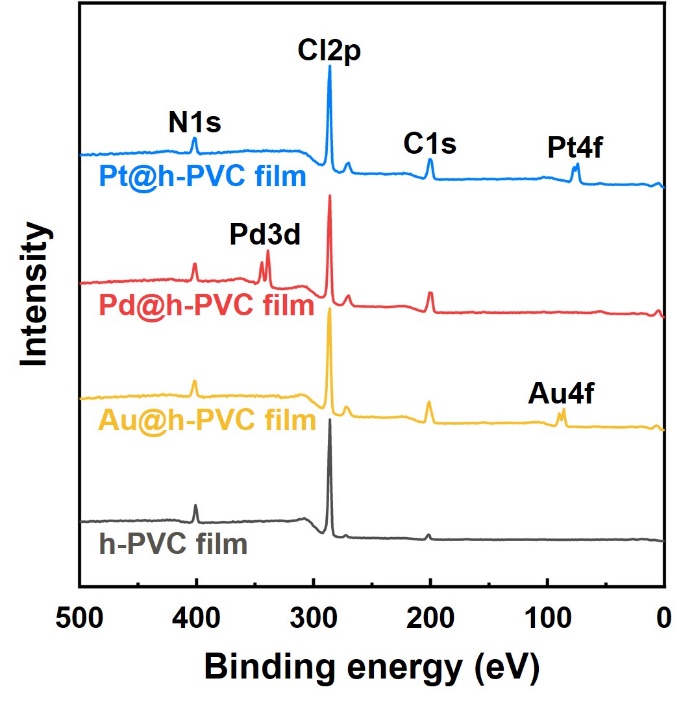
**

**Figure S29.** XPS survey spectra of the h-PVC and PM@h-PVC films. The PM@h-PVC films were collected using tweezers after PM (200 mg L^−1^) aqueous solutions (pH = 2) containing the h-PVC film (1.0 g L^−1^) were shaken for 12 h.

**
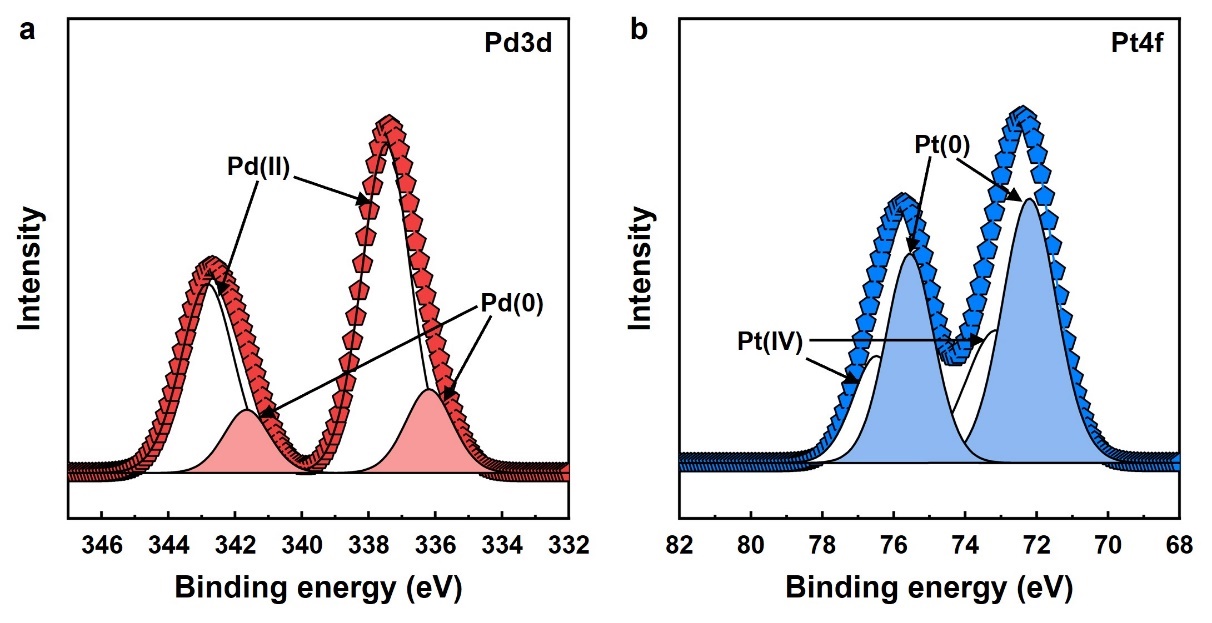
**

**Figure S30.** Deconvoluted a) Pd3d and b) Pt4f XPS peaks of the Pd@h-PVC and Pt@h-PVC films, respectively. The PM@h-PVC films were collected using tweezers after PM (200 mg L^−1^) aqueous solutions (pH = 2) containing the h-PVC film (1.0 g L^−1^) were shaken for 12 h.

**
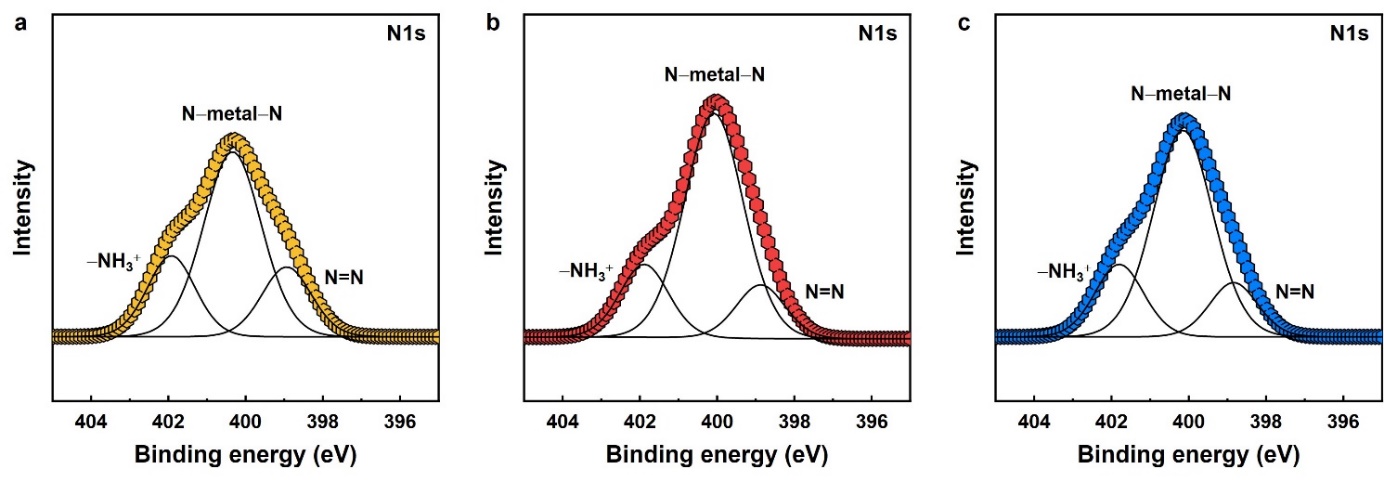
**

**Figure S31.** Deconvoluted N1s XPS peaks of the a) Au@h-PVC, b) Pd@h-PVC, and c) Pt@h-PVC films. The PM@h-PVC films were collected using tweezers after PM (200 mg L^−1^) aqueous solutions (pH = 2) containing the h-PVC film (1.0 g L^−1^) were shaken for 12 h.

**
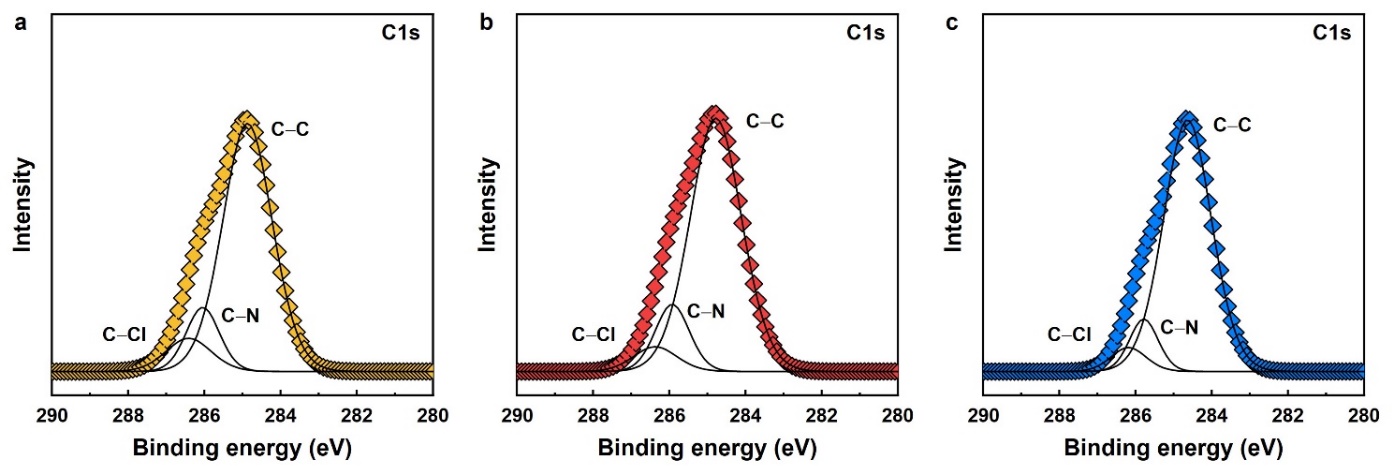
**

**Figure S32.** Deconvoluted C1s XPS peaks of the a) Au@h-PVC, b) Pd@h-PVC, and c) Pt@h-PVC films. The PM@h-PVC films were collected using tweezers after PM (200 mg L^−1^) aqueous solutions (pH = 2) containing the h-PVC film (1.0 g L^−1^) were shaken for 12 h.

**
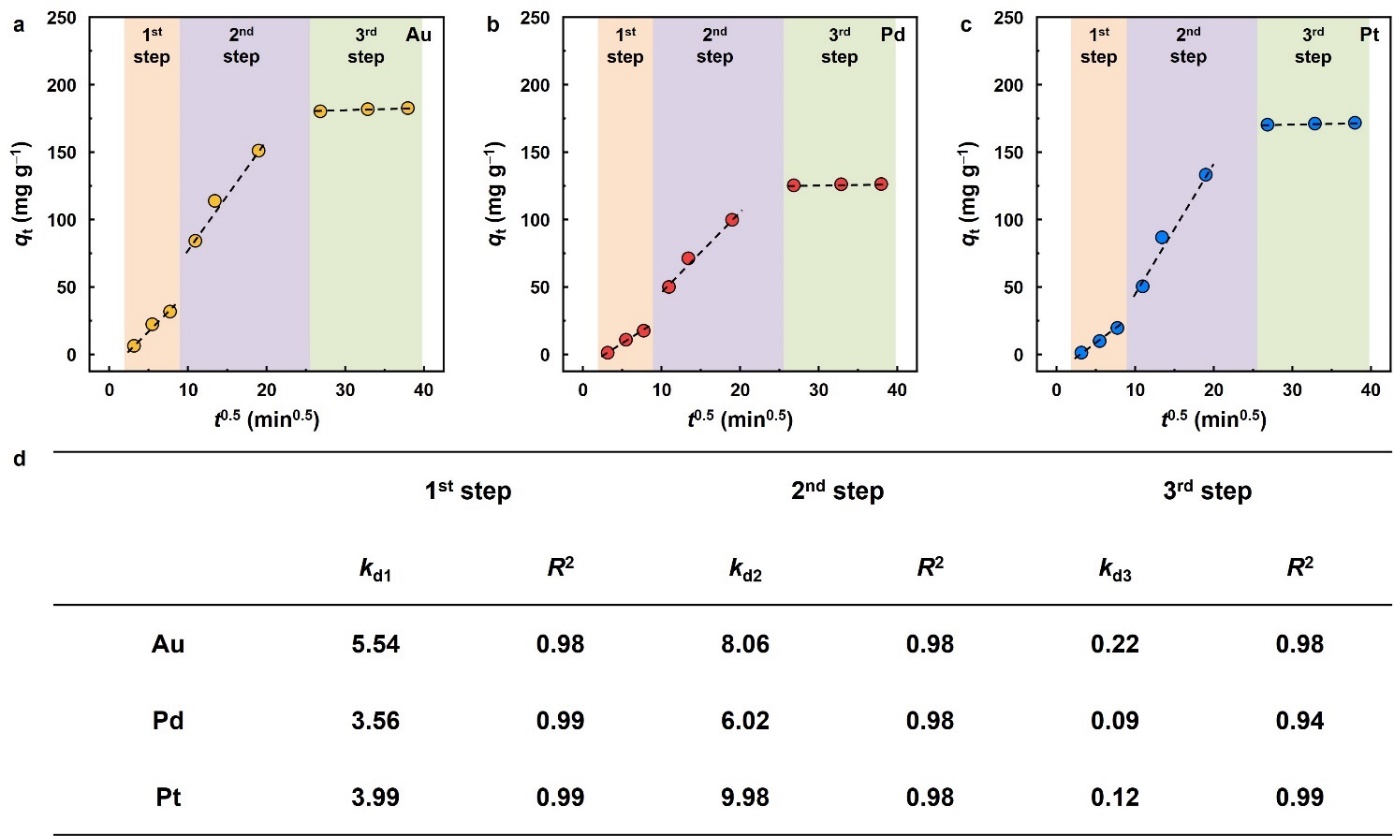
**

**Figure S33.** a−c) PM adsorption kinetics data of the h-PVC film fitted to the Weber−Morris model: a) Au, b) Pd, and c) Pt. d) Corresponding model fitting parameters for the three PMs.

**
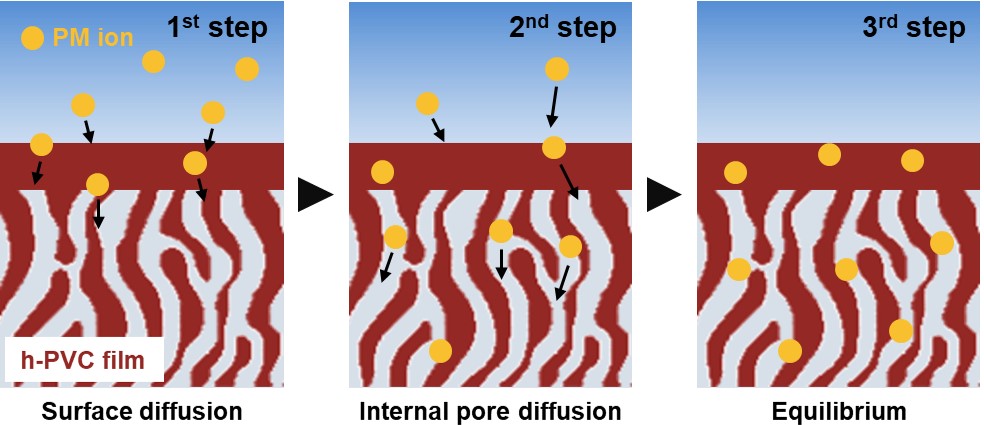
**

**Figure S34.** Schematic of the kinetics of the PM adsorption mechanism of the h-PVC film.

**
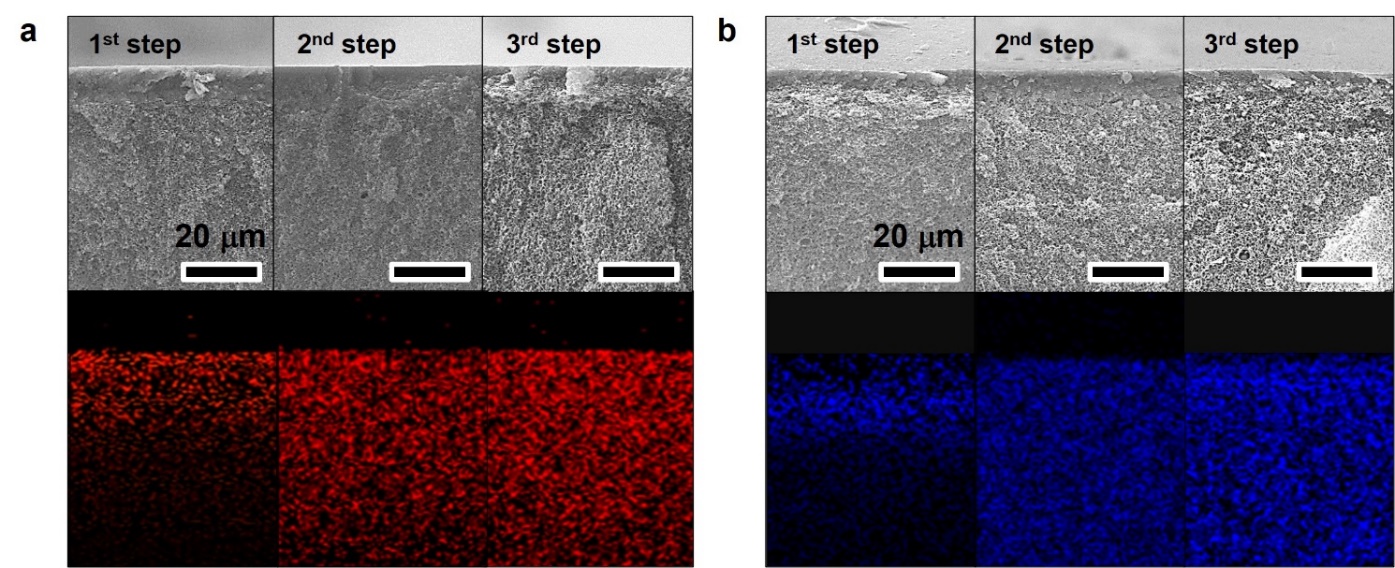
**

**Figure S35.** Cross-sectional SEM (top) and corresponding EDS (bottom) images of the a) Pd@h-PVC and b) Pt@h-PVC films over three-step adsorption: (h-PVC film dose = 1.0 g L^−1^, *C*_i_ = 200 mg L^−1^, pH = 2, contact time = 10 min (1^st^ step), 2 h (2^nd^ step), and 12 h (3^rd^ step)).

**
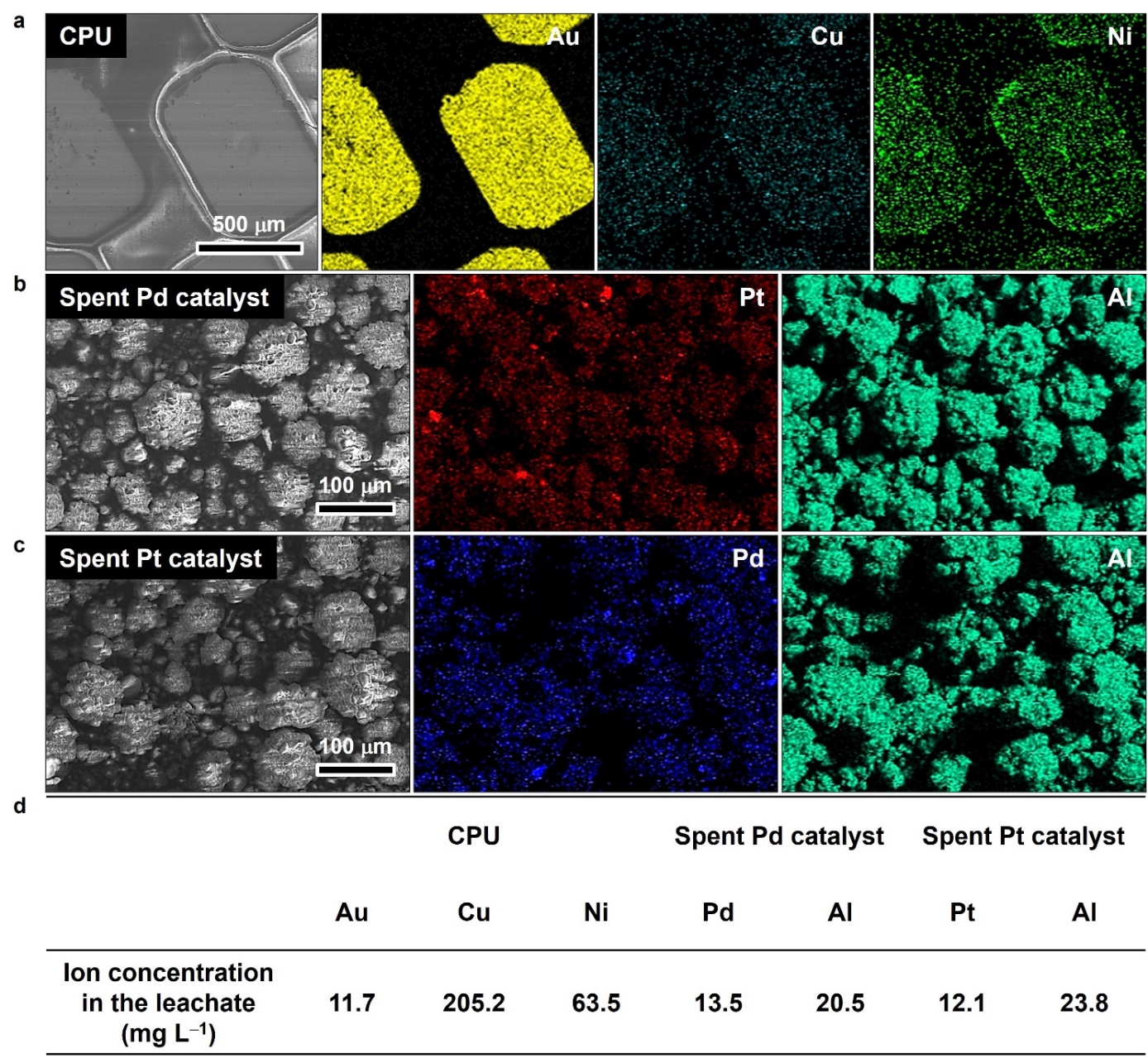
**

**Figure S36.** a−c) SEM (left) and corresponding EDS (right) images of the spent a) CPU and b) Pd and c) Pt catalysts. d) Metal composition and ion concentration of the real-world leachates.


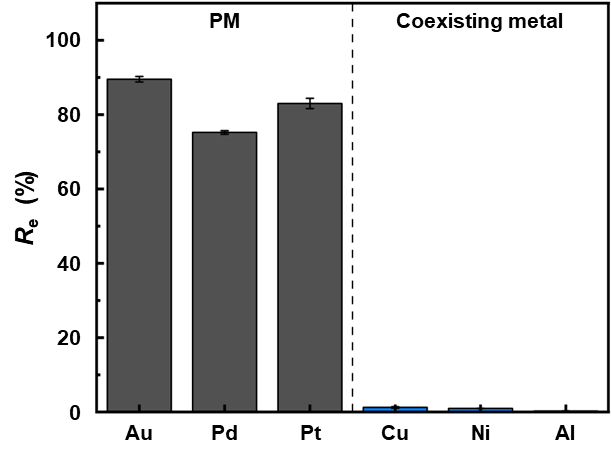


**Figure S37.** Recovery efficiency (*R*_e_) of the h-PVC film for each metal ion (h-PVC film dose = 1.0 g L^−1^, *C*_i_ = 100 mg L^−1^, pH = 2, contact time = 12 h). The h-PVC film exhibited high *R*_e_ (>75%) for anionic PM species but marginal *R*_e_ (<1.3%) for metal cations (i.e., Cu^2+^, Ni^2+^, and Al^3+^), displaying its high PM selectivity. Data represents the mean ± standard deviation (*n* = 3).


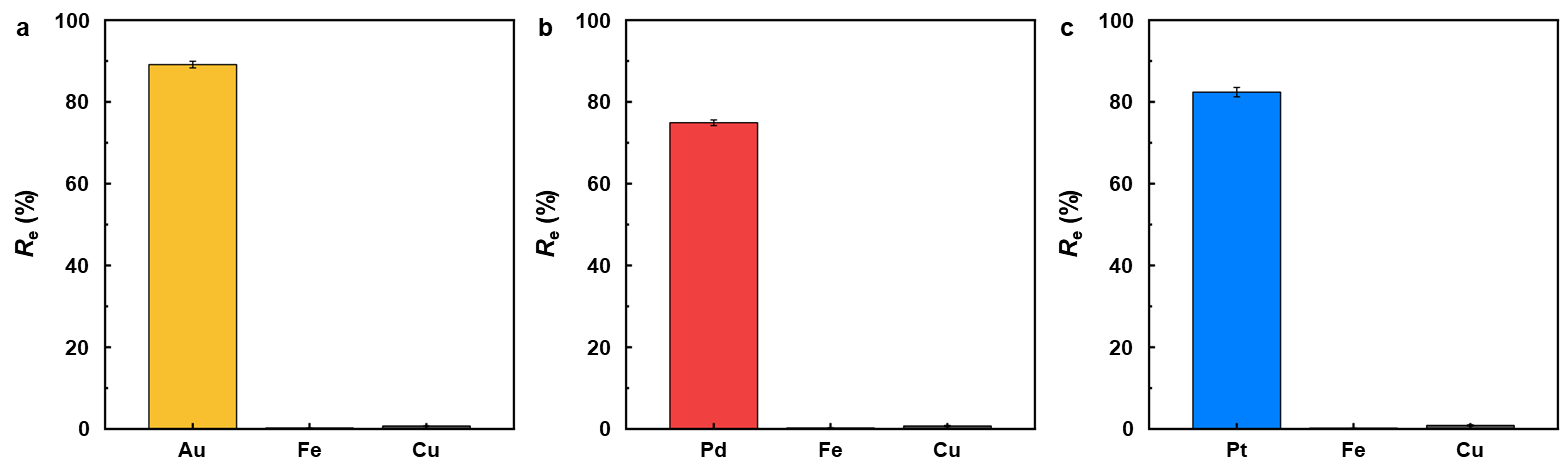


**Figure S38.** Recovery efficiency (*R*_e_) of the h-PVC film for mixed-ion solutions containing PM, Fe, and Cu ions: a) Au, b) Pd, and c) Pt (h-PVC film dose = 1.0 g L^−1^, *C*_i_ = 100 mg L^−1^, pH = 2, contact time = 12 h). The h-PVC film exhibited high *R*_e_ (>74.9%) for anionic PM species but marginal *R*_e_ (<0.9%) for coexisting metal cations (i.e., Fe^3+^ and Cu^2+^), displaying its high PM selectivity. Data represents the mean ± standard deviation (*n* = 3).


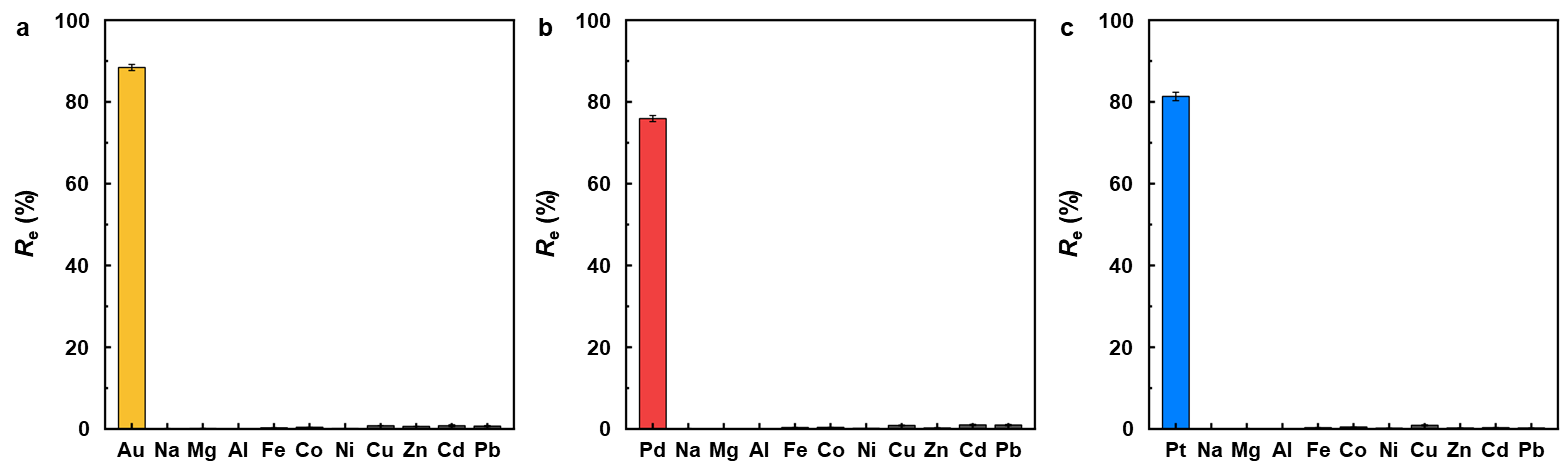


**Figure S39.** Recovery efficiency (*R*_e_) of the h-PVC film for simulated industrial wastewater containing PM and ten coexisting metal ions: a) Au, b) Pd, and c) Pt (h-PVC film dose = 1.0 g L^−1^, initial metal ion concentration (*C*_i_) = 100 mg L^−1^, pH = 2, contact time = 12 h). The h-PVC film exhibited high *R*_e_ (>75.9%) for anionic PM species but marginal *R*_e_ (<1.1%) for coexisting metal cations (i.e., Na^+^, Mg^2+^, Al^3+^, Fe^3+^, Co^2+^, Ni^2+^, Cu^2+^, Zn^2+^, Cd^2+^, and Pb^2+^), displaying its high PM selectivity. Data represents the mean ± standard deviation (*n* = 3).

**
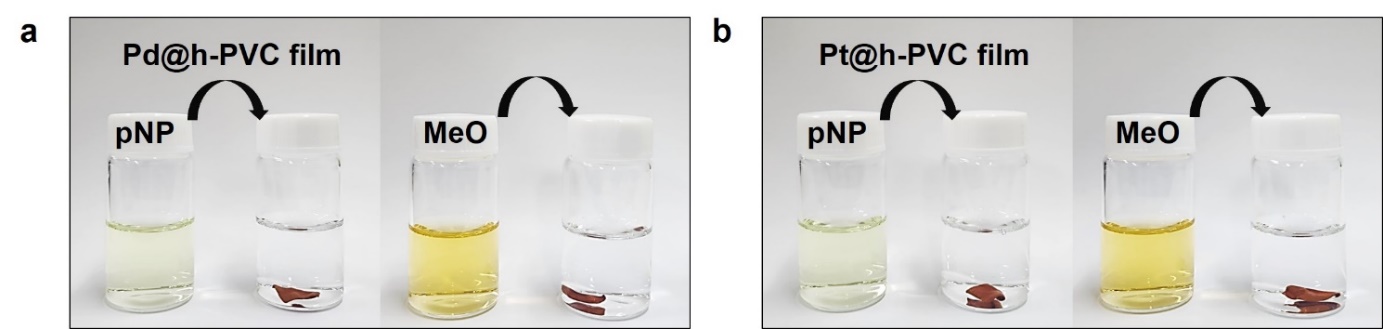
**

**Figure S40.** Photographs illustrating the reduction of organic dyes (pNP and MeO) catalyzed by the a) Pd@h-PVC and b) Pt@h-PVC films.

**
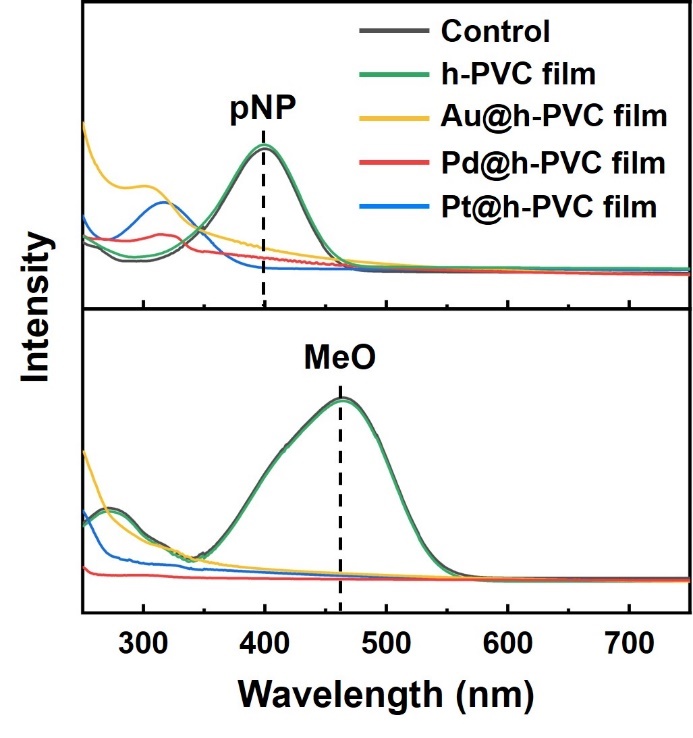
**

**Figure S41.** UV−vis spectra of organic dye (pNP and MeO) aqueous solutions containing NaBH_4_ before (control) and after the introduction of the h-PVC and PM@h-PVC films. The PM@h-PVC films were collected using tweezers after real-world leachates containing the h-PVC film (1.0 g L^−1^) were shaken for 12 h. The introduction of the PM@h-PVC films resulted in the complete disappearance of the characteristic UV−vis spectra of pNP and MeO, indicating their catalytic activity for dye reduction.


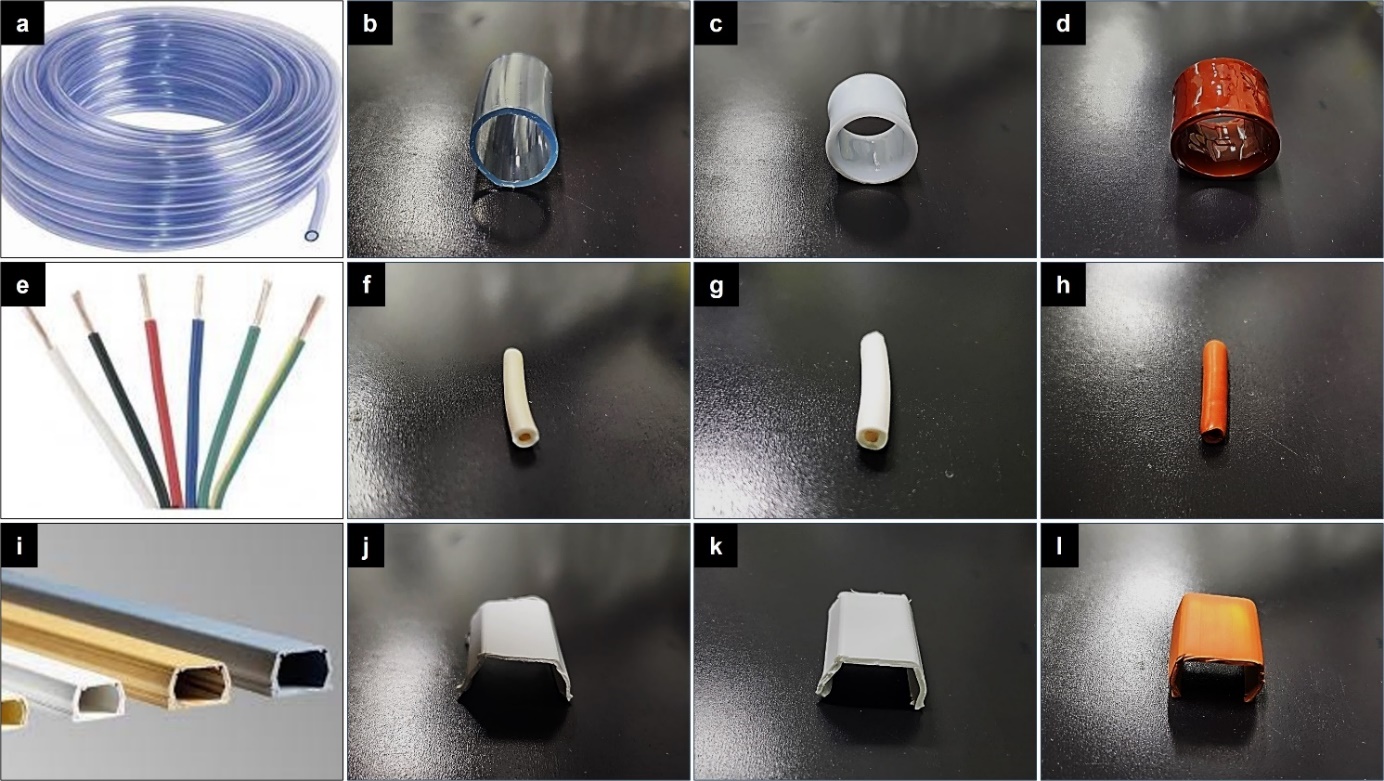


**Figure S42.** a,e,i) Conceptual images and b−d,f−h,j−l) photographs of the b,f,j) pristine PVC, c,g,k) d-PVC, and d,h,l) h-PVC plastics: a−d) PVC hose, e−h) wire sheath, and i−l) mold.


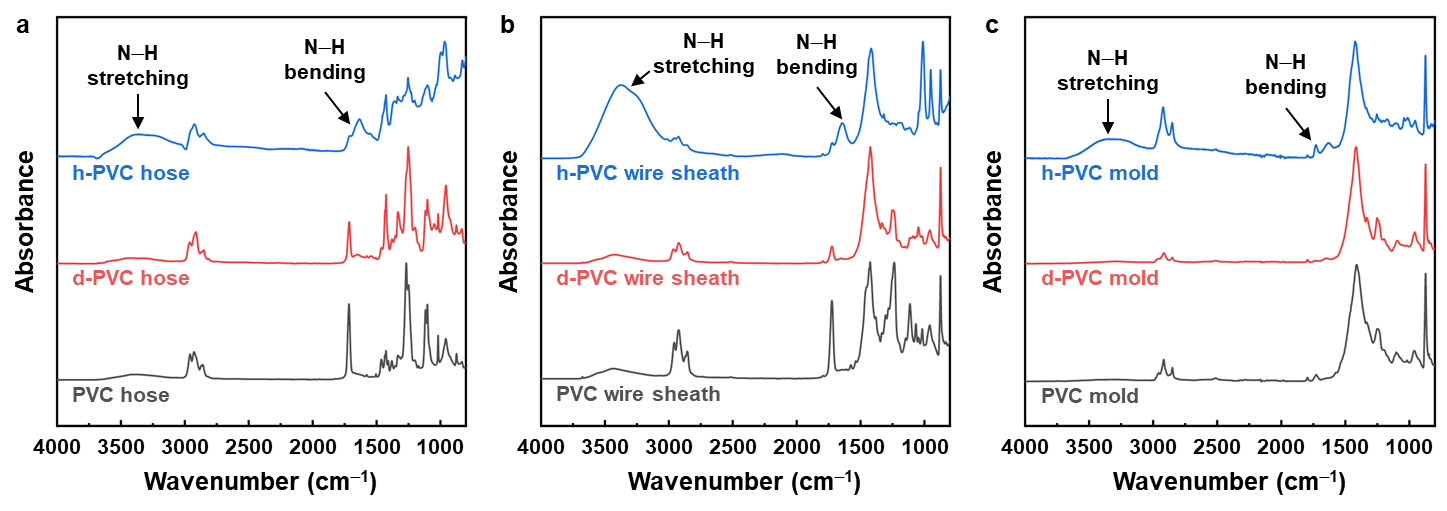


**Figure S43.** FT-IR spectra of the pristine PVC, d-PVC, and h-PVC plastics: a) PVC hose, b) wire sheath, and c) mold.


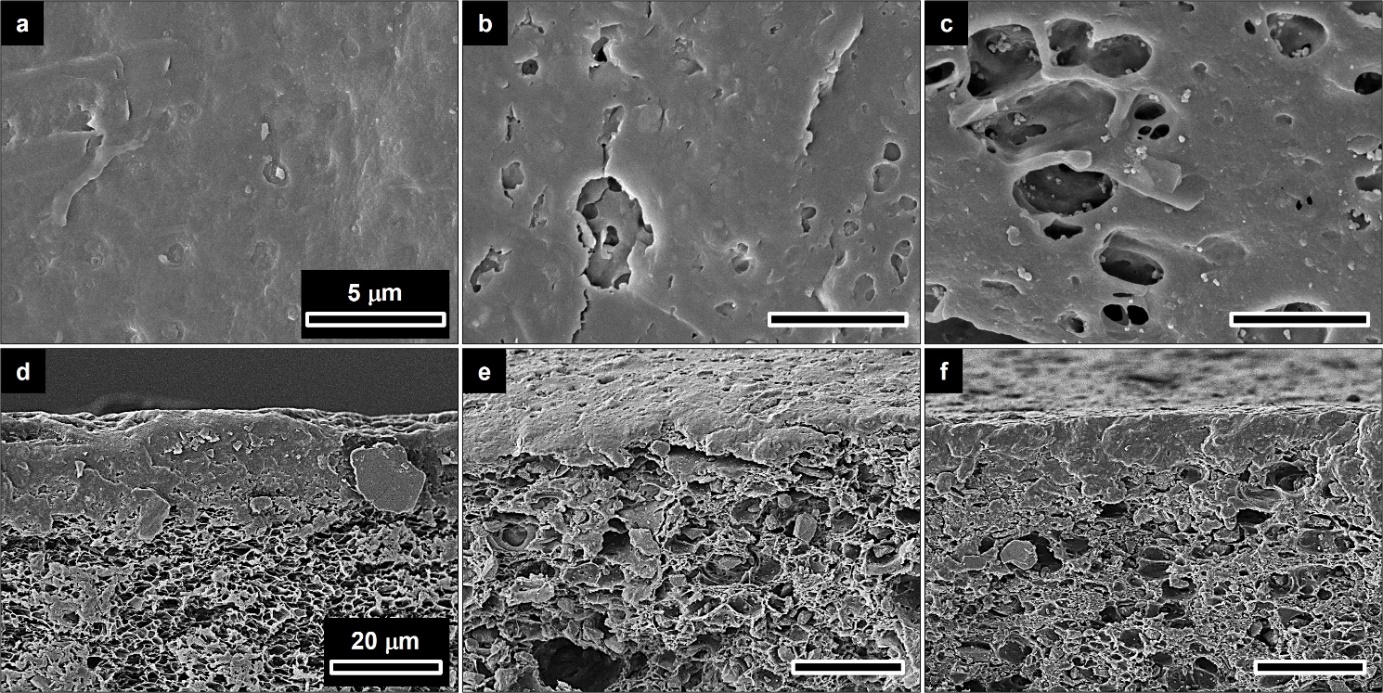


**Figure S44.** a−c) Surface and d−f) cross-sectional SEM images of the h-PVC plastics: h-PVC a,d) hose, b,e) wire sheath, c,f) and mold.


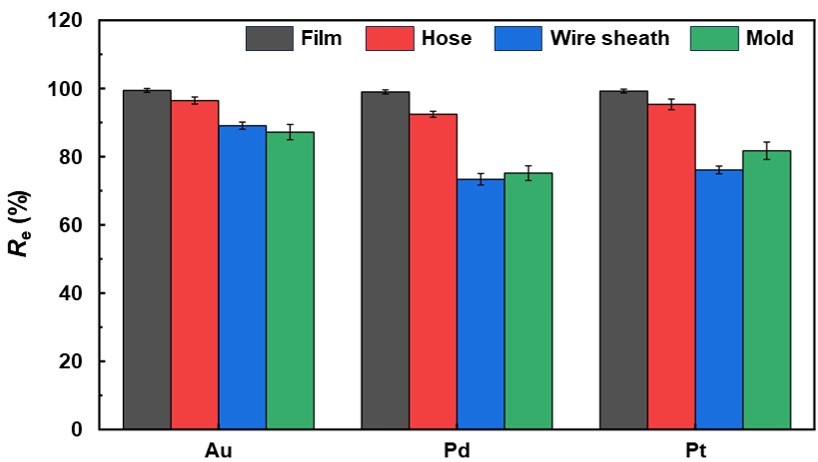


**Figure S45.** PM recovery efficiency (*R*_e_) of the h-PVC plastics (h-PVC plastic dose = 1.0 g L^−1^, *C*_i_ = 10 mg L^−1^, pH = 2, contact time = 12 h). A low *C*_i_ of 10 mg L^−1^ was used because it corresponds to the typical PM ion concentration of real-world leachates.^[3]^ Data represents the mean ± standard deviation (*n* = 3).


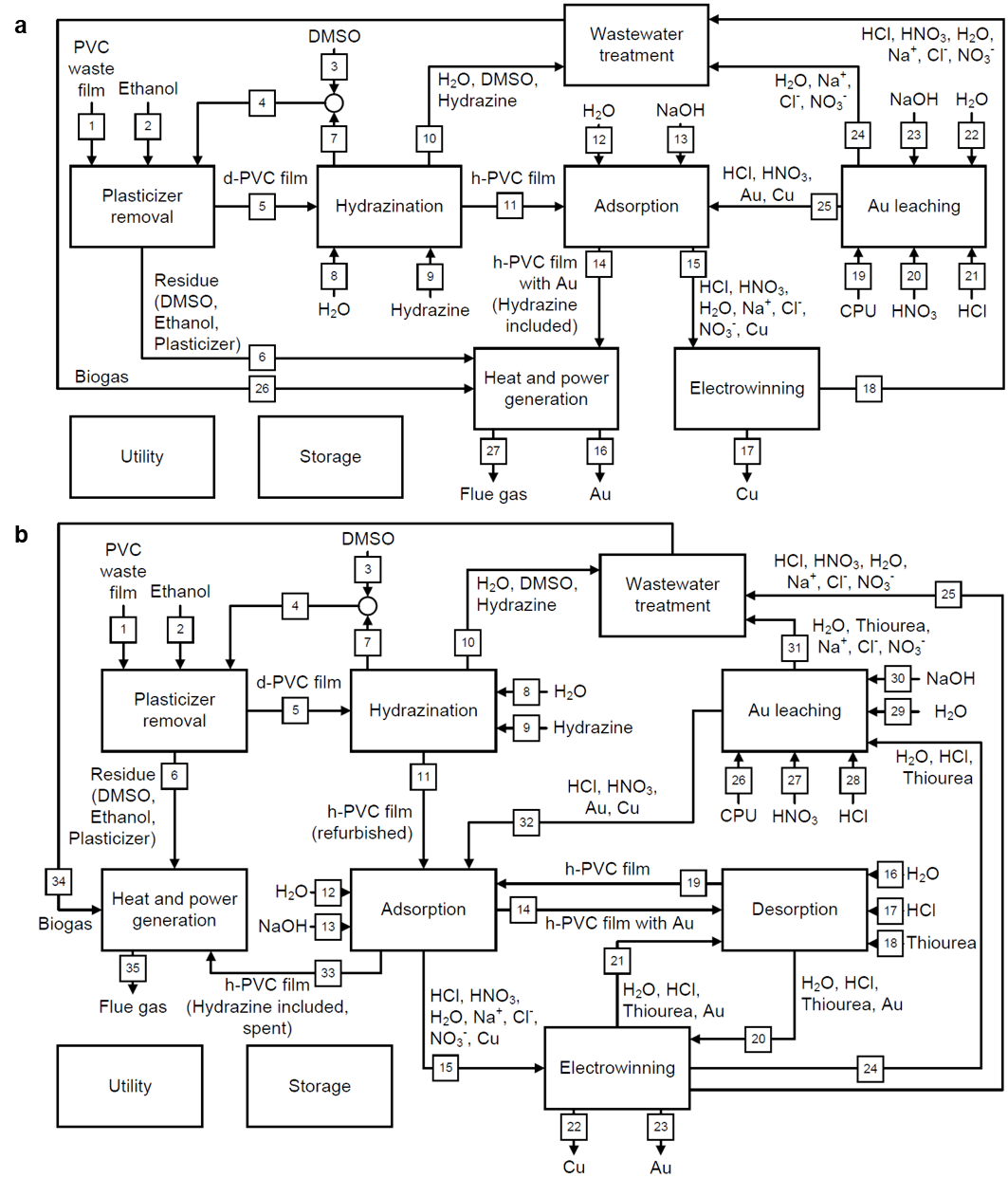


**Figure S46.** Block flow diagrams of the a) film calcination and b) film regeneration processes using the h-PVC plastic film for Au recovery from CPU waste. All the residues, including solvents (DMSO, ethanol), plasticizer, and hydrazine, generated during the formation of the h-PVC film are completely decomposed into monomers either in the heat and power generation system (combustion) or in the wastewater treatment system (digestion).

**
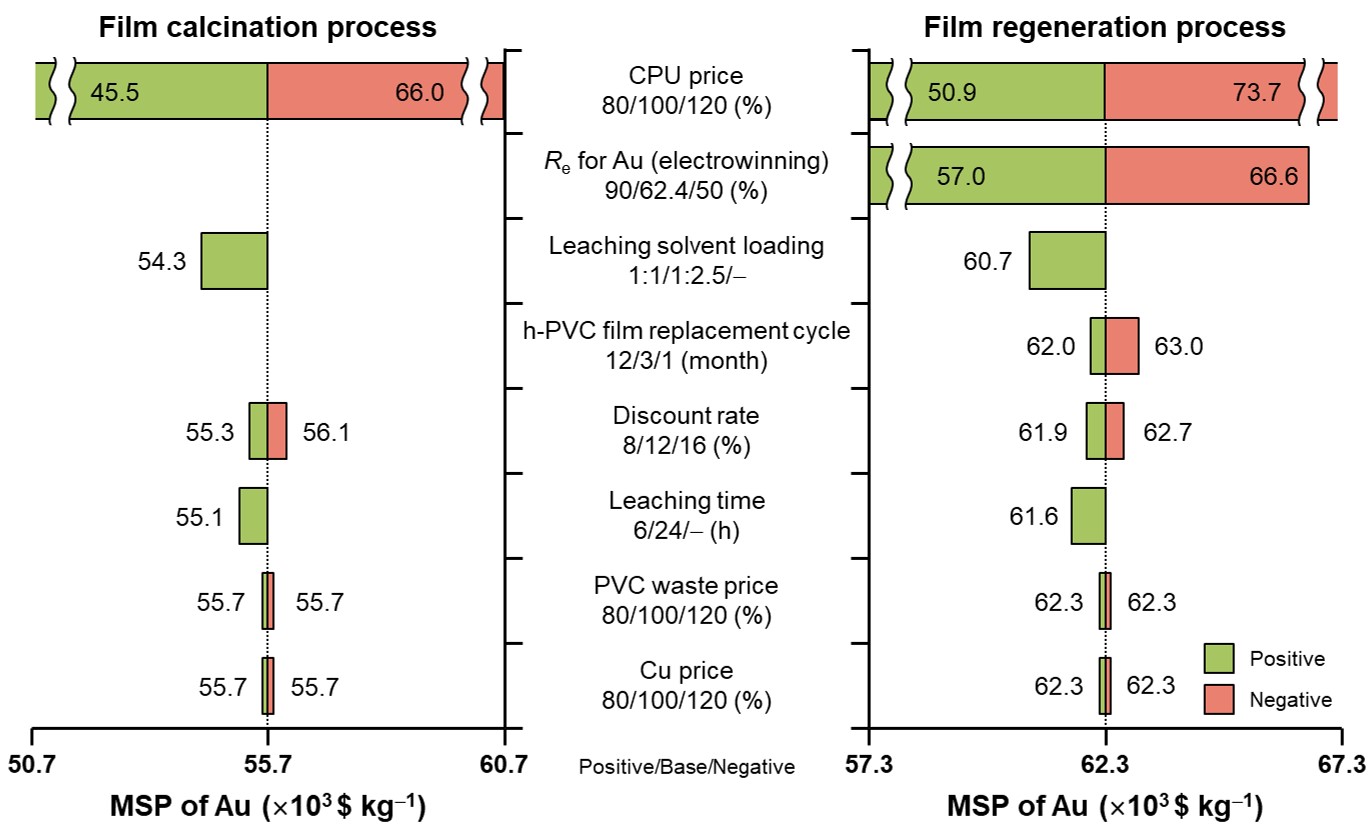
**

**Figure S47.** Sensitivity analysis of the film calcination and film regeneration processes.


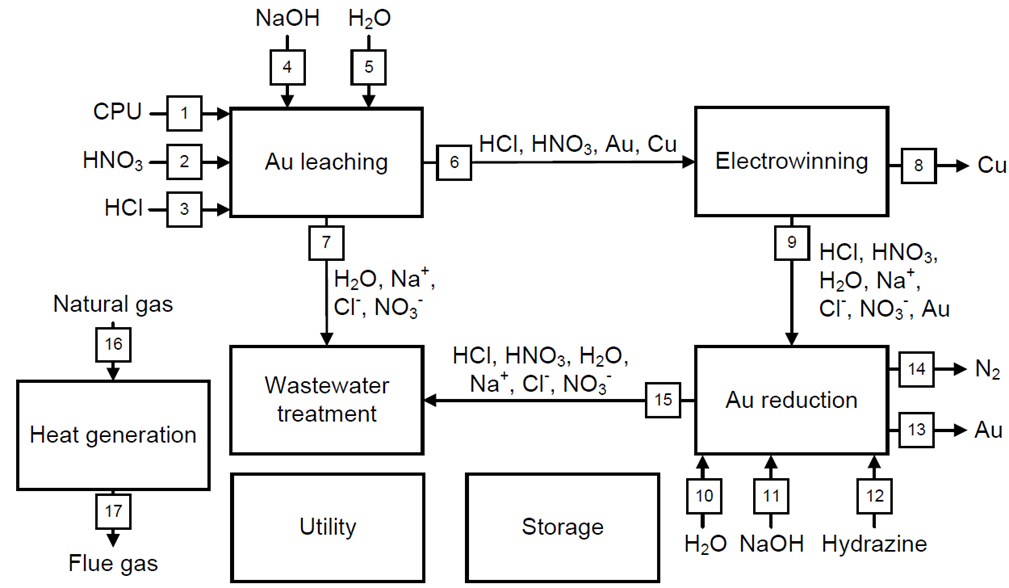


**Figure S48.** Block flow diagrams of the chemical reduction process using the hydrazine reducing agent for Au recovery from CPU waste. Hydrazine is added to the CPU leachate, followed by filtration to separate the resulting metallic precipitates and recover Au.


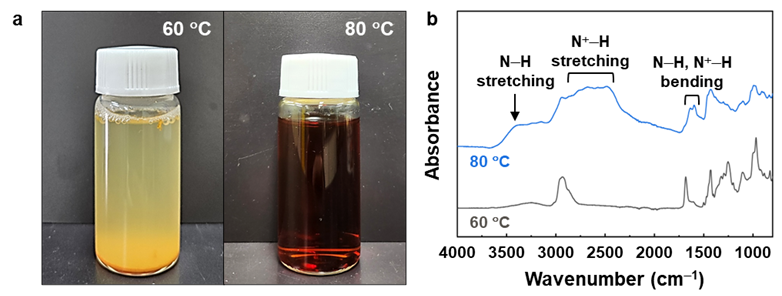


**Figure S****49.** a) Photographs of the corresponding aqueous solutions and b) FT-IR spectra of the h-PVC polymers hydrazinated at 60 and 80 °C for 5 d. Together with its water insoluble property, the significantly lower intensity of the hydrazine FT-IR peaks of the h-PVC polymer hydrazinated at 60 °C than that at 80 °C indicates a lower hydrazination efficiency (incomplete hydrazination) at 60 °C.

**Table S1.** Integrated area percentages of the deconvoluted N1s XPS peaks of the PM@h-PVC precipitates. The area percentage of the N=N peak decreased in the order of Au (21%) > Pt (16%) > Pd (14%), positively correlating with the reduction extent of these ions.

| Chemical bonding | Peak area percentage [%] | | |
| --- | --- | --- | --- |
|  | Au@h-PVC | Pd@h-PVC | Pt@h-PVC |
| −NH_3_^+^ | 21 | 18 | 19 |
| N−metal−N | 58 | 68 | 65 |
| N=N | 21 | 14 | 16 |
| Conversion^a)^ [%] | 50 | 44 | 46 |

^a)^Conversion of protonated hydrazine (−NH_3_^+^) groups to diazene groups (N=N).

**Table S2.** Kinetics model fitting parameters of the h-PVC polymer for three PMs.

|  | Pseudo-first-order | | | Pseudo-second-order | | |
| --- | --- | --- | --- | --- | --- | --- |
|  | *q*_e_  [mg g^−1^] | *k*_1_  [min^−1^] | *R*^2^ | *q*_e_  [mg g^−1^] | *k*_2_  [g mg^−1^ min^−1^] | *R*^2^ |
| Au | 1026 | 0.52 | 0.99 | 1036 | 2.56 | 0.99 |
| Pd | 656 | 0.30 | 0.98 | 683 | 0.95 | 0.99 |
| Pt | 637 | 0.13 | 0.94 | 688 | 0.30 | 0.98 |

**Table S3.** Isotherm model fitting parameters of the h-PVC polymer for three PMs.

|  | Langmuir | | | Freundlich | | |
| --- | --- | --- | --- | --- | --- | --- |
|  | *q*_m_  [mg g^−1^] | *K*_L_  [L mg^−1^] | *R*^2^ | *n* | *K*_F_  [mg g^−1^] | *R*^2^ |
| Au | 2304 | 1.97 | 0.95 | 5.24 | 893 | 0.77 |
| Pd | 759 | 0.10 | 0.98 | 3.34 | 149 | 0.84 |
| Pt | 803 | 0.15 | 0.96 | 3.49 | 186 | 0.91 |

**Table S4.** Maximum PM adsorption capacities (*q*_max_) and equilibrium times (*t*_eq_) of the h-PVC polymer and other reported PM adsorbents.

| Adsorbent | *q*_max_ [mg g^−1^]/*t*_eq_ [min] | | | Ref. |
| --- | --- | --- | --- | --- |
|  | Au | Pd | Pt |  |
| h-PVC | 2304/10 | 759/30 | 803/30 | This study |
| Star-shaped poly(acryloyl hydrazide) | 2847/1 | 1078/1 | 714/1 | [3] |
| Plant tannin-immobilized Fe_3_O_4_@SiO_2_ microsphere | 917/1440 | 196/1440 | − | [10] |
| Nanometer-sized titanium dioxide | 23/10 | 12/30 | − | [11] |
| Si-TpAL^a)^ | − | 48/300 | − | [12] |
| (3-Mercaptopropyl)trimethoxysilane-modified CoFe_2_O_4_ | 120/240 | − | − | [13] |
| MNP-G3^b)^ | 4/480 | 3/480 | − | [14] |
| AHPP-MOF^c)^ | − | 284/420 | − | [15] |
| Fe_3_O_4_ nanoparticle | − | 11/180 | 13/180 | [16] |
| Thiol-modified mesoporous silica | − | 10/300 | 18/480 | [17] |
| MPVA^d)^ | 1425/10 | − | − | [18] |
| Magnetic alginate polymer-imprinted diatomite composite | − | 60/140 | − | [19] |
| 1,4,7,10-Tetraazacyclododecane-modified mesoporous silica | − | 162/180 | − | [20] |
| Fe-BTC^e)^ | 934/2 | − | − | [21] |
| Zr(IV)-based metal-organic framework | 280/25 | 120/25 | 166/25 | [22] |
| Thiourea-modified zirconium-based MOF | 326/180 | − | − | [23] |
| MIL-101(Cr)-NH_2_^f)^ | − | 278/720 | 141/720 | [24] |
| UiO-66-BTU^g)^ | 658/300 | − | − | [25] |
| MOF-AFH^h)^ | 403/180 | 193/180 | − | [26] |
| UiO-66-NH_2_^i)^ | 650/1440 | − | − | [27] |
| Cyanide-activated cobalt hexacyanoferrate | − | − | 25/1440 | [28] |
| MIL-101(Cr)/ED-GA^j)^ | − | 323/120 | 416/60 | [29] |
| PCN-222-MBA^k)^ | 714/1 | − | − | [30] |
| IPMIL-101(Cr)^l)^ | − | 193/40 | − | [31] |
| PEPEI^m)^ | 743/1080 | 509/1080 | 572/1080 | [32] |
| Poly(allylamine hydrochloride)-modified *Escherichia coli* | − | − | 304/45 | [33] |
| Tannic acid-coated porous poly(EGDMA-co-AN)^n)^ microsphere | 52/60 | − | − | [34] |
| Chitosan-dibenzo-18-crown-6-ether | − | 19/180 | 23/180 | [35] |
| Poly-Cys-g-PDA@GPUF^o)^ | 1083/30 | 785/30 | 574/30 | [36] |
| Polyethyleneimine-grafted polyacrylonitrile capsule | 576/180 | − | − | [37] |
| Polyamine chelating resin | − | − | 162/1200 | [38] |
| Glycine-modified crosslinked chitosan resin | 170/120 | 120/120 | 122/180 | [39] |
| Dimethylaniline-modified paper | 906/300 | 224/300 | 176/300 | [40] |
| Human hair | 638/1440 | − | − | [41] |
| *N*-Aminoguanidine-modified persimmon tannin | 1753/240 | 214/120 | 197/1440 | [42] |
| 8-Hydroxyquinoline-2-carbaldehyde-grafted chitosan | − | 340/120 | 204/120 | [43] |
| Polyethyleneimine-incorporated algal bead | − | 136/1440 | 115/360 | [44] |
| Thiourea-modified chitosan microsphere | − | 42/150 | 57/300 | [45] |
| Thiourea-modified chitosan-imprinted resin | 933/240 | − | − | [46] |
| Poly(acryloyl hydrazide)-brushed carbon nanotube membrane | 292/180 | 187/180 | 267/180 | [47] |
| Graphene oxide | 108/360 | 81/120 | 71/40 | [48] |
| Mesoporous carbon | 492/1080 | 64/1080 | 78/1080 | [49] |
| Graphene oxide-TOABr^p)^ | − | 93/30 | − | [50] |
| B-N-WSBP biochar^q)^ | 461/180 | 134/180 | 47/180 | [51] |
| Graphene oxide/calcium alginate hydrogel | 82/1320 | − | − | [52] |
| Bismuth carbonate supported over carbon black | 13/60 | − | − | [53] |

^a)^3-Aminopropyl-functionalized silica-gel; ^b)^Magnetic nanoparticle modified by a third-generation dendrimer; ^c)^4-Amino-3-hydroxybenzoic acid-modified Zr-based metal-organic framework; ^d)^Ti_3_C_2_ MXene-polyhedral oligomeric silsesquioxane aerogel with uniformly dispersed calcined-V_2_O_3_; ^e)^Fe_3_O clusters interlinked by 1,3,5-benzenetricarboxylate ligands; ^f)^Cr-based metal-organic framework; ^g)^Zr-based metal-organic framework functionalized by bisthiourea; ^h)^2,6-Aminopyridine-grafted 3-formyl-4-hydroxybenzoic acid-based metal-organic framework; ^i)^Zr-based metal-organic framework crosslinked by 2-aminoterephthalic acid; ^j)^Cr-based metal-organic framework functionalized with ethylenediamine and glutaraldehyde; ^k)^Porous coordination network with 4-mercaptobenzoic acid; ^l)^Pd^2+^ ion-imprinted Cr-based metal-organic framework; ^m)^*N*-2-(2-Pyridyl)-ethyl polyethyleneimine; ^n)^Poly(ethylene glycol dimethacrylate-co-acrylonitrile); ^o)^Cysteine polymer brush-grafted polydopamine-modified graphene-based polyurethane foam; ^p)^Tetraoctylammonium bromide-impregnated graphene oxide; ^q)^Boron–nitrogen co-doped walnut shell biochar powder

**Table S5.** Integrated area percentages of the PM(0) and PM ion peaks of the PM@h-PVC polymer precipitates and PM@h-PVC films.

|  | XPS peak area percentage [%] | | | | | |
| --- | --- | --- | --- | --- | --- | --- |
|  | Au@h-PVC precipitate | Pd@h-PVC precipitate | Pt@h-PVC precipitate | Au@h-PVC film | Pd@h-PVC film | Pd@h-PVC film |
| PM(0) | 89 | 29 | 80 | 87 | 21 | 68 |
| PM ion | 11 | 71 | 20 | 13 | 79 | 32 |

**Table S6.** Isotherm model fitting parameters of the h-PVC film for three PMs.

|  | Langmuir | | | Freundlich | | |
| --- | --- | --- | --- | --- | --- | --- |
|  | *q*_m_  [mg g^−1^] | *K*_L_  [L mg^−1^] | *R*^2^ | *n* | *K*_F_  [mg g^−1^] | *R*^2^ |
| Au | 355 | 3.37 | 0.98 | 2.42 | 34 | 0.92 |
| Pd | 141 | 5.34 | 0.99 | 3.36 | 24 | 0.84 |
| Pt | 241 | 3.43 | 0.98 | 2.68 | 28 | 0.88 |

**Table S7** Maximum PM adsorption capacities (*q*_max_) and equilibrium times (*t*_eq_) of the h-PVC film and other reported macroscale PM adsorbents.

| Adsorbent | *q*_max_ [mg g^−1^]/*t*_eq_ [h] | | | Ref. |
| --- | --- | --- | --- | --- |
|  | Au | Pd | Pt |  |
| h-PVC film | 355/12 | 141/12 | 241/12 | This study |
| Phosphonic acid-functionalized poly(vinyl chloride) | 336/18 | − | − | [8] |
| Porous amine-functionalized poly(acrylonitrile) capsule | 576/3 | − | − | [37] |
| Glutaraldehyde-crosslinked chitosan grafted with 8-hydroxyquinoline-2-carbaldehyde | − | 340/1 | 204/1 | [43] |
| Sericin-alginate beads crosslinked by polyethylene glycol diglycidyl ether | − | 16/30 | − | [54] |
| Glutaraldehyde-crosslinked chitosan beads | 882/12 | − | − | [55] |
| *N*-(2-Bis[2-aminoethyl)amino]-ethyl)aminomethyl-polystyrene polymer beads | 168/12 | 205/12 | − | [56] |
| UiO-66-NH_2_^a)^ | − | 335/24 | − | [57] |
| Bamboo shoot shell biochar microspheres | − | 187/6 | − | [58] |
| Dialdehyde carboxymethyl cellulose-crosslinked chitosan | − | 89/1 | 81/0.5 | [59] |
| Calcined wheat bran | − | − | 27/16 | [60] |
| Chitosan-assembled diatomaceous earth-sugarcane bagasse | − | − | 216/10 | [61] |
| Thiourea-functionalized polyacrylonitrile beads | − | 30/6 | 49/6 | [62] |
| Glycine-modified crosslinked chitosan resin | 170/3 | 120/3 | 123/3 | [63] |
| Tannin from Indian almond leaf biomass | − | 28/3 | 47/3 | [64] |
| Bayberry tannin-immobilized collagen fiber membrane | − | 33/4 | 46/4 | [65] |
| Polyethylenimine-crosslinked poly(vinyl chloride) | − | − | 217/1.5 | [66] |
| Hydroxyapatite-assembled silk nanofibril membrane | 164/12 | − | − | [67] |
| L-Lysine-modified crosslinked chitosan resin | 70/10 | 110/10 | 129/10 | [68] |
| TEMPO^b)^-oxidized cellulose nanofiber film | 15/24 | − | − | [69] |

^a)^Zr-based metal-organic framework crosslinked by 2-aminoterephthalic acid; ^b)^(2,2,6,6-Tetramethylpiperidin-1-yl)oxidanyl

**Table S8.** Capital costs (×10^6^ $) of the film calcination, film regeneration, and chemical reduction processes.

| Subsystem | Film calcination | Film regeneration | Chemical reduction |
| --- | --- | --- | --- |
| Plasticizer removal | 16.1 | 16.1 | − |
| Hydrazination | 2.5 | 2.3 | − |
| Au leaching | 94.6 | 94.6 | 94.8 |
| Adsorption | 2.2 | 2.1 | − |
| Chemical reduction | − | − | 1.7 |
| Desorption | − | 0.8 | − |
| Electrowinning | 2.6 | 6.4 | 2.6 |
| Wastewater treatment | 57.2 | 59.8 | 57.3 |
| Heat and power generation | 19.3 | 10.8 | 4.3 |
| Storage | 3.3 | 3.3 | 3.3 |
| Utility | 4.8 | 4.0 | 3.4 |
| Total installed equipment cost | 109.2 | 107.4 | 89.8 |
| Total capital investment | 202.6 | 202.2 | 167.4 |

Capital cost was calculated using economic parameters (e.g., installation factors and discount rate) based on an *n*th plant assumption as follows:

$$\text{Capital cost}\text{ }\text{}\text{ }\text{Installed equipment cost}\text{ }\text{}\text{ }\text{Total direct cost}\text{ }\text{}\text{ }\text{Total indirect cost}$$

$$\text{}\text{ }\text{Fixed capital investment}\text{ }\text{}\text{ }\text{Total capital investment}$$

**Table S9.** Operating costs (×10^6^ $ year^−1^) of the film calcination, film regeneration, and chemical reduction processes.

| Subsystem | Film calcination | Film regeneration | Chemical reduction |
| --- | --- | --- | --- |
| Plasticizer removal | 12.1 | 5.4 | − |
| Hydrazination | 1.5 | 0.7 | − |
| Au leaching | 1003.6 | 1003.8 | 1004.2 |
| Adsorption | 21.5 | 21.5 | − |
| Chemical reduction | − | − | 22.3 |
| Desorption | − | 10.3 | − |
| Electrowinning | 0.1 | 0.3 | 0.1 |
| Wastewater treatment | 2.5 | 2.7 | 2.6 |
| Heat and power generation | 1.0 | 0.5 | 0.3 |
| Storage | 0.1 | 0.1 | 0.2 |
| Utility | 0.2 | 0.2 | 0.2 |
| Fixed cost | 9.4 | 9.4 | 8.9 |
| Total operating cost | 1042.6 | 1045.5 | 1029.9 |

The operating cost of each subsystem includes raw material (e.g., PVC and CPU waste), cooling utility (i.e., cooling water), and fixed (e.g., salaries and property insurance) costs

**Table S10.** Minimum selling price (MSP, ×10^3^ $ kg^−1^) and purity of recovered Au.

| Process | MSP | Purity [%] |
| --- | --- | --- |
| Calcination | 55.7 | 98 |
| Regeneration | 62.3 | 98 |
| Chemical reduction | 56.7 | 77 |

**Table S11.** Environmental impacts of the film calcination, film regeneration, chemical reduction, and conventional Au production processes.

| Impact category | Process | | | |
| --- | --- | --- | --- | --- |
|  | Film  calcination | Film  regeneration | Chemical  reduction | Conventional Au production |
| Global warming potential | 95% | 106% | 95% | 100% |
| Ionizing radiation | 68% | 76% | 67% | 100% |
| Ozone formation, human health | 20% | 23% | 21% | 100% |
| Fine particulate matter formation | 50% | 57% | 50% | 100% |
| Ozone formation, terrestrial ecosystem | 20% | 23% | 21% | 100% |
| Terrestrial acidification | 49% | 56% | 50% | 100% |
| Freshwater eutrophication | 0% | 0% | 0% | 100% |
| Terrestrial ecotoxicity | 21% | 13% | 14% | 100% |
| Freshwater ecotoxicity | 0% | 0% | 0% | 100% |
| Marine ecotoxicity | 0% | 0% | 0% | 100% |
| Human carcinogenic toxicity | 1% | 2% | 2% | 100% |
| Human noncarcinogenic toxicity | 0% | 0% | 0% | 100% |
| Land use | 8% | 9% | 8% | 100% |
| Mineral resource scarcity | 1% | 1% | 1% | 100% |
| Fossil resource scarcity | 65% | 75% | 64% | 100% |

**Table S12.** Economic parameters and assumptions for TEA analysis^a)^.

| Input | Price | Ref. |
| --- | --- | --- |
| PVC waste film | 434.8 $ ton^−1^ | [70] |
| DMSO | 3000 $ ton^−1^ | [71] |
| EtOH | 770 $ ton^−1^ | [72] |
| H_2_O price | 0.4 $ ton^−1^ | [72] |
| NaOH | 525.6 $ ton^−1^ | [72] |
| Boiler chemicals | 6563.6 $ ton^−1^ | [72] |
| Flue gas desulfurization lime | 262.1 $ ton^−1^ | [72] |
| Ammonia | 418.9 $ ton^−1^ | [72] |
| Cooling tower chemicals | 3933.5 $ ton^−1^ | [72] |
| Hydrazine | 2645 $ ton^−1^ | [73] |
| Waste CPU | 30000 $ ton^−1^ | [74] |
| HCl | 168 $ ton^−1^ | [75] |
| HNO_3_ | 300 $ ton^−1^ | [76] |
| Electricity | 8.1 cents kWh^−1^ | [77] |
| Cu | 8804.0 $ ton^−1^ | [78] |
| Economic parameters | | |
| Internal rate of return [%] | 12.0 | |
| Tax rate [%] | 30.0 | |
| Depreciation period for general plant [years] | 20.0 | |
| Depreciation period for steam/electricity system [years] | 7.0 | |

^a)^Assumptions: 1) The capital investment is spread over 3 years at rates of 8%, 60%, and 32% in the first, second, and third years, respectively. 2) The working capital is 5% of the fixed capital investment. 3) The capital charge factor, calculated by discounted cash flow analysis, is 0.154 for both film calcination and film regeneration processes.

**Table S13.** Energy requirements before and after heat integration.

| Process | Energy required | Before  [MW] | After  [MW] | Decrease  [%] |
| --- | --- | --- | --- | --- |
| Calcination | Heating | 1.6 | 0.6 | 62.5 |
|  | Cooling | 22.9 | 21.9 | 4.4 |
| Regeneration | Heating | 0.6 | 0.2 | 66.7 |
|  | Cooling | 16.8 | 16.4 | 2.4 |

References

[1] J. Wang, X. Guo, Adsorption kinetic models: Physical meanings, applications, and solving methods. *J. Hazard. Mater.* **2020**, 390, 122156.

[2] M. A. Al-Ghouti, D. A. Da'ana, Guidelines for the use and interpretation of adsorption isotherm models: A review. *J. Hazard. Mater.* **2020**, 393, 122383.

[3] S. S. Shin, Y. Jung, S. Jeon, S.-J. Park, S.-J. Yoon, K.-W. Jung, J.-W. Choi, J.-H. Lee, Efficient recovery and recycling/upcycling of precious metals using hydrazide-functionalized star-shaped polymers. *Nat. Commun.* **2024**, 15, 3889.

[4] C. Nan, J. Dong, H. Tian, H. Shi, S. Shen, J. Xu, X. Li, T. Shi, Oxidations of hydrazine and substituted hydrazines by hexachloroiridate (IV) in aqueous solution: Kinetic and mechanistic analyses. *J. Mol. Liq.* **2018**, 256, 489.

[5] M. Gurung, B. B. Adhikari, S. Alam, H. Kawakita, K. Ohto, K. Inoue, Persimmon tannin-based new sorption material for resource recycling and recovery of precious metals. *Chem. Eng. J.* **2013**, 228, 405.

[6] J. Park, S. W. Won, J. Mao, I. S. Kwak, Y.-S. Yun, Recovery of Pd(II) from hydrochloric solution using polyallylamine hydrochloride-modified Escherichia coli biomass. *J. Hazard. Mater.* **2010**, 181, 794.

[7] H. Wu, Y. Wang, L. O. Jones, W. Liu, L. Zhang, B. Song, X.-Y. Chen, C. L. Stern, G. C. Schatz, J. F. Stoddart, Selective separation of hexachloroplatinate (IV) dianions based on exo‐binding with cucurbit [6] uril. *Angew. Chem. Inter. Ed.* **2021**, 60, 17587.

[8] J. Feng, D. Xu, Z. Tang, H. Chen, C. He, S. Ning, M. Li, G. Yuan, A novel phosphonic acid functionalized poly(vinyl chloride) electrospun nanofiber for efficient adsorption of gold from aqueous solution. *Polymer* **2023**, 274, 125899.

[9] L. Zhou, X. Lu, Z. Ju, B. Liu, H. Yao, J. Xu, Q. Zhou, Y. Hu, S. Zhang, Alcoholysis of polyethylene terephthalate to produce dioctyl terephthalate using choline chloride-based deep eutectic solvents as efficient catalysts. *Green Chem.* **2019**, 21, 897.

[10] R. Fan, H. Min, X. Hong, Q. Yi, W. Liu, Q. Zhang, Z. Luo, Plant tannin immobilized Fe_3_O_4_@SiO_2_ microspheres: A novel and green magnetic bio-sorbent with superior adsorption capacities for gold and palladium. *J. Hazard. Mater.* **2019**, 364, 780.

[11] Y. Qing, Y. Hang, R. Wanjaul, Z. Jiang, B. Hu, Adsorption behavior of noble metal ions (Au, Ag, Pd) on nanometer-size titanium dioxide with ICP-AES. *Anal. Sci.* **2003**, 19, 1417.

[12] H. Wu, S.-Y. Kim, T. Ito, M. Miwa, S. Matsuyama, One-pot synthesis of silica-gel-based adsorbent with Schiff base group for the recovery of palladium ions from simulated high-level liquid waste. *Nucl. Eng. Technol.* **2022**, 54, 3641.

[13] A. Kraus, K. Jainae, F. Unob, N. Sukpirom, Synthesis of MPTS-modified cobalt ferrite nanoparticles and their adsorption properties in relation to Au(III). *J. Colloid Interface Sci.* **2009**, 338, 359.

[14] C.-H. Yen, H.-L. Lien, J.-S. Chung, H.-D. Yeh, Adsorption of precious metals in water by dendrimer modifiedmagnetic nanoparticles. *J. Hazard. Mater.* **2017**, 322, 215.

[15] J. Tang, J. Zhao, S. Wang, L. Zhang, M. Zhao, Z. Huang, Y. Hu, Pre-modification strategy to prepare a novel Zr-based MOF for selective adsorption of Palladium(II) from solution. *Chem. Eng. J.* **2021**, 407, 127223.

[16] A. Uheida, M. Iglesias, C. Fontàs, Y. Zhang, Adsorption behavior of platinum group metals (Pd, Pt, Rh) on nonylthiourea‐coated Fe_3_O_4_ nanoparticles. *Sep. Sci. Technol.* **2006**, 41, 909.

[17] T. Kang, Y. Park, J. Yi, Highly selective adsorption of Pt^2+^ and Pd^2+^ using thiol-functionalized mesoporous silica. *Ind. Eng. Chem. Res.* **2004**, 43, 1478.

[18] W. Wang, X. Li, R. Wang, J. Wang, H. Deng, S. Tong, Three-dimensional Ti_3_C_2_ MXene-POSS/V_2_O_3_@C nanocomposite aerogel for ultrafast and selective recovery of gold (III) at low temperatures. *Chem. Eng. J.* **2023**, 468, 143453.

[19] H. Rasoulzadeh, A. Sheikhmohammadi, M. Abtahi, B. Roshan, R. Jokar, Eco-friendly rapid removal of palladium from aqueous solutions using alginate-diatomite magnano composite. *J. Environ. Chem. Eng.* **2021**, 9, 105954.

[20] C. Zeng, P. Liu, Z. Xiao, Y. Li, L. Song, Z. Cao, D. Wu, Y.-F. Zhang, Highly selective adsorption and recovery of palladium from spent catalyst wastewater by 1,4,7,10-tetraazacyclododecane-modified mesoporous silica. *ACS Sustainable Chem. Eng.* **2022**, 10, 1103.

[21] D. T. Sun, N. Gasilova, S. Yang, E. Oveisi, W. L. Queen, Rapid, selective extraction of trace amounts of gold from complex water mixtures with a metal-organic framework (MOF)/polymer composite. *J. Am. Chem. Soc.* **2018**, 140, 16697.

[22] S. Lin, D. H. K. Reddy, J. K. Bediako, M.-H. Song, W. Wei, J.-A. Kim, Y.-S. Yun, Effective adsorption of Pd(II), Pt(IV) and Au(III) by Zr(IV)-based metal-organic frameworks from strongly acidic solutions. *J. Mater. Chem. A* **2017**, 5, 13557.

[23] C. Wu, X. Zhu, Z. Wang, J. Yang, Y. Li, J. Gu, Specific recovery and in situ reduction of precious metals from waste to create MOF composites with immobilized nanoclusters. *Ind. Eng. Chem. Res.* **2017**, 56, 13975.

[24] C.-R. Lim, S. Lin, Y.-S. Yun, Highly efficient and acid-resistant metal-organic frameworks of MIL-101(Cr)-NH_2_ for Pd(II) and Pt(IV) recovery from acidic solutions: Adsorption experiments, spectroscopic analyses, and theoretical computations. *J. Hazard. Mater.* **2020**, 387, 121689.

[25] J. Guo, X. Fan, J. Wang, S. Yu, M. Laipan, X. Ren, C. Zhang, L. Zhang, Y. Li, Highly efficient and selective recovery of Au(III) from aqueous solution by bisthiourea immobilized UiO-66-NH_2_: Performance and mechanisms. *Chem. Eng. J.* **2021**, 425, 130588.

[26] J. Tang, Y. Chen, S. Wang, D. Kong, L. Zhang, Highly efficient metal-organic frameworks adsorbent for Pd(II) and Au(III) recovery from solutions: Experiment and mechanism. *Environ. Res.* **2022**, 210, 112870.

[27] Z. Chang, F. Li, X. Qi, B. Jiang, J. Kou, Selective and efficient adsorption of Au(III) in aqueous solution by Zr-based metal-organic frameworks (MOFs): An unconventional way for gold recycling. *J. Hazard. Mater.* **2020**, 390, 122175.

[28] M. Liu, X. Long, X. Li, Y. Du, Y. Zhao, Z. Huang, Y. Chen, R. Chen, Enrichment and activation of cyano in cobalt hexacyanoferrate for specific recovery of ultra-low concentrations of platinum. *Sep. Purif. Technol.* **2024**, 328, 124925.

[29] T. C. Maponya, K. Makgopa, T. R. Somo, D. M. Tshwane, K. D. Modibane, Highly adsorptive removal of palladium and platinum ions from wastewater using novel ethylenediamine-glutaraldehyde-grafted metal organic framework. *Environ. Nanotechnol. Monit. Manage.* **2023**, 20, 100805.

[30] S. Nazri, M. Khajeh, A. R. Oveisi, R. Luque, E. Rodríguez-Castellón, M. Ghaffari-Moghaddam, Thiol-functionalized PCN-222 MOF for fast and selective extraction of gold ions from aqueous media. *Sep. Purif. Technol.* **2021**, 259, 118197.

[31] T. C. Maponya, K. D. Modibane, T. R. Somo, K. Makgopa, Selective adsorption of palladium ions from wastewater by ion-imprinted MIL-101 (Cr) derived from waste polyethylene terephthalate: Isotherms and kinetics. *Sep. Purif. Technol.* **2023**, 307, 122767.

[32] S. Bratskaya, Y. Privar, A. Ustinov, Y. Azarova, A. Pestov, Recovery of Au(III), Pt(IV), and Pd(II) using pyridylethyl-containing polymers: Chitosan derivatives vs synthetic polymers. *Ind. Eng. Chem. Res.* **2016**, 55, 10377.

[33] J. Mao, S. Y. Lee, S. W. Won, Y.-S. Yun, Surface modified bacterial biosorbent with poly(allylamine hydrochloride): Development using response surface methodology and use for recovery of hexachloroplatinate (IV) from aqueous solution. *Water Res.* **2010**, 44, 5919.

[34] J. Kim, K. R. Kim, Y. Hong, S. Choi, C. T. Yavuz, J. W. Kim, Y. S. Nam, Photochemically enhanced selective adsorption of gold ions on tannin-coated porous polymer microspheres. *ACS Appl. Mater. Interfaces* **2019**, 11, 21915.

[35] O. Grad, M. Ciopec, A. Negrea, N. Duțeanu, G. Vlase, P. Negrea, C. Dumitrescu, T. Vlase, R. Vodă, Precious metals recovery from aqueous solutions using a new adsorbent material. *Sci. Rep.* **2011**, 11, 2016.

[36] D. Xue, T. Li, Y. Liu, Y. Yang, Y. Zhang, J. Cui, D. Guo, Selective adsorption and recovery of precious metal ions from water and metallurgical slag by polymer brush graphene–polyurethane composite. *React. Funct. Polym.* **2019**, 136, 138.

[37] Y. Jung, T. Do, U. S. Choi, K.-W. Jung, J.-W. Choi, Cage-like amine-rich polymeric capsule with internal 3D center-radial channels for efficient and selective gold recovery. *Chem. Eng. J.* **2022**, 438, 135618.

[38] B. Li, W. Xiong, Y. Cao, X. Zhou, H. Zhu, M. Li, L. Yang, P. Shao, Targeting of platinum capture under 1+1 aqua regia using robust and recyclable polymeric polyamine resin: Adsorption performance and mechanism. *Environ. Res.* **2023**, 227, 115814.

[39] A. Ramesh, H. Hasegawa, W. Sugimoto, T. Maki, K. Udea, Adsorption of gold(III), platinum(IV) and palladium(II) onto glycine modified crosslinked chitosan resin. *Bioresour. Technol.* **2008**, 99, 3801.

[40] C. R. Adhikari, D. Parajuli, H. Kawakita, K. Inoue, K. Ohto, H. Harada, Dimethylamine-modified waste paper for the recovery of precious metals. *Environ. Sci. Technol.* **2008**, 42, 5486.

[41] D. Yu, S. Morisada, H. Kawakita, K. Sakaguchi, S. Osada, K. Ohto, K. Inoue, S.-M. Song, G. Zhang, R. R. Sathuluri, Gold recovery from precious metals in acidic media by using human hair waste as a new pretreatment-free green material. *J. Environ. Chem. Eng.* **2021**, 9, 104724.

[42] M. Gurung, B. B. Adhikari, S. Morisado, H. Kawakita, K. Ohto, K. Inoue, S. Alam, *N*-aminoguanidine modified persimmon tannin: A new sustainable material for selective adsorption, preconcentration and recovery of precious metals from acidic chloride solution. *Bioresour. Technol.* **2013**, 129, 108.

[43] S. Mincke, T. G. Asere, I. Verheye, K. Folens, F. V. Bussche, L. Lapeire, K. Verbeken, P. V. D. Voort, D. A. Tessema, F. Fufa, G. D. Laing, C. V. Stevens, Functionalized chitosan adsorbents allow recovery of palladium and platinum from acidic aqueous solutions. *Green Chem.* **2019**, 21, 2295.

[44] S. Wang, T. Vincent, J.-C. Roux, C. Faur, E. Guibal, Pd(II) and Pt(IV) sorption using alginate and algal-based beads. *Chem. Eng. J.* **2017**, 313, 567.

[45] L. Zhou, J. Liu, Z. Liu, Adsorption of platinum(IV) and palladium(II) from aqueous solution by thiourea-modified chitosan microspheres. *J. Hazard. Mater.* **2009**, 172, 439.

[46] J. Guo, X. Fan, Y. Li, S. Yu, Y. Zhang, L. Wang, X. Ren, Mechanism of selective gold adsorption on ion-imprinted chitosan resin modified by thiourea. *J. Hazard. Mater.* **2021**, 415, 125617.

[47] L. Zhang, X. Zha, G. Zhang, J. Gu, W. Zhang, Y. Huang, J. Zhang, T. Chen, Designing a reductive hybrid membrane to selectively capture noble metallic ions during oil/water emulsion separation with further function enhancement. *J. Mater. Chem. A* **2018**, 6, 10217.

[48] L. Liu, S. Liu, Q. Zhang, C. Li, C. Bao, X. Liu, P. Xiao, Adsorption of Au(III), Pd(II), and Pt(IV) from aqueous solution onto graphene oxide. *J. Chem. Eng. Data* **2013**, 58, 209.

[49] P. R. Zalupski, R. McDowell, G. Dutech, The adsorption of gold, palladium, and platinum from acidic chloride solutions on mesoporous carbons. *Solvent Extr. Ion Exch.* **2014**, 32, 737−748.

[50] S. Sharma, N. Rajesh, Synergistic influence of graphene oxide and tetraoctylammonium bromide (frozen ionic liquid) for the enhanced adsorption and recovery of palladium from an industrial catalyst. *J. Environ. Chem. Eng.* **2016**, 4, 4287.

[51] Z. Wang, X. Xu, S. Ma, H. Wang, H. Zhao, Y. Wang, S. Tong, Z. Su, W. Wang, J. Bai, The superior adsorption capacity of boron-nitrogen co-doping walnut shell biochar powder for Au(III), Pt(IV), and Pd(II). *J. Environ. Chem. Eng.* **2021**, 9, 106288.

[52] S. Saha, M. Venkatesh, H. Basu, M. V. Pimple, R. K. Singhal, Recovery of gold using graphene oxide/calcium alginate hydrogel beads from a scrap solid state detector. *J. Environ. Chem. Eng.* **2019**, 7, 103134.

[53] C. Ianăşi, P. Svera, A. Popa, R. Lazău, A. Negrea, P. Negrea, N. Duteanu, M. Ciopec, N.-S. Nemes, Adsorbent material based on carbon black and bismuth with tunable properties for gold recovery. *Materials* **2023**, 16, 2837.

[54] T. L. da Silva, M. G. C. da Silva, M. G. A. Vieira, Palladium adsorption on natural polymeric sericin-alginate particles crosslinked by polyethylene glycol diglycidyl ether. *J. Environ. Chem. Eng.* **2021**, 9, 105617.

[55] T. H. Bui, W. Lee, S. B. Jeon, K. W. Kim, Y. Lee, Enhanced Gold (III) adsorption using glutaraldehyde-crosslinked chitosan beads: Effect of crosslinking degree on adsorption selectivity, capacity, and mechanism. *Sep. Purif. Technol.* **2020**, 248, 116989.

[56] M. Sayın, M. Can, M. Imamoglu, Adsorption of Pd(II) and Au(III) ions by commercial tris (2-aminoethyl) amine polystyrene polymer beads. *J. Chem. Eng. Data* **2021**, 66, 1132.

[57] J. Feng, M. Xie, D. Xu, Z. Tang, C. He, S. Ning, M. Lin, G. Yuan, S. Jiang, Preparation of metal-organic framework composite beads for selective adsorption and separation of palladium: Properties, mechanism and practical application. *Sep. Purif. Technol.* **2022**, 302, 122081.

[58] J. Yang, Q. Long, Y. Zhu, C. Lin, X. Xu, B. Pan, W. Shi, Y. Guo, J. Deng, Q. Yao, Z. Wang, Multifunctional self-assembled adsorption microspheres based on waste bamboo shoot shells for multi-pollutant water purification. *Environ. Res.* **2024**, 249, 118452.

[59] T. G. Asere, S. Mincke, K. Folens, F. V. Bussche, L. Lapeire, K. Verbeken, P. V. D. Voort, D. A. Tessema, G. D. Laing, C. V. Stevens, Dialdehyde carboxymethyl cellulose cross-linked chitosan for the recovery of palladium and platinum from aqueous solution. *React. Funct. Polym*. **2019**, 141, 145.

[60] F. Ogata, Y. Uematsu, N. Nagai, M. Nakamura, A. Tabuchi, C. Saenjum, T. Nakamura, N. Kawasaki, Wheat brans as waste biomass based on a potential bio–adsorbent for removing platinum (IV) ions from aqueous phase. *Bioresour. Technol. Rep.* **2022**, 20, 101238.

[61] J. Yang, T. Liang, B. Pan, X. Xu, Y. Guo, W. Shi, Z. Wang, A spherical adsorbent produced from a bagasse biochar chitosan assembly for selective adsorption of platinum-group metals from wastewater. *Int. J. Biol. Macromol.* **2024**, 266, 131142.

[62] M. H. Morcali, L. T. Khajavi, D. B. Dreisinger, Extraction of nickel and cobalt from nickeliferous limonitic laterite ore using borax containing slags. *Int. J. Miner. Process.* **2017**, 167, 27.

[63] A. Ramesh, H. Hasegawa, W. Sugimoto, T. Maki, K. Ueda, Adsorption of gold (III), platinum (IV) and palladium (II) onto glycine modified crosslinked chitosan resin. *Bioresour. Technol.* **2008**, 99, 3801.

[64] P. Ramakul, Y. Yanachawakul, N. Leepipatpiboon, N. Sunsandee, Biosorption of palladium (II) and platinum (IV) from aqueous solution using tannin from Indian almond (Terminalia catappa L.) leaf biomass: Kinetic and equilibrium studies. *Chem. Eng. J.* **2012**, 193, 102.

[65] H. W. Ma, X. P. Liao, X. Liu, B. Shi, Recovery of platinum (IV) and palladium (II) by bayberry tannin immobilized collagen fiber membrane from water solution. *J. Membr. Sci.* **2006**, 278, 373.

[66] H. N. Park, H. A. Choi, S. W. Won, Fibrous polyethylenimine/polyvinyl chloride crosslinked adsorbent for the recovery of Pt (IV) from acidic solution: Adsorption, desorption and reuse performances. *J. Clean. Prod.* **2018**, 176, 360.

[67] S. Liao, C. K. Chan, S. Ramakrishna, Biomimetic nanocomposites for tissue engineering. *J. Bionanosci.* **2007**, 1, 1.

[68] K. Fujiwara, A. Ramesh, T. Maki, H. Hasegawa, K. Ueda, Adsorption of platinum (IV), palladium (II) and gold (III) from aqueous solutions onto l-lysine modified crosslinked chitosan resin. *J. Hazard. Mater.* **2007**, 146, 39.

[69] S. Jafari, B. P. Wilson, M. Hakalahti, T. Tammelin, E. Kontturi, M. Lundström, M. Sillanpää, Recovery of gold from chloride solution by TEMPO-oxidized cellulose nanofiber adsorbent. *Sustainability* **2019**, 11, 1406.

[70] *Recycling – secondary material price indicator*, https://ec.europa.eu/eurostat/statistics-explained/index.php?title=Recycling_%E2%80%93_secondary_material_price_indicator#Average_prices_and_trade_volumes, **2024**.

[71] A. Al Ghatta, J. D. E. T. Wilton-Ely, J. P. Hallett, From sugars to FDCA: a techno-economic assessment using a design concept based on solvent selection and carbon dioxide emissions. *Green Chem.* **2021**, 23, 1716−1733.

[72] R.E. Davis, N. J. Grundl, L. Tao, M. J. Biddy, E. C. Tan, G. T. Beckham, D. Humbird, D. N. Thompson, M. S. Roni, National Renewable Energy Laboratory (NREL), Golden, CO (United States), **2018**. https://doi.org/10.2172/1483234.

[73] L. Shaobo, H. Yuying, J. Liangliang, F. Guang, G. Yiyao, H. Zhiqi, Progress in hydrazine oxidation-assisted hydrogen production. *Energy Reviews* **2025**, 4, 100105.

[74] *Scrap prices-CPU processor chips*, https://rockawayrecycling.com/metal/cpu-processor-chips/, **2024**.

[75] *Hydrochloric acid price data*, https://www.chemanalyst.com/Pricing-data/hydrochloric-acid-61, **2024**.

[76] *Nitric acid price data,* https://www.chemanalyst.com/Pricing-data/nitric-acid-1142, **2024**.

[77] *U.S. Energy Information administration-EIA-Independent Statistics and Analysis*, https://www.eia.gov/, **2024**.

[78] *Metal spot price charts*, https://www.dailymetalprice.com/metalpricecharts.php?c=cu&u=kg&d=2400, **2024**.
